# Comparative Genomics, Whole-Genome Re-sequencing and Expression Profile Analysis of Nucleobase:Cation Symporter 2 (*NCS2*) Genes in Maize

**DOI:** 10.3389/fpls.2018.00856

**Published:** 2018-06-28

**Authors:** Wenbo Chai, Xiaojian Peng, Bin Liu, Jing Wang, Zhan Zhu, Yin Liu, Kai Zhao, Beijiu Cheng, Weina Si, Haiyang Jiang

**Affiliations:** National Engineering Laboratory of Crop Stress Resistance Breeding, School of Life Sciences, Anhui Agricultural University, Hefei, China

**Keywords:** NCS2, evolution, gene duplication, population genetic, expression, abiotic stress, subcellular localization

## Abstract

Nucleobase:cation symporter 2 (NCS2) proteins are important for the transport of free nucleobases, participating in diverse plant growth and developmental processes, as well as response to abiotic stress. To date, a comprehensive analysis of the *NCS2* gene family has not been performed in maize. In this study, we conducted a comparative genomics analysis of *NCS2* genes in 28 plant species, ranging from aquatic algae to land plants, concentrating mainly on maize. Gene duplication events contributed to the expansion of *NCS2* genes from lower aquatic plants to higher angiosperms, and whole-genome/segmental and single-gene duplication events were responsible for the expansion of the maize *NCS2* gene family. Phylogenetic construction showed three *NCS2* subfamilies, I, II, and III. According to homology-based relationships, members of subfamily I are *NCS2/AzgA-like* genes, whereas those in subfamilies II and III are *NCS2/NAT*s. Moreover, subfamily I exhibited ancient origins. A motif compositional analysis showed that one symbolic motif (motif 4) of the *NCS2/NAT* genes was absent in subfamily I. In maize, three *NCS2/AzgA-like* and 21 *NCS2/NAT* genes were identified, and purifying selection influenced the duplication of maize *NCS2* genes. Additionally, a population genetic analysis of *NCS2* genes revealed that *ZmNCS2–21* showed the greatest diversity between the 78 inbred and 22 wild surveyed maize populations. An expression profile analysis using transcriptome data and quantitative real-time PCR revealed that *NCS2* genes in maize are involved in diverse developmental processes and responses to abiotic stresses, including abscisic acid, salt (NaCl), polyethylene glycol, and low (4°C) and high (42°C) temperatures. *ZmNCS2* genes with relatively close relationships had similar expression patterns, strongly indicating functional redundancy. Finally, *ZmNCS2–16* and *ZmNCS2–23* localize in the plasma membrane, which confirmed their predicted membrane structures. These results provide a foundation for future studies regarding the functions of ZmNCS2 proteins, particularly those with potentially important roles in plant responses to abiotic stresses.

## Introduction

Nucleobases have considerable effects on various plant growth and physiological processes (Rapp et al., 2015). The consistent production of new nucleobases provides the necessary components for DNA and RNA synthesis, and nucleobase derivatives are important for cell signaling, nutrition, stress responses, and cell homeostasis (Senecoff et al., 1996). Many indispensable secondary metabolites are nucleobase derivatives, including cytokinins and caffeine. Consistent with the functional importance of nucleobases, pathways associated with their synthesis and catabolism are prevalent in diverse subcellular compartments. Additionally, extensive nucleobase trafficking between these compartments, mediated largely by transporters, is necessary. Several nucleobase transporters have been identified. Equilibrative nucleoside transporter family members are the primary nucleoside transporters in various eukaryotic organisms (Young et al., 2013). Proton symporters (e.g., NCS1), which can transport purines, have been detected in bacteria, fungi, and plants (de Koning and Diallinas, 2000). Two other transporter families, purine permease and ureide permease, have been identified in plants. Furthermore, the nucleobase:cation symporter (NCS2) proteins, which include the nucleobase–ascorbate transporter (NAT) and the AzgA-like protein families, are ubiquitous nucleobase transporters in many organisms (Girke et al., 2014; Niopek-Witz et al., 2014; Vastermark et al., 2015). *NCS2*/*NAT* genes are distantly related to *NCS2*/*AzgA-like* genes (Girke et al., 2014; Niopek-Witz et al., 2014; Vastermark et al., 2015). The NCS2/NAT proteins are important for plant growth and development. These proteins generally contain a NAT signature motif [Q/E/P]-N-X-G-X-X-X-X-T-[R/K/G] and a QH structure, which are critical for NCS2/NAT functions and missing in NCS/AzgA-like genes (Diallinas et al., 1998; Koukaki et al., 2005; Pantazopoulou and Diallinas, 2006).

The NCS2/NAT family proteins transport nucleotides and other specific molecules across membranes. In bacteria, plants, and fungi, NAT proteins are involved in transporting xanthine, uric acid, uracil, and a toxic purine analog. However, in mammals, these proteins transport ascorbate (vitamin C). Many NAT proteins from microorganisms and animals have been studied (Gournas et al., 2008). For example, 10 NCS2/NAT members in *Escherichia coli* have been analyzed, including the uracil transporter UraA (Lu et al., 2011; Li et al., 2014), the xanthine permeases XanQ and XanP (Karatza and Frillingos, 2005), the adenine permease PurP, and the guanine and hypoxanthine permeases YgfQ and YgfU. The latter functions as a high-capacity transporter of uric acid. (Papakostas and Frillingos, 2012; Papakostas et al., 2013). In contrast, relatively few plant NAT (NCS2) proteins have been functionally characterized (Niopek-Witz et al., 2014). The overexpression of *Arabidopsis thaliana AtNAT3* and *AtNAT12* in *E. coli uraA* knockout mutants indicated that they transport adenine, guanine, and uracil with high affinities. Moreover, the transient expression of *AtNAT3* and *AtNAT12* revealed that the encoded proteins are localized in the plasma membrane (Niopek-Witz et al., 2014). In maize (*Zea mays*), leaf permease 1, which is encoded by *ZmLpe1*, is the only functionally characterized NCS2 protein, and it is reportedly required for chloroplast development and membrane integrity (Argyrou and Diallinas, 2001). In *Aspergillus nidulans*, the AzgA-like proteins exist in membranes where they function as proton symporters specific for hypoxanthine, guanine, and adenine (Pantazopoulou et al., 2007). In *Arabidopsis, AtAzg1* and *AtAzg2* can transporter adenine and guanine (Mansfield et al., 2009).

There have been limited investigations into the evolution of the *NCS2* gene family. However, recent developments in genome sequencing technology have resulted in the release of large amounts of plant genome sequences, which may be useful for analyzing the evolution of *NCS2* genes (Schnable et al., 2009). Gene duplication has long been viewed as an important inducer of gene family expansion, especially in higher eukaryotes (Wendel, 2000; Sémon and Wolfe, 2007). Generally, gene duplication modes include whole-genome duplication/segmental duplication (WGD/SD), tandem duplication, and single-gene duplication. Recent genome sequencing studies confirmed that WGD/SD and tandem duplication events were important in duplicating plant genes (Tuskan et al., 2006; Schmutz et al., 2010; Wang et al., 2012). Single-gene duplications involve the relocation of a single gene to a new position, with segregants containing duplicated copies of the gene (Freeling, 2009). Tandem duplications accelerated the expansion of the nucleotide binding site–leucine-rich repeat gene family (Leister, 2004; Kohler et al., 2008), while WGDs contributed to the evolution of the heat-stress factor gene family (Lin et al., 2014). Following gene duplication events, some duplicated genes may be functionally the same as the original gene, while others become pseudogenes that evolve a new function or are deleted because of functional redundancy (Rody et al., 2017).

Globally, maize is an important crop. In this study, we comprehensively analyzed the *NCS2* gene family in maize, as well as in other plant species. We observed that the expansions of the *NCS2* gene families of diverse species, from aquatic algae to land plants, were the result of different types of duplication modes. In maize, WGD/SD and single-gene duplication events have been vital for the expansion of the *NCS2* gene family. A phylogenetic analysis uncovered three *NCS2* gene subfamilies and clarified their evolutionary history among plant species. Non-synonymous (Ka)/synonymous (Ks) substitution ratios for maize paralogous genes indicated that the number of duplicated genes increased under purifying selection. Additionally, the genetic diversity among 78 maize inbred and 22 wild maize lines was assessed using whole-genome sequencing, and tissue-specific *NCS2* gene expression profiles in response to various stresses were examined. Finally, the subcellular localizations of two randomly selected NCS2/NAT proteins were determined. The data presented herein provide new insights into the evolution and functions of maize *NCS2* genes.

## Materials and Methods

### Genome-Wide Identification of *NCS2* Genes

Maize proteomes were downloaded from the MaizeGBD website (version 4). To better trace the origin of the *NCS2* genes in the plant kingdom, complete proteomes of 26 plant species, including *Chondrus crispus, Chlamydomonas reinhardtii, Physcomitrella patens, Selaginella moellendorffii, Amborella trichopoda, Elaeis guineensis, Musa acuminata, Zea mays, Sorghum bicolor, Setaria italic, Brachypodium distachyon, Oryza sativa, Nelumbo nucifera, Solanum tuberosum, Solanum lycopersicum, Vitis vinifera, Medicago truncatula, Glycine max, Fragaria vesca, Malus domestica, Manihot esculenta, Populus trichocarpa, Eucalyptus grandis, A. thaliana, Gossypium raimondii, Theobroma cacao* and *Citrus sinensis* were downloaded from the Phytozome website (Version 11)^1^. The proteome of *Picea asperata* was downloaded from Spruce Genome Project^2^. To better detect the candidate *NCS2* genes, which encode a Xan_ur_permease (PF00860) domain, a pfam_scan perl script in HMMER3.1 was applied to query all of the surveyed proteomes against the Pfam library (Bateman et al., 1999). The molecular weight (Mw) and isoelectric point (pI) of each gene product were estimated using the pI/Mw tool on the ExPASy website^3^ (Gasteiger et al., 2003). The putative transmembrane regions in each maize NCS2 protein were predicted using the default settings of the TMHMM Server (version 2.0)^4^.

### Phylogenetic Analysis and Identification of Conserved Motifs

A phylogenetic species tree was constructed using the Taxonomy Browser online program^5^. The full-length amino acid sequences of all NCS2 proteins were aligned using the MAFFT online program with the auto strategy (Kuraku et al., 2013)^6^. Gaps in aligned sequences were deleted by TrimAL3.0 using -automated1 and -strictplus for maximum-likelihood (ML) and neighbor-joining (NJ) trees, respectively. Then, sequences that completely overlapped others were deleted manually. The resulting alignment file was first used to construct an unrooted phylogenetic tree based on the NJ method in MEGA 7.0 with the JTT mode and pairwise deletion (Kumar et al., 2016). A bootstrap analysis was completed using 1,000 replicates. To construct ML and Bayesian trees, the resulting alignment sequences were submitted to Prottest 3.4 to select best-fit models (Darriba et al., 2011). According to the results, the estimated most appropriate model was the LG+G+F model (with an -lnL 39574.56) based on the Corrected Akaike Information Criterion. We applied this model and other criteria of the Prottest results in PhyML 3.1 to generate a ML tree (Guindon and Gascuel, 2003). We used MrBayes v.3.1.2 to construct a Bayesian tree with the alignment sequences. Because the LG model was not supported in MrBayes and no other models had an Corrected Akaike Information Criterion weight greater than 0.01, two independent 13,000,000-generation runs of five chains using the related WAG model were executed (prset aamodelpr = fixed(WAG); lset rates = gamma; mcmc ngen = 13,000,000; samplefreq = 1000; printfreq = 500; diagnfreq = 5,000; sump burnin = 3,250; contype = allcompat) (Le and Gascuel, 2008). The NJ and ML trees were further edited with MEGA 7.0, while the Bayesian tree was edited in Figtree v1.4.3 (Rambaut, 2012)^7^. Protein sequence motifs were identified using the default settings of the MEME motif search tool^8^.

### Mapping of *ZmNCS2* Genes and Analyses of Gene Duplications

The *ZmNCS2* genes were named based on their positions from the top to the bottom of the chromosomes. The chromosomal positions of the *ZmNCS2* genes were determined based on the information available in the Phytozome database^9^ and were visualized using a Perl script. Duplication events were identified based on sequence alignments. Sequences resulting from SDs were aligned with the ClustalX 2.0 program. The aligned sequences were analyzed using DnaSP6 to estimate the Ks and Ka substitution rates. The divergence time (T) was calculated based on the Ks value and the number of substitutions per synonymous locus per year as follows: T = Ks/2λ × 10^-6^ (where λ = 6.5 × 10^-9^ for grasses).

### Detection of Orthologous Gene Pairs

A Perl script was used to mark the chromosomal positions of the *NCS2* genes in maize, *S. bicolor* (sorghum), and *O. sativa* (rice). The orthologous *NCS2* genes in *A. thaliana*, maize, rice, and sorghum were identified using OrthoMCL^10^. The relationships between orthologous gene pairs among the three species were plotted using Circos^11^.

### Microarray Analysis of *ZmNCS2*

The expression profiles for *ZmNCS2* genes were obtained using publicly available transcriptome data (Stelpflug et al., 2016). Ten *ZmNCS2* genes were used as queries to search the transcriptome data. A heat map was generated using the heatmap function of R^12^.

### Plant Materials and Stress Treatments

Three-week-old seedlings (three-leaf stage) of maize inbred line B73 were used to examine the *ZmNCS2* expression patterns in response to different stress treatments. Maize plants were grown in a greenhouse (14-h light/10-h dark photoperiod; 28 ± 2°C). The treatments were independent exposures to 10 mM abscisic acid (ABA), 4°C, 42°C, 20 mM NaCl, and 20% polyethylene glycol (PEG). Seedlings were irrigated prior to the spray treatments. Leaves harvested 1 h after the treatments were immediately frozen in liquid N_2_ and stored at -80°C for subsequent RNA extractions. Analyses at all developmental stages were completed, with at least three biological replicates per sample.

### RNA Extraction and Quantitative Real-Time PCR (qRT-PCR) Analysis

Total RNA was isolated from each frozen sample using RNAiso Plus (TaKaRa, Japan). The quality of the extracted RNA was assessed in a 1.2% agarose gel. First-strand cDNA was synthesized using 1 μg RNA and the ReverTra Ace qPCR RT Master Mix with gDNA Remover (Toyobo, Japan). A qRT-PCR assay was conducted using the RT-PCR Quick Master Mix. Each reaction was completed in a final volume of 20 μl, containing 10 μl SYBR Green Master Mix, 2.0 μl diluted cDNA sample, and 400 nM gene-specific primers. The Primer Express 3.0 program was used to design gene-specific primers for amplifying 90–150-bp products. Details regarding the primers are provided in **Supplementary Table S1**. The qRT-PCR program was as follows: 95°C for 10 min and 40 cycles at 95°C for 15 s and 60°C for 1 min. The specificities of the reactions were verified by melting curve analyses. The maize actin1 gene was used as an internal reference. The relative mRNA level for each gene was calculated according to the 2^-ΔΔCT^ method. The qRT-PCR assay was conducted at least three times under identical conditions.

### Subcellular Localizations of *ZmNCS2-16* and *ZmNCS2-23*

Plant-mPLoc^13^ was used to predict the subcellular localizations of *ZmNCS2-16* and *ZmNCS2-23*. The *ZmNCS2-16* and *ZmNCS2-23* full-length open reading frames were amplified by PCR using the following gene-specific primer pairs that eliminated the termination codon: *ZmNCS2-16*-F: 5′-ATGGATGAACTATACAAAGGGATGTACCTGCCACATGCTGTTCA-3′ and *ZmNCS2-16*-R: 5′-AACATATCCAGTCACTATGGGGACCGATGGGAAGAACTTATTCA-3′; and *ZmNCS2-23*-F: 5′-ATGGATGAACTATACAAAGGGATGATAATAGTTTCTCTGGTTGC-3′ and *ZmNCS2-23*-R: 5′-AACATATCCAGTCACTATGGGGATGCCGACACACTTTGCC-3′, respectively. After verifying the accuracy of the amplified sequences, the PCR products were independently inserted into the pMDC43-GFP vector using the ClonExpress II One Step Cloning Kit (Vazyme). The *ZmNCS2–16* and *ZmNCS2–23* sequences were placed under the control of the cauliflower mosaic virus 35S promoter. The pMDC43-GFP-*ZmNCS2–16* and pMDC43-GFP-*ZmNCS2–23* plasmids, and the control vector (pMDC43-GFP), were independently inserted into tobacco cells using an *Agrobacterium tumefaciens*-mediated transformation method.

### Calling of Single Nucleotide Polymorphisms (SNPs) and Population Genetic Analysis

We mapped all the reads of maize re-sequencing data (unpublished) against the reference maize v3.0 genome by BWA using default parameters(Li and Durbin, 2009). Picard-MarkDuplicates^14^ and GATK – IndelRealigner were used to correct mapping results(Mckenna et al., 2010). Finally, GATK-UnifiedGenotyper was utilized to detect SNPs in each maize individual at corresponding gene loci. Nucleotide variation was estimated as the ratio between SNP numbers and corresponding CDS length. Pair-wise fixation index (Fst) and Tajima’ *D* were calculated by Arlequin31(Excoffier and Lischer, 2010).

## Results

### Identification of *NCS2* Genes in Maize and Other Species

Twenty-four candidate *NCS2* genes were identified in maize based on a search of the Pfam database (**Table 1**). We named all *NCS2* genes from *ZmNCS1–1* to *ZmNCS2–24* according to their positions on the chromosomes. These *ZmNCS2* genes were distributed unevenly among nine chromosomes, with none on chromosome 9. Chromosomes 1, 2, and 7 each had four *ZmNCS2* genes, while chromosomes 4 and 8 each had two and chromosomes 3, 5, 6, and 10 each had one chromosome. The lengths of the encoded proteins ranged from 192 to 795 amino acids, with an average of 468 amino acids. The Mw of these proteins ranged from 21 to 85.4 kDa, while the pI values were between 6.4 and 9.61.

**Table 1:** Details of the 24 *ZmNCS2* genes and their encoded proteins.

Gene ID	Gene name	Type	The length of the gene	Chromosome location	AA	PI
Zm00001d030868	ZmNCS2-1	NCS2/NAT	164.19661	1	556	9.13
Zm00001d032322	ZmNCS2-2	NCS2/NAT	222.263901	1	533	9.29
Zm00001d033585	ZmNCS2-3	NCS2/NAT	267.905764	1	527	9.01
Zm00001d034678	ZmNCS2-4	NCS2/NAT	299.619441	1	529	9.54
Zm00001d005590	ZmNCS2-5	NCS2/NAT	180.005803	2	525	9.08
Zm00001d006408	ZmNCS2-6	NCS2/NAT	206.925694	2	733	9.18
Zm00001d007116	ZmNCS2-7	NCS2/NAT	222.533458	2	461	7.7
Zm00001d007251	ZmNCS2-8	NCS2/AzgA-like	225.688832	2	543	8.85
Zm00001d000434	ZmNCS2-9	NCS2/AzgA-like	0.046642	3	573	6.6
Zm00001d042322	ZmNCS2-10	NCS2/NAT	161.217464	3	225	8.73
Zm00001d048960	ZmNCS2-11	NCS2/AzgA-like	10.883172	4	547	8.95
Zm00001d049324	ZmNCS2-12	NCS2/NAT	26.332755	4	522	8.65
Zm00001d053604	ZmNCS2-13	NCS2/NAT	236.138479	4	192	9.24
Zm00001d053955	ZmNCS2-14	NCS2/NAT	244.179086	4	382	8.74
Zm00001d012935	ZmNCS2-15	NCS2/NAT	2.204358	5	519	9.56
Zm00001d018001	ZmNCS2-16	NCS2/NAT	211.694641	5	326	9.54
Zm00001d035993	ZmNCS2-17	NCS2/NAT	65.161869	6	218	8.46
Zm00001d020345	ZmNCS2-18	NCS2/NAT	108.133164	7	530	9.37
Zm00001d021163	ZmNCS2-19	NCS2/NAT	144.473134	7	246	9.61
Zm00001d021519	ZmNCS2-20	NCS2/NAT	154.989542	7	795	8.26
Zm00001d022079	ZmNCS2-21	NCS2/NAT	169.619486	7	245	8.78
Zm00001d012307	ZmNCS2-22	NCS2/NAT	172.160772	8	701	9.61
Zm00001d012693	ZmNCS2-23	NCS2/NAT	178.730558	8	524	8.73
Zm00001d024918	ZmNCS2-24	NCS2/NAT	94.913396	10	281	6.4

**FIGURE 1:**
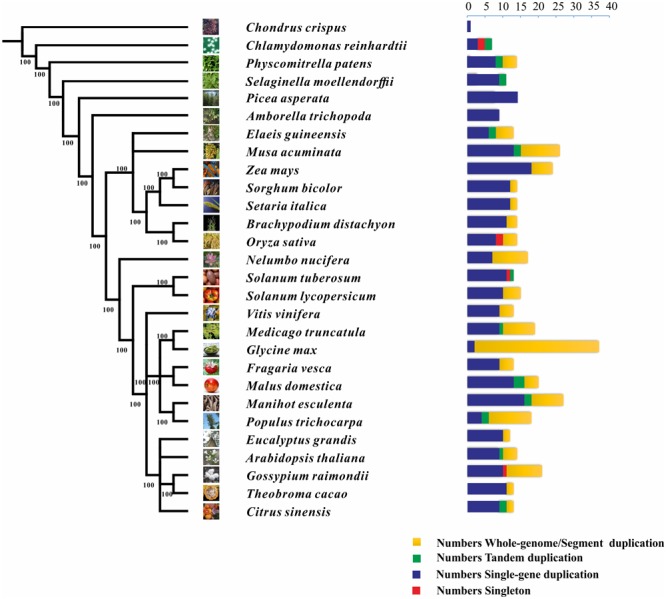
Phylogenetic relationships among 26 species and the duplication modes of the *NCS2* gene family. Green, red, yellow, and blue represent tandem, singleton, whole-genome/segmental, and single-gene duplication modes, respectively.

We also characterized the *NCS2* homologs from 27 other plant species, ranging from single-celled aquatic plants to higher angiosperms (**Figure 1** and **Table 2**). A total of 441 *NCS2* genes were identified, with 1–37 per species (Stelpflug et al., 2016). Additionally, only one and seven homologs were detected in the algae *C. crispus* and *C. reinhardtii*, respectively. In contrast, 11 and 14 *NCS2* homologs were detected in the basal land plant species *P. patens* and *S. moellendorffii*, respectively. Additionally, 14 *NCS2* homologs were found in *P. asperata*, which is a gymnosperm, and 9 *NCS2* homologs were detected in *A. trichopoda*, which is an ancestor of the flowering plant lineage. The number of *NCS2* genes in angiosperms ranged from 9 to 37, with considerable gene expansion observed in several species. Specifically, *M. acuminata, Z. mays*, and *G. max* contained as many as 26, 24, and 37 *NCS2* genes, respectively. Finally, an analysis of the linear correlations between the numbers of *NCS2* genes and the total numbers of genes in the genomes of all surveyed species revealed a weak association (*R*^2^ = 0.6642, *p* = 5.9327E-15; **Supplementary Figure S1**). Thus, the number of *NCS2* genes was not proportional to the number of gene loci, which may be a consequence of the evolution of the *NCS2* gene family.

**Table 2:** Details of the 28 analyzed species.

Species	Abbreviation	Number	Dicot/Monocot	Genome(Mb)	Reference	Gene
*Chondrus crispus*	Ccr	1	Chondrus	104.98	Janouškovec et al., 2013	9807
*Chlamydomonas reinhardtii*	Cre	7	Chlorophyta	120.405	Merchant et al., 2007	14488
*Physcomitrella patens*	Ppa	14	Moss	477.948	Rensing et al., 2008	35934
*Selaginella moellendorffii*	Smo	11	Fern	212.502	Banks et al., 2011	34746
*Picea asperata*	Pas	15	Pinaceae	11961.4	Nystedt et al., 2013	
*Amborella trichopoda*	Atr	9	Amborella	706.495	Amborella Genome Project, 2013	31494
*Elaeis guineensis*	Egu	13	Monocot	1017.1	Yeoh et al., 2013	41887
*Musa acuminata*	Ma	26	Monocot	472.231	D’Hont et al., 2012	47707
*Zea mays*	Zm	24	Monocot	2155.82	Schnable et al., 2009	58291
*Sorghum bicolor*	Sb	14	Monocot	709.345	Bowers et al., 2009	39248
*Setaria italica*	Si	14	Monocot	441.705	Doust and Bennetzen, 2009	35844
*Brachypodium distachyon*	Bd	14	Monocot	243.424	Vogel et al., 2010	33944
*Oryza sativa*	Os	14	Monocot	368.351	Yu et al., 2002	36376
*Nelumbo nucifera*	Nnu	17	Dicot	797.494	Ming et al., 2013	38191
*Solanum tuberosum*	St	13	Dicot	705.934	Xu et al., 2011	37966
*Solanum lycopersicum*	Sly	15	Dicot	760.067	Tomato Genome Consortium, 2012	36010
*Vitis vinifera*	Vv	13	Dicot	486.197	Jaillon et al., 2007	41208
*Medicago truncatula*	Mtr	19	Dicot	412.924	Young et al., 2011	57661
*Glycine max*	Gm	37	Dicot	953.339	Schmutz et al., 2010	71525
*Fragaria vesca*	Fve	13	Dicot	214.373	Shulaev et al., 2011	31387
*Malus domestica*	Mdo	20	Dicot	1288.87	Velasco et al., 2010	60549
*Manihot esculenta*	Mes	27	Dicot	390.836	Velasco et al., 2010	43286
*Populus trichocarpa*	Pt	18	Dicot	417.287	Tuskan et al., 2006	45942
*Eucalyptus grandis*	Eg	12	Dicot	691.43	Tuskan, 2014	52554
*Arabidopsis thaliana*	At	14	Dicot	116.846	David et al., 2008	39593
*Gossypium raimondii*	Gra	21	Dicot	767.667	Wang et al., 2012	59057
*Theobroma cacao*	Tca	13	Dicot	335.437	Motamayor et al., 2013	37520
*Citrus sinensis*	Csi	13	Dicot	323.528	Xu et al., 2013	43683

### Duplication Modes Involved in the Expansion of the *NCS2* Gene Family

Diverse modes of gene duplication, especially WGDs and tandem duplications, were important in increasing the number of early diverging groups of land plants (Du et al., 2015). The duplication modes associated with the *NCS2* genes were analyzed to characterize the expansion and variability of *NCS2* homologs in the surveyed plants. Duplicated types of NCS2 genes were elucidated from the PLAZA website^15^. The *NCS2* genes underwent WGD and/or tandem duplication events in all of the surveyed plant species, with the exception of *C. crispus, P. asperata*, and *A. trichopoda*. In *G. max*, which contained the greatest number of *NCS2* genes, 33 of 35 *NCS2* homologs were generated from WGD events. Tandem duplications were important for the expansion of the *NCS2* gene family in some species, including *S. lycopersicum, C. reinhardtii*, and *S. moellendorffii*. Additionally, WGD events were critical for *NCS2* gene family expansion in species such as *N. nucifera, G. max*, and *G. raimondii*. Furthermore, tandem duplication and WGD events contributed to the expansion of the *NCS2* gene family in some species, including *A. thaliana, P. patens, E. guineensis, M. acuminata*, and *P. trichocarpa*. An analysis of the *NCS2* gene family’s expansion in maize revealed a lack of tandem duplication events. However, six *NCS2* genes resulted from WGD events and 18 *NCS2* genes were the product of single-gene duplications (Paterson, 2012). Our results indicated that WGD (or SD) events and single-gene duplications were primarily responsible for the expansion of the *ZmNCS2* family.

**FIGURE 2:**
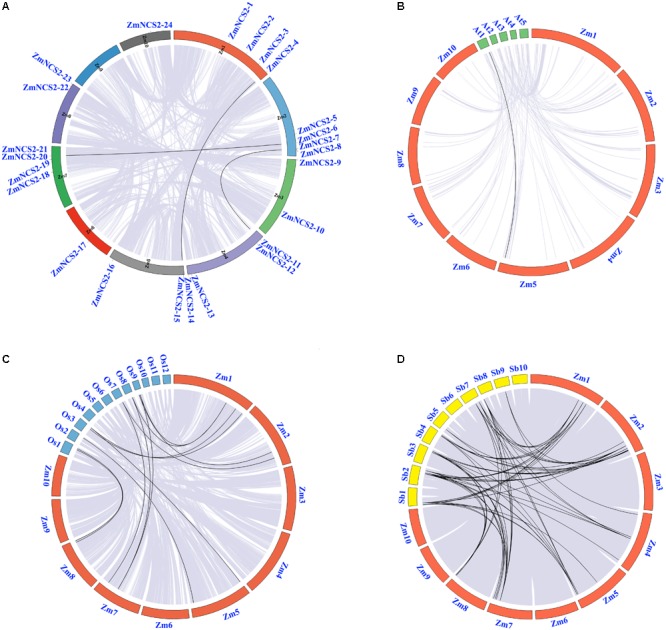
Microsyntenic relationships among the *NCS2* regions in *Zea mays, Sorghum bicolor*, and *Oryza sativa*. **(A)** Chromosomal locations of *ZmNCS2* genes and WGD paralogous genes in maize. **(B–D)** *Zea mays* (Zm), *Sorghum bicolor* (Sb), and *Oryza sativa* (Os) chromosomes are presented in different colors. Black lines represent syntenic relationships between *NCS2* regions.

We also investigated the chromosomal synteny among maize *NCS2* genes and in three other plant species (**Figure 2**). A comparative analysis was used to identify orthologous *NCS2* genes among *A. thaliana*, maize, rice, and sorghum. We identified 69 syntenic gene pairs between maize and sorghum and 12 syntenic gene pairs between maize and rice (**Figure 2**), suggesting that maize is more closely related to sorghum than to rice (Tikhonov et al., 1999). These results are also consistent with an earlier study, which concluded that sorghum and maize diverged 11.9 million years ago, while rice diverged from the common ancestor of maize and sorghum 50 million years ago (Lai et al., 2004). Interestingly, we detected only one collinear gene pair between *A. thaliana* and maize.

### Phylogenetic Analysis of *NCS2* Genes in Different Species

**FIGURE 3:**
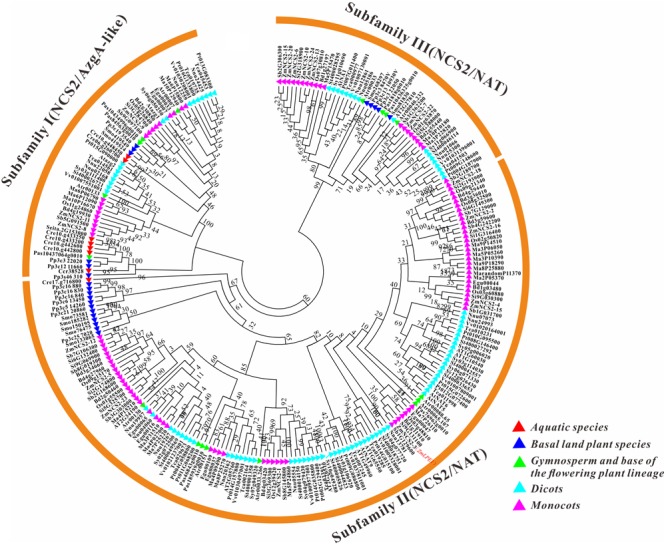
Phylogenetic analysis of *NCS2* genes in 20 species. A phylogenetic tree containing proteins encoded by all of the *NCS2* genes from 20 species was constructed using the neighbor-joining method after the full-length protein sequences were aligned. Red: aquatic plants (*Chondrus crispus* and *Chlamydomonas reinhardtii*); blue: early diverging plants (*Physcomitrella patens* and *Selaginella moellendorffii*); green: gymnosperm (spruce) and a flowering plant (*Amborella trichopoda*); light blue: dicots (*Theobroma cacao, Arabidopsis thaliana, Nelumbo nucifera, Solanum lycopersicum, Vitis vinifera, Populus trichocarpa*, and *Solanum tuberosum*); and purple: monocots (*Musa acuminata, Elaeis guineensis, Zea mays, Sorghum bicolor, Setaria italica, Brachypodium distachyon*, and *Oryza sativa*).

To clarify the evolutionary history of *NCS2* homologs in different plant lineages, we examined the phylogenetic relationships among 255 *NCS2* genes from 20 species representing most of the plant kingdom. These included two aquatic species (*C. crispus* and *C. reinhardtii*), one moss (*P. patens*), one fern (*S. moellendorffii*), one gymnosperm (*P asperata*), one Amborellales (*A. trichopoda)*, seven monocots (*B. distachyon, O. sativa, S. italica, S. bicolor, Z. mays, E. guineensis*, and *M. acuminata*) and seven dicots (*A. thaliana, S. tuberosum, S. lycopersicum, T. cacao, V vinifera, P. trichocarpa*, and *N. nucifera*). Unrooted phylogenetic trees were constructed based on the NJ, ML and Bayesian methods (**Figure 3, Supplementary Figure S2**, and **Supplementary Table S2**, respectively). Because of the similarity in the tree topologies, only the NJ phylogenetic tree was used for further analysis. According to the topological relationships and bootstrap values, our phylogenetic tree comprised three subfamilies (**Table 3**), each with a different number of *NCS2* homologs. Subfamilies I and III consisted of 54 and 47 genes, respectively, while subfamily II included as many as 154 genes (**Table 3**), indicative of considerable gene expansion. In particular, based on the homology levels of *NCS2* genes with functionally characterized members, two identified *NCS2/AzgA-like* genes, *AtAzg1* and *AtAzg2*, clustered within subfamily I, and the genes in subfamily I belonged to the *NCS2/AzgA-like* family. All of the identified *NCS2/NAT* genes clustered within subfamilies II and III, and their members were further classified as *NCS/NAT* genes (**Supplementary Table S3**). Additionally, the *C. crispus* (red alga) *NCS2* gene was included in subfamily I, while the *C. reinhardtii* (green alga) *NCS2* genes clustered in subfamilies I and II. All of the remaining analyzed species possessed *NCS2* genes from all three subfamilies. These data were consistent with the ancient origins of the subfamily I *NCS2/AzgA-like* homologs and the more recent origin of the subfamily III *NCS2/NAT* members. Moreover, the fact that subfamily II contained the most *NCS2* genes suggested that recent duplications occurred after species diverged from a common ancestor.

**Table 3:** The istribution of *NCS2* genes in different subfamilies.

Species	Subfamily I	Subfamily II	Subfamily III
*Chondrus crispus*	1	0	0
*Chlamydomonas reinhardtii*	6	1	0
*Selaginella moellendorffii*	2	5	2
*Physcomitrella patens*	4	7	3
*Picea asperata*	3	3	2
*Amborella trichopada*	2	5	2
*Arabidopsis thaliana*	2	10	2
*Solanum tuberosum*	1	9	2
*Solanum lycopersicum*	2	11	2
*Theobroma cacao*	3	7	2
*Nelumbo nucifera*	4	8	4
*Vitis vinifera*	2	7	2
*Populus trichocarpa*	3	11	3
*Brachypodium distachyon*	2	9	2
*Oryza sativa*	3	9	2
*Setaria italica*	2	10	2
*Sorghum bicolor*	2	10	2
*Zea mays*	3	10	8
*Elaeis guineensis*	2	6	2
*Musa acuminata*	5	16	3

**FIGURE 4:**
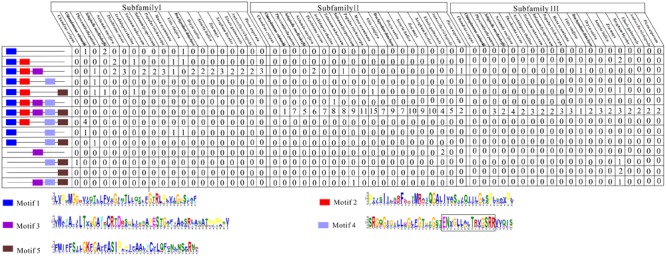
Conserved motifs of NCS2 family proteins in 20 species. Conserved protein motifs in NCS2 proteins were identified using the MEME program. Motifs are indicated with different colors. Motif 4 includes the core region of the *NCS2/NAT* domain “ENXGLLGLTR”.

Subsequently, we examined the motif compositions of the NCS2 proteins encoded by genes in different clades. Five putative motifs appeared to correspond to a typical NCS2 domain (xanthine/uracil/vitamin C permease) (**Figure 4**). Motifs 1 and 4 were annotated with the xanthine/uracil/vitamin C permease domains, which are associated with transporter activities. Additionally, motif 4 included the core region of the *NCS2/NAT* domain “ENXGLLGLTR.” The NCS2 proteins clustered within the same subfamily shared similar motif compositions. Almost all of the subfamily I NCS2/AzgA-like proteins from land plants contained only motifs 1, 2, and 3, which is consistent with previous studies (Amillis and Koukaki, 2001; Cecchetto et al., 2004; Mansfield et al., 2009). The *C. crispus* NCS2 protein contained motifs 4 and 5. Most of the proteins in subfamilies II and III harbored all five motifs and a complete *NCS2/NAT* domain.

### Maize *NCS2* Gene Evolution Was Driven by Purifying Selection

**FIGURE 5:**
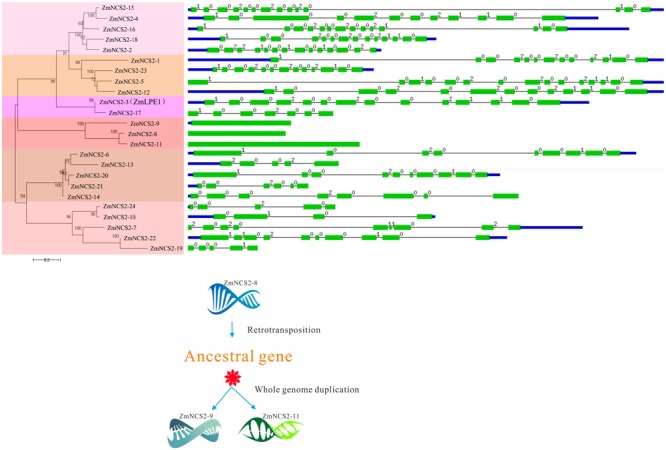
Phylogenetic and gene structural analyses of the maize *NCS2* family. Phylogenetic tree of all *Zea mays NCS2* genes, intron/exon structures of 24 *NCS2* genes, and duplication of an intronless gene caused by retrotransposition.

The molecular evolution rate was estimated to further characterize the evolution of the maize *NCS2* genes. The ratio of Ka to Ks substitutions is an essential parameter of molecular evolution. A Ka/Ks value greater than 1 generally indicates positive selection, while a value less than 1 indicates negative or purifying selection. We calculated the Ka/Ks values for the *Z. mays* paralogs. For a thorough examination of maize paralog pairs, we constructed an NJ tree based only on maize proteins (**Figure 5**). Genes with more than 50% homology were considered to be paralog pairs. Twenty-eight maize paralog pairs were identified, and their Ka/Ks values (**Table 4** and **Supplementary Figure S3**) were less than 1, corresponding to a strong purifying selection. The average Ka/Ks value (0.0432) of the WGD pairs was much lower than that of the single-gene duplication pairs (0.218). However, a sliding-window analysis indicated that some Ka/Ks values were greater than 1, which was consistent with positive selection (**Supplementary Figure S3**). For example, the Ka/Ks value of the *ZmNCS2–8/11* pair was only 0.0409, but two regions in these genes had high Ka/Ks values, indicating the regions were under positive selection. Finally, based on a substitution rate of 6.5 × 10^-9^ substitutions per locus per year in grass species, the duplication events associated with 28 paralog pairs were estimated to have occurred 4.6–486 million years ago.

**Table 4:** Ka/Ks values and the estimated divergence times for the duplicated *NCS2* paralogs in *Zea mays.*

Paralogous pairs	Ks	Ka/Ks	T(Mya)
ZmNCS2-4/16	2.0975	0.1861	322.7
ZmNCS2-15/16	1.9726	0.1357	303.5
ZmNCS2-2/18	1.1503	0.113	177.0
ZmNCS2-1/23	1.6788	0.3361	258.3
ZmNCS2-1/5	3.1584	0.3302	485.9
ZmNCS2-1/12	2.9285	0.3603	450.5
ZmNCS2-23/5	1.6428	0.1272	252.7
ZmNCS2-23/12	1.8148	0.1653	279.2
ZmNCS2-5/12	1.3158	0.1244	202.4
ZmNCS2-3/17	0.0437	0.0335	6.7
ZmNCS2-9/8	0.6875	0.4016	105.8
ZmNCS2-9/11	0.6946	0.3917	106.9
ZmNCS2-6/13	0.6123	0.4221	94.2
ZmNCS2-6/21	0.1653	0.0341	25.4
ZmNCS2-6/14	0.1929	0.0358	29.7
ZmNCS2-20/13	0.5587	0.4157	86.0
ZmNCS2-20/21	0.0286	0.0153	4.4
ZmNCS2-20/14	0.03	0.0241	4.6
ZmNCS2-13/21	0.5257	0.4143	80.9
ZmNCS2-13/14	0.5346	0.4384	82.2
ZmNCS2-21/14	0.0203	0.0399	3.1
ZmNCS2-7/22	0.3658	0.379	56.3
ZmNCS2-7/19	0.4235	0.4177	65.2
ZmNCS2-22/19	0.0736	0.0885	11.3
ZmNCS2-10/24	0.0305	0.0301	4.7
ZmNCS2-4/15	0.26	0.0703	40.0
ZmNCS2-6/20	0.2014	0.0184	31.0
ZmNCS2-8/11	0.1634	0.0409	25.1

### Population Genetic Analysis of *NCS2* Genes in 78 Inbred and 22 Wild Maize Lines

Genome resequencing data for 78 inbred and 22 wild maize lines were used to investigate *NCS2* gene polymorphisms (data not published). The average nucleotide divergence of *NCS2* genes in the wild lines was greater than that in the inbred lines. Additionally, for all *NCS2* genes, nucleotide divergence was greater in the wild lines than in the inbred lines, implying that the *NCS2* genes in the inbred lines were relatively conserved (**Table 5**). Additionally, the fixation index (Fst) values were calculated to determine the genetic differentiation between the inbred and wild maize populations, which was influenced by several evolutionary processes, including genetic drift and/or natural selection. Most of the Fst values were close to 0, with only *ZmNCS2–2, ZmNCS2–19, ZmNCS2–20*, and *ZmNCS2–21* having Fst values approaching 1 (i.e., greater genetic differentiation). Finally, Tajima’s *D* value was used to estimate the evolutionary dynamics. Values of 0, >0, and <0 indicated the selection pressure of a sudden population contraction and a population expansion after a recent bottleneck, respectively. Tajima’s *D* values in the wild maize lines were <0 for all *NCS2* genes, except for *ZmNCS2–8*, which had a Tajima’s *D* value of 0. In the inbred maize lines, Tajima’s *D* value was <0 for *ZmNCS2–2, –12, –15, –16*, –*18*, and –*21*, but >0 for *ZmNCS2–1, –3, –4, –5, –8, –11, –19, –20*, and *–23.*

**Table 5:** Summary of Tajima’s *D*, Fst, and Pi values in 78 inbred and 22 wild maize lines.

Name	Tajima’s D		Pi		Fst
	Inbred	Wild	Inbred-Pi	Wild-Pi	
ZmNCS2-5	0.97643	–0.00834	0.006	0.007	0.13213^∗∗∗^
ZmNCS2-4	1.55339	–0.75836	0.008	0.009	0.0558^∗∗^
ZmNCS2-12	–0.9032	–0.54566	0.005	0.006	0.10359^∗∗^
ZmNCS2-2	–0.91075	–1.55056^∗^	0	0.007	0.62528^∗∗^
ZmNCS2-15	–0.33853	–0.15618	0.004	0.007	0.07661^∗∗^
ZmNCS2-18	–0.07269	–0.59671	0.001	0.034	0.2163^∗∗∗^
ZmNCS2-23	0.81477	–1.27716	0.004	0.004	0.06692^∗∗^
ZmNCS2-16	–0.80634	–1.66656^∗^	0.003	0.003	0.00693
ZmNCS2-3	1.2244	–2.09018^∗∗^	0.003	0.004	0.13131^∗∗∗^
ZmNCS2-1	0.04933	–1.66731	0.002	0.003	0.03633^∗^
ZmNCS2-8	0.13593	0	0.003	0.003	–0.02427
ZmNCS2-22	0.35068	–2.03415^∗∗^	0.005	0.006	0.02822
ZmNCS2-11	0.30145	–1.55056^∗^	0.002	0.003	0.12779^∗∗∗^
ZmNCS2-21	–0.29227	–0.22349	0	0.016	0.92549^∗∗∗^
ZmNCS2-19	0.78984	–1.19389^∗^	0.001	0.036	0.79087^∗∗∗^
ZmNCS2-20	1.56053	–0.6273	0.004	0.008	0.57721^∗∗∗^

*^∗^ means *P* values below 0.1, ^∗∗^ means *P* values below 0.05, ^∗∗∗^ means *P* values below 0.01.*

### Expression Characteristics of *ZmNCS2* Genes Based on Transcriptome and qRT-PCR Analyses

**FIGURE 6:**
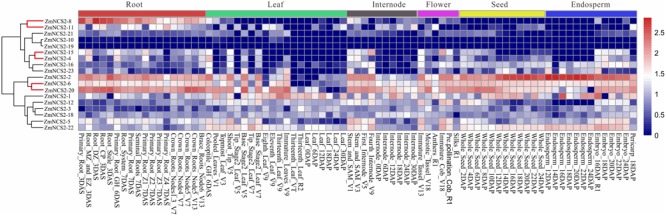
Expression profiles of *NCS2* genes in different tissues. Gene expression was analyzed in the roots, leaves, internodes, flowers, seeds, and endosperm. Genome-wide microarray data were obtained from the NimbleGen microarray provided by Stelpflug et al. (2016).

To gain insights into their possible functions, we comprehensively examined the expression patterns of all *ZmNCS2* genes using microarray data and a qRT-PCR analysis. We first analyzed the *ZmNCS2* expression profiles in different tissues based on published data for 18 *NCS2* genes (Stelpflug et al., 2016) (**Figure 6**). Genes clustered together with similar expression profiles belonged to the same clade in the phylogenetic tree. For example, the expression levels of *NCS2–10, NCS2–19*, and *NCS2–21*, which belonged to subfamily III, were almost undetectable in different maize tissues. Additionally, four members of subfamily II (*NCS2–15, NCS2–4, NCS2–16*, and *NCS2–23*) were expressed more highly in the roots than in the leaves. The three WGD pairs exhibited similar expression profiles. We observed that *NCS2–6* and *NCS2–20* were highly expressed in all surveyed tissues, while *NCS2–8* and *NCS2–11* were highly expressed in the roots, but were seldom expressed in the leaves, internodes, flowers, seeds, or endosperm. Differences in the *ZmNCS2* expression patterns suggested that the encoded proteins may exhibit diverse or novel functions, which is consistent with the results of the phylogenetic and protein motif analyses.

**FIGURE 7:**
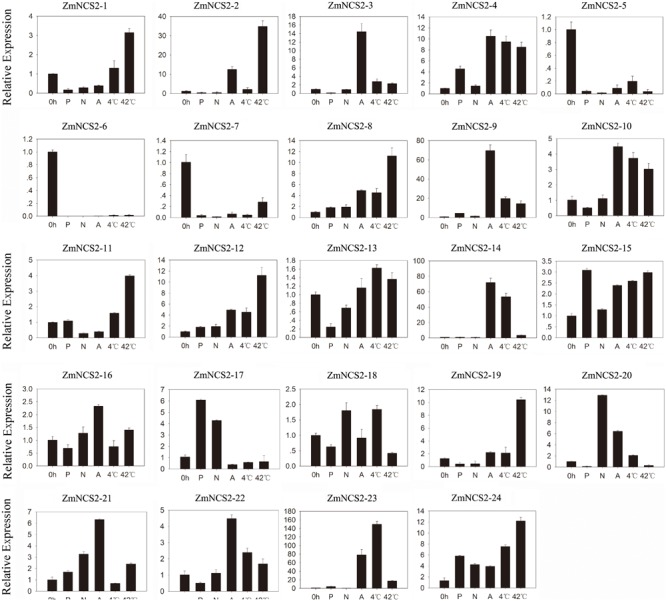
Expression patterns of five stress-responsive *ZmNCS2* genes in response to various stress treatments. Relative expression levels and stress treatments (PEG, ABA, NaCl, 4°C, and 42°C) are indicated on the *y*-axis and *x*-axis, respectively.

Nucleobase derivatives are critical for cell signaling, nutrition, stress responses, and cell homeostasis. Additionally, soil salinity can decrease L-ascorbic acid contents in wheat (Shalata et al., 2001; Sairam and Srivastava, 2002). Therefore, elucidating the regulatory pathways involved in stress adaptations may be useful for improving crop production. We further investigated the *ZmNCS2* expression levels in response to abiotic stresses by treating leaves of 3-week-old seedlings with ABA, salt, simulated drought, cold, and heat stresses (**Figure 7**). The analyzed genes were differentially expressed in the leaves under different abiotic stress conditions. The null treatment (0 h) was normalized to an expression level of 1. The *ZmNCS2–5* and *ZmNCS2–15* expression levels were considerably down-regulated under all abiotic stresses, while the *ZmNCS2–12* expression level was significantly up-regulated in response to all treatments. Under drought conditions, the *ZmNCS2* genes were not expressed or were expressed at low levels, except for *ZmNCS2–4* and *ZmNCS2–12*, which had up-regulated expression levels following the PEG treatment. Additionally, the expression levels of some of the *NCS2* genes were strongly up-regulated during the heat stress treatment (*ZmNCS2–1, ZmNCS2–2, ZmNCS2–4, ZmNCS2–12*, and *ZmNCS2–16*), while the expression levels of other *NCS2* genes were up-regulated by ABA (*ZmNCS2–2, ZmNCS2–3, ZmNCS2–4, ZmNCS2–12, ZmNCS2–16*, and *ZmNCS2–23*). Furthermore, the expression levels of *ZmNCS2–4* and *ZmNCS2–23* were sensitive to the 4°C treatment.

### Subcellular Localizations of *ZmNCS2–16* and *ZmNCS2–23*

**FIGURE 8:**
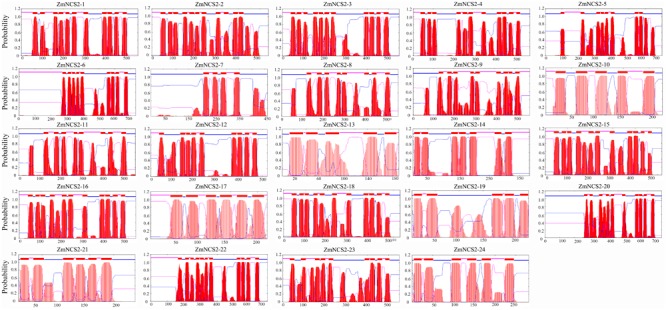
Predicted transmembrane regions of ZmNCS2 proteins. Transmembrane regions were predicted using the TMHMM Server (version 2.0) (http://www.cbs.dtu.dk/services/TMHMM/).

**FIGURE 9:**
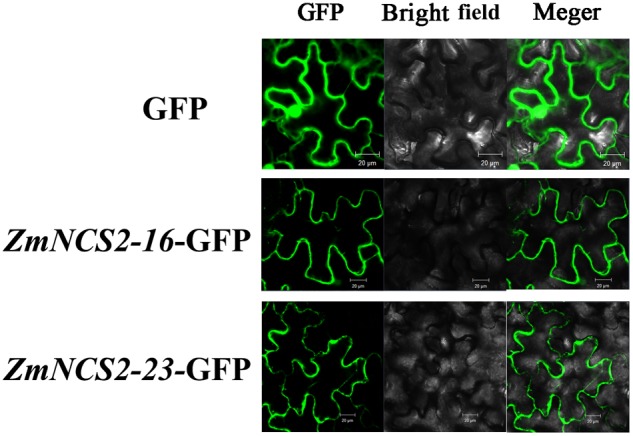
Subcellular localization of *ZmNCS2–16* and *ZmNCS2–23.* The control (GFP alone) signal was detected throughout the cell, while the *ZmNCS2–16*-GFP and *ZmNCS2–23*-GFP signals were localized to the plasma membrane.

The NCS2 proteins are highly hydrophobic. The number of putative membrane-spanning segments was calculated using the TMHMM Server (version 2.0). We predicted that maize NCS2 proteins include 5–11 membrane-spanning helices (**Figure 8**). We randomly selected two genes (*ZmNCS2–16* and *ZmNCS2–23*) to determine whether the encoded proteins were present in the cell membrane. An *in silico* analysis of the *NCS2* gene family indicated that *ZmNCS2–16* and *ZmNCS2–23* were localized in the cell membrane^16^. To experimentally confirm this prediction, we transiently expressed *ZmNCS2–16*-GFP and *ZmNCS2–23*-GFP under the control of the cauliflower mosaic virus 35S promoter in tobacco cells, which revealed that they were present in the cell membrane (**Figure 9**).

## Discussion

Members of the *NCS2* family have critical functions regarding the transport of free nucleobases (purines and pyrimidines). Despite the intriguing functional diversity and broad expansion of this gene family during evolution (de Koning and Diallinas, 2000; Frillingos, 2012), a genome-wide study of *NCS2/NAT* homologs has been reported for only a few species, including *A. thaliana, S. lycopersicum*, and *M. domestica* (Maurino et al., 2006; Cai et al., 2014; Sun et al., 2016). In the present study, we conducted a comprehensive investigation of the evolution and expression patterns of maize *NCS2/NAT/AzgA-like* superfamily genes. In total, 24 *ZmNCS2* were found in *Z. mays*. Additionally, *NCS2* genes were also detected in 27 other plant species, including two aquatic chlorophytes (*C. crispus* and *C. reinhardtii*), two early diverging land plant species (*P. patens* and *S. moellendorffii*), one gymnosperm (*P. asperata*), and one Amborellales (*A. trichopoda*), as well as 23 monocots and dicots. A total of 441 *NCS2* genes were identified, implying that *NCS2* homologs are evolutionarily conserved in the plant kingdom. We observed varying copy numbers among *NCS2* homologs and a considerable expansion of the corresponding gene family from lower aquatic plants to angiosperms. The greater number of *NCS2* genes in land plants than in *C. crispus* and *C. reinhardtii* suggested that the expansion of the gene families during speciation was the result of different gene duplication modes (i.e., WGD/SD, tandem duplication, and single-gene duplication). An examination of the duplication modes associated with *NCS2* genes revealed that one or more duplication modes were primarily responsible for the expansion of the plant *NCS2* gene families. We also attempted to trace the evolutionary history of *NCS2* genes among diverse plant species, from a red alga to angiosperms. Three subfamilies were identified based on the phylogenetic analyses. According to the distribution of previously functionally characterized maize *NCS2* genes, we found that *AzgA* genes clustered only within subfamily I, while all of the previously reported *NAT* genes clustered within subfamilies II and III. *NCS2* genes in subfamily I were classified as *NCS2/AzgA-like* genes, while those in subfamilies II and III were classified as *NCS2/NAT* genes. There were three *ZmNCS2/AzgA-like* and 21 *ZmNCS2/NAT* genes (**Table 1**). All of the surveyed species contained at least one subfamily I *NCS2* gene, suggesting the ancient origins of these genes. In contrast, subfamily III *NCS2* genes were present only in land plant species, indicating that these *NCS2* genes diverged from the early diverging land plants, such as *P. patens* and *S. moellendorffii*, which represent ancient lineages that diverged from aquatic organisms. Furthermore, subfamily II contained the greatest number of *NCS2* genes, possibly because of recent duplication events that resulted in neofunctionalization. An examination of motifs revealed that NCS2 proteins from different subfamilies had conserved and analogous structural features. The typical NCS2 domain may be represented by five putative motifs. The proteins encoded by the *NCS2* genes in subfamily I contain motifs 1, 2, and 3, while proteins encoded by subfamily II and III genes contain motifs 1–5, which is consistent with the phylogenetic results. Based on the deduced origins of these three subfamilies, that ancient *NCS2/NAT* domains may have been relatively short and harbored only a few motifs. During evolution, gene fusions or chromosomal recombination occurred that enlarged the NCS2 domain, ultimately producing the typical domain with five motifs.

In maize, WGD and single-gene duplication events, but not tandem duplications, were likely important for the *NCS2* gene family’s expansion. Moreover, the intronless structure of all the three maize *NCS2/AzgA-like* (*ZmNCS2–9, ZmNCS2–8*, and *ZmNCS2–11*) genes implied that a retrotransposition duplication of *ZmNCS2–9* resulted in the ancestral gene of *ZmNCS2–8* and *ZmNCS2–11*, which were then generated by a WGD (**Figure 5**). Paralogous maize *NCS2* gene pairs were identified, and the corresponding Ka/Ks values revealed that purifying selection may have been largely responsible for the increase in the functional diversity of the *NCS2* gene family. Moreover, the Ka/Ks values were lower for the WGD gene pairs than for the single-gene duplication gene pairs. The duplication of three paralogous gene pairs was estimated to have occurred 4.6–486 million years ago. A phylogenetic analysis revealed that the genes from the monocot and dicot species clustered separately, suggesting that the expansion of the *NCS2* gene family differed between monocotyledons and dicotyledons. A previous comparative genomics study concluded that euchromatic regions are highly conserved between rice and maize (Wei et al., 2007). We observed that the number of orthologous genes was greater between maize and sorghum than between maize and rice, which is consistent with previous studies that suggested sorghum and maize progenitors diverged after rice diverged from the common ancestor of maize and sorghum. Our results may be useful for clarifying the evolution of the *NCS2* multigene families in different species.

Plant growth and productivity are frequently threatened by abiotic stresses, including drought, high salinity, and extreme temperatures. The expression levels of many stress-related genes may be induced by abiotic stresses. Our qRT-PCR analysis confirmed that *ZmNCS2* genes are differentially expressed in response to abiotic stresses. An earlier study concluded that drought stress can decrease the production of L-ascorbic acid (Li et al., 1998), while also down-regulating the expression of *NCS2* genes, including *ZmNCS2–1, ZmNCS2–5*, and *ZmNCS2–15*. The expression levels of two genes were down-regulated by PEG, NaCl, and ABA, as well as low (4°C) and high (42°C) temperatures. Additionally, the expression levels of *ZmNCS2–2, ZmNCS2–3, ZmNCS2–4*, and *ZmNCS2–23* were strongly up-regulated by ABA, suggesting that these genes are important for the ABA stress-related regulatory network. Meanwhile, the expression levels of *ZmNCS2–4* and *ZmNCS2–23* were up-regulated by exposure to 4°C, suggesting that these two genes may help mediate cold tolerance. Environmental stresses frequently threaten maize growth and productivity. Many *NCS2* genes are involved in cellular processes that protect various plant species, including maize and tomato, against environmental stresses (Li et al., 1998; Cai et al., 2014). The subcellular localizations of *ZmNCS2–16*-GFP and *ZmNCS2–23*-GFP in the plasma membrane suggests that *ZmNCS2–16* and *ZmNCS2–23* may be involved in the transport of free nucleobases.

## Conclusion

Our study traced the evolutionary fate of *NCS2* genes in 28 plant species, ranging from single aquatic algae to higher angiosperms, with a major emphasis on *Z. mays* for the first time. Phylogenetic analysis of *NCS2* genes in maize and 27 other species was performed to help better classify and characterize *NCS2* genes in maize and plant kingdoms. As a result, three subfamilies were identified. Members in subfamily I were *NCS2/AzgA-like* genes, while members in subfamilies II and III were *NCS2/NAT* genes. The motif compositional analysis showed one symbolic motif (motif 4) of the *NCS2/NAT* genes was absent in subfamily I *NCS2/AzgA-like* genes. In maize, there were three *ZmNCS2/AzgA-like* and 21 *ZmNCS2/NAT* genes. WGD and single-gene duplication events, but not tandem duplications, were likely important for the maize *NCS2* gene family’s expansion. In addition, *ZmNCS2* genes experienced purifying selection, and those with related evolutionary relationships had similar expression patterns in different tissues or under diverse abiotic stresses, strongly suggesting functional redundancy. Thus, the present study utilized comparative genomics, population genetics and gene expression profiling analysis and provided new insights to better understand the complexity of *NCS2* genes in maize. Their genetic evolutionary relationship and gene expression patterns at diverse developmental stage and under diverse abiotic stress, will benefit further functional analyses of *ZmNCS2* genes.

## Author Contributions

WC and XP conceived and designed this research. WC and WS performed the experiment. WC, BL, YL, and HJ analyzed the data. WC, KZ, ZZ, and JW contributed reagents, materials, and analysis tools. WC wrote the manuscript. All the authors read and approved the manuscript.

## Conflict of Interest Statement

The authors declare that the research was conducted in the absence of any commercial or financial relationships that could be construed as a potential conflict of interest.

## Footnotes

1 www.phytozome.org

2 http://www.Congenie.org

3 http://www.expasy.org/tools/

4 http://www.cbs.dtu.dk/services/TMHMM/

5 https://www.ncbi.nlm.nih.gov/Taxonomy/CommonTree/wwwcmt.cgi

6 https://mafft.cbrc.jp/alignment/server/

7 http://tree.bio.ed.ac.uk/software/figtree/

8 http://meme-suite.org/

9 https://phytozome.jgi.doe.gov/pz/portal.html

10 http://orthomcl.org/orthomcl/

11 http://circos.ca/

12 http://www.bioconductor.org/

13 http://www.csbio.sjtu.edu.cn/bioinf/plant-multi/

14 http://broadinstitute.github.io/picard/

15 https://bioinformatics.psb.ugent.be/plaza/

16 http://www.csbio.sjtu.edu.cn/bioinf/plant-multi/

## Supplementary Material

FIGURE S1 Correlation between the number of *NCS2* genes and the total number of genes in a genome.

FIGURE S2 Analysis of *NCS2* genes from 20 species based on phylogenetic trees constructed using the maximum-likelihood and Bayesian methods. **(A)** Maximum-likelihood tree; **(B)** Bayesian tree.

FIGURE S3 Sliding-window plots of representative duplicated *NCS2* genes in maize. The window and step sizes are 150 and 9 bp, respectively. Nucleotide positions and Ka/Ks ratios are indicated on the *x*-axis and *y*-axis, respectively.

TABLE S1 Gene-specific primers for the qRT-PCR analysis of maize *NCS2* genes

TABLE S2 Protein sequences for the phylogenetic trees constructed using the neighbor-joining, maximum-likelihood and Bayesian methods.

TABLE S3 The genes in different *NCS2* subfamilies.
