# Supplementary figures and images for: Comparative Genomics, Whole-Genome Re-sequencing and Expression Profile Analysis of Nucleobase:Cation Symporter 2 (NCS2) Genes in Maize
Source: Front Plant Sci. 2018 Jun 28;9:856. doi: 10.3389/fpls.2018.00856 (PMC6031955; doi:10.3389/fpls.2018.00856)

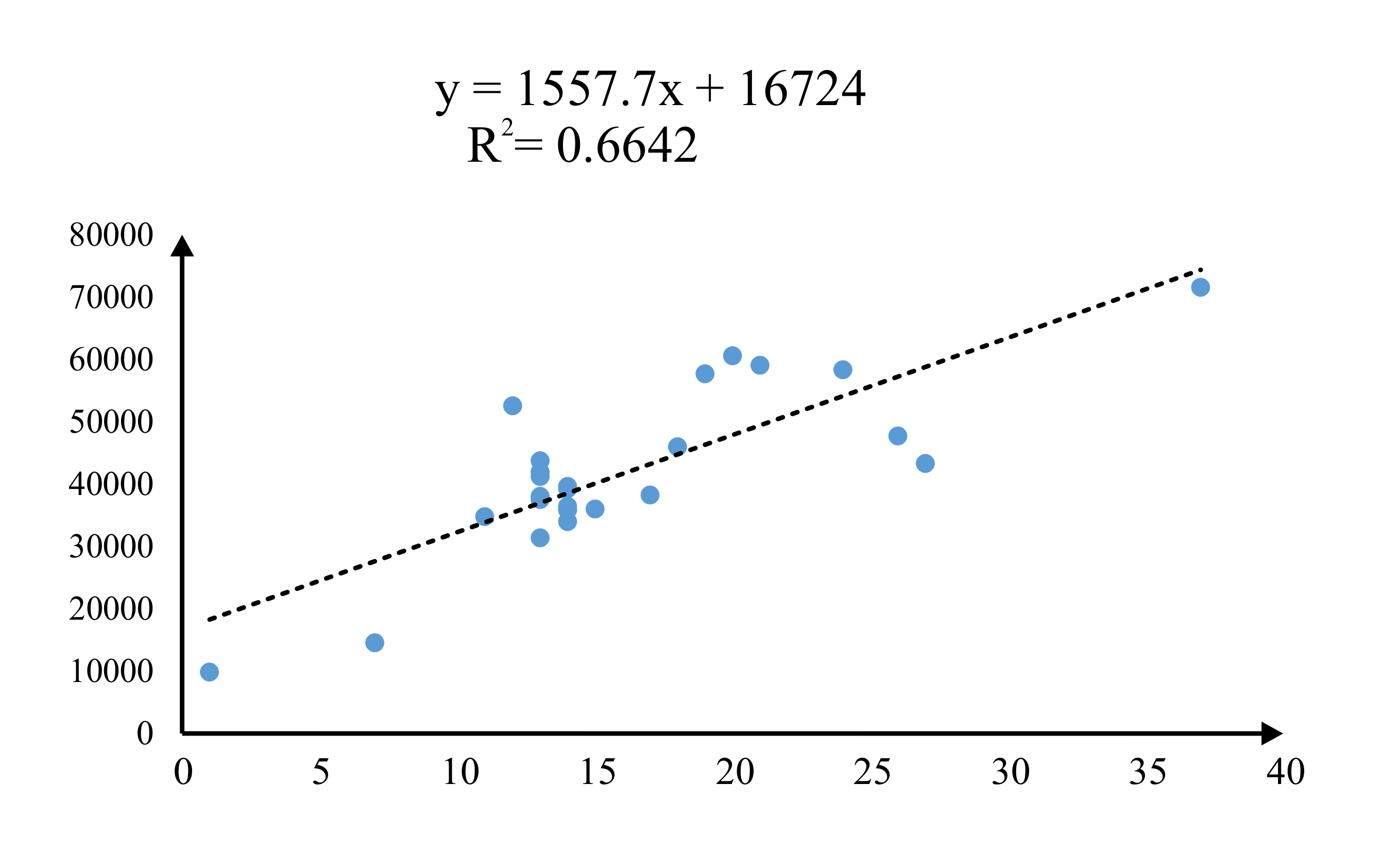

Supplement: FIGURE S1 — Correlation between the number of NCS2 genes and the total number of genes in a genome. [file Image_1.TIF]

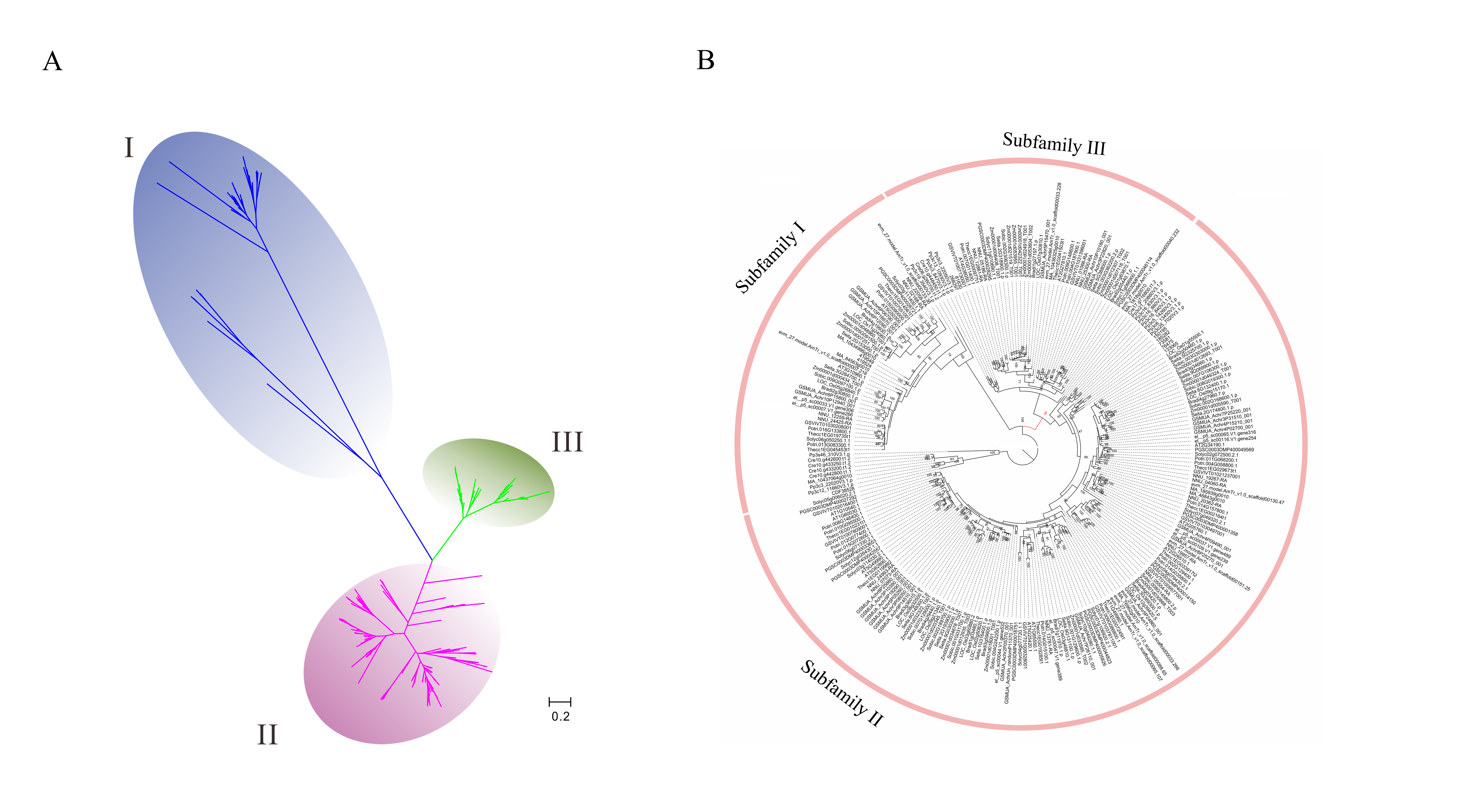

Supplement: FIGURE S2 — Analysis of NCS2 genes from 20 species based on phylogenetic trees constructed using the maximum-likelihood and Bayesian methods. (A) Maximum-likelihood tree; (B) Bayesian tree. [file Image_2.TIF]

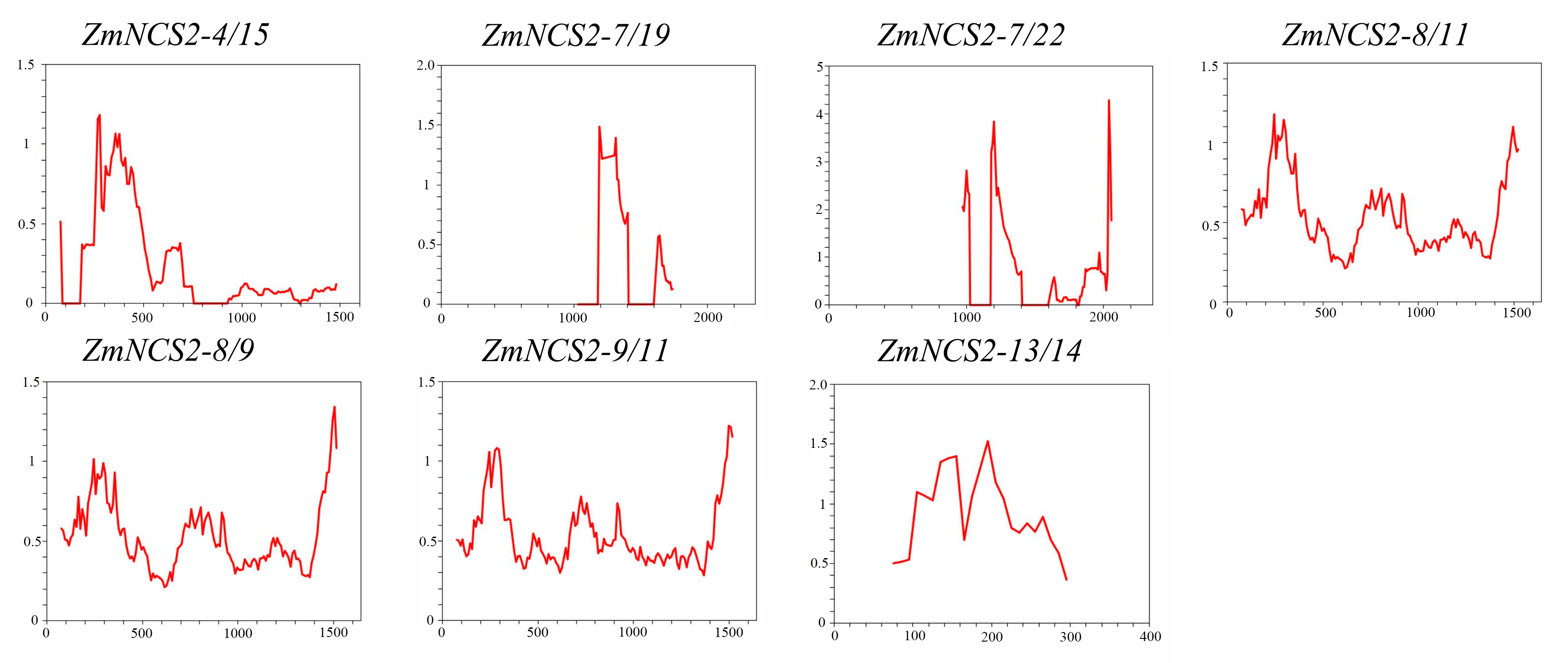

Supplement: FIGURE S3 — Sliding-window plots of representative duplicated NCS2 genes in maize. The window and step sizes are 150 and 9 bp, respectively. Nucleotide positions and Ka/Ks ratios are indicated on the x-axis and y-axis, respectively. [file Image_3.TIF]
