# Supplementary material for: Comparative Genomics, Whole-Genome Re-sequencing and Expression Profile Analysis of Nucleobase:Cation Symporter 2 (NCS2) Genes in Maize
Source: Front Plant Sci. 2018 Jun 28;9:856. doi: 10.3389/fpls.2018.00856 (PMC6031955; doi:10.3389/fpls.2018.00856)
Supplement: TABLE S1 — Gene-specific primers for the qRT-PCR analysis of maize NCS2 genes [file Table_1.DOC]

Table S1. The gene-specific primers of NCS2 in maize used for qRT-PCR analysis.

| ZmNCS2-1 | AGTTATTCAATCGTTCCTCTT |
| --- | --- |
|  | CCTTGTGCTGTTCTCATT |
| ZmNCS2-2 | CTCTTGTTGAGTCATCTG |
|  | AGAACTGAAGGAGGTATC |
| ZmNCS2-3 | GAGAAGTTCAAGTCCTACAAG |
|  | GTACCTGCTCAAGCCATA |
| ZmNCS2-4 | GGAACCACTGTATCTGTC |
|  | TTACCAAGGATTGAGAAGAA |
| ZmNCS2-5 | CGCAATCTCTTCATCATC |
|  | AGAACTTGTGGAATACCTA |
| ZmNCS2-6 | CTTGAGAAAAATATCCCTGTTTGG |
|  | GTCTTAGAATGAAATGTTGGTGGG |
| ZmNCS2-7 | AGGTTGAAGGCATTCCATTAAG |
|  | AATGCCTCTGCTAACAACTCCA |
| ZmNCS2-8 | AATAACCCGGGGTACCAGCAGT |
|  | AAAACGAGGCCCTCCAGCATCA |
| ZmNCS2-9 | TCTGCCTCTCGGGGCGAATGAC |
|  | GAAGGTCACGAACAGGATGCCG |
| ZmNCS2-10 | GTCTTACTACAATACTACA |
|  | TTATCTGGAATACTGAAC |
| ZmNCS2-11 | CTACATCGAGTCCACCGCGGGG |
|  | TGCAGCGCGACGTAGACTCCCA |
| ZmNCS2-12 | TCCTATGCTTGTCTTGTT |
|  | AGAACCTCTCCAATATCG |
| ZmNCS2-13 | AACAAGATGCAGGAGAGTCTCG |
|  | CATCCATCTTCATTTGCCACAT |
| ZmNCS2-14 | CAATGGGTGGTTCTGATGAGGA |
|  | ATAGCCCCCTGTAGTTCCCTCA |
| ZmNCS2-15 | TTCTCAGTACATACCACAT |
|  | GCAATGGACATTATGACA |
| ZmNCS2-16 | TCAAGATGGTTCAACGATA |
|  | ATGCCTGTCAATAGTGTT |
| ZmNCS2-17 | ACCCTCTCGAGAGATTCGTGTT |
|  | GGGAACGCGAAGAAGAAGAGTC |
| ZmNCS2-18 | TGTCTCTTCTTCGCATAT |
|  | TGATGAACTTGGTCCTAA |
| ZmNCS2-19 | TAACTTGTGGCAGTACCTCCGG |
|  | AGGAAAAAACTGGAGTTCCCCA |
| ZmNCS2-20 | GGAGCATATAACTTCAAAGGCT |
|  | AACTGAAGCAACCAGAGAAACT |
| ZmNCS2-21 | GTCTTACTACAATACTACAT |
|  | ATTATCTGGAATACTGAAC |
| ZmNCS2-22 | TCCTCTCGTGATGGTCCCTGCT |
|  | TGTAGTCACCCCGGTGACGAGC |
| ZmNCS2-23 | TGGAGCCTATAAGAACAG |
|  | GCAGAAGAGATGAGATTG |
| ZmNCS2-24 | TCTCCTTGTCGATACCCGCATA |
|  | ATGTCTACTGCCTGGCACTGTG |
