# Supplementary material for: Comparative Genomics, Whole-Genome Re-sequencing and Expression Profile Analysis of Nucleobase:Cation Symporter 2 (NCS2) Genes in Maize
Source: Front Plant Sci. 2018 Jun 28;9:856. doi: 10.3389/fpls.2018.00856 (PMC6031955; doi:10.3389/fpls.2018.00856)
Supplement: TABLE S2 — Protein sequences for the phylogenetic trees constructed using the neighbor-joining, maximum-likelihood and Bayesian methods. [file Table_2.DOC]

Table S2 The sequences of genes used in phylogenetic analysis.

>ZmNCS2-9

MALSIIPSTSSGEHAAASASATATTTTKLGQLNAAVERSWVGRRFRLAARGTTFTTELRAGTATFLTMAYILAVNASILSDSGATCTVDDCDVPSPGCKFPPVDPGYAACVARVRRDLIVATAASSVIGSFIMGAFANLPIALAPGMGTNAYFAYTVVGFHGSGTLPYRTALAAVFLEGLIFLFISIVGLRSKLAQFIPTPVRISASAGIGLFLAFIGLQSNEGVGLVGFSSSTLVTLGACPASQRASVAPVLTFPNGTVALMPGGTVSGGILCLSGRMTSPTFWLAVVGFLIIAFCLIKRVKGALIYGILFVTFVSWPRHTAVTAFPDTPAGDDSFHYFKKVFDVHRIRSTAGALDFSGIGQGYFWEALFTFLYVDILDTTGSLYTMARFAGFVDDATGEFEGQYFAFMSDATAIVFGSLLGTSPVTAFIESSTGIREGGRTGLTALTAAIYFAAALFITPVLASIPSWAVGPPLVLVGVMMMRAVAEVDWDDMRQAVPAFLTLALMPLTYSIAYGLIGGIASYMLLHSWDWACQATRRLGCLKKGGGGADSTNGGVAEQSVEEQRKDMESA------------------------------------------------------------------------------------------------------------------------------------------------------------------------------------------------------------------------------

>ZmNCS2-5

MESLTLRASTAASKLQRRRLLPGRQRATYVRATASAGEPDLSVRVNGLQMQWGRGGTDFRSLRTCSSYADVQQAVVAVCVWLMKSDLGTMDVLFTLLSLVPGQILCGYPTLLAVATSPRSSSPLLCSQGRFLPLPRLIVLEKPSLSTRFRSWTWTLSSATQSSRTRPALVPADSEMAEVKPEDMVHHLPMDQLQGLEYCIDSNPSWGEGIALGFQHYILSLGTAVMIPTLLVPLMGGNDDDKAKVVQTLLFVTGIKTLLQTLFGTRLPTVMGGSYAYVVPILSIVRDPSFARIADGHTRFLQTMRAVQGSLIVSSSIQIILGYSQLWAICSRFFSPLGMVPVVALVGLGLFERGFPVVGKCVEIGLPMLILFVALSQYLKHVHIRHVPVFERFSLLMCITLVWVYAHILTASGAYKHTALVTQINCRTDRANLISSSQWISIPYPLQWGAPTFSADHAFGMMAAVMVSLIETTGAFKAAARLASATPPPAYVLSRGIGWQGIGTLLDGLFGTGTGSTVSVENVGLLGSTRVGSRRVIQISAGFMIFFSILGKFGALFASIPFTIFAAIYCVMFGIVAAVGLSFMQFTNMNSMRNLFIIGVSLFLGLSIPEYFSRYSTSSQQGPAHTKAGWFNDYINTSFSSPPAVALIVAVLLDNTLDVRDAARDRGMPWWARFRTFRGDSRNEEFYTLPFNLNRFFPPT-----------------------------------------------------------------------------------------------

>ZmNCS2-6

MPSSRRTTGRGGAGAGAGGAGAGDAGGGDGDRVPRFMGNNRDHNPRELRSWARRTGFHPSAFFSGESNSSFASSAAPQPPPPPPPASSRRPPRPPPERDPDPDAEDDLDPAPGRGRGRRPRRRIDLRGELQIPPGFGREEAVPGSAEPDARRGGARGDATRRNGGVERGQAPANAGRNGNGALADADARKKAEDAEAKRKAEEAEARRKKEDEERDAELAAYYQEQWANEDEGAAGAAAAAAETAPLYEASGLRCGVTENPGWAPLIFYGIQHYLSIAGSLVFVPLILVPTMGGSDKFKHIMRELQGAILVGSVFQIILGYTGLMSLFLRLINPVVVAPTIAAVGLAFFSYGFPQAGSCVEISMPLILLVLLCTLYLRKISLFGNHIFLVYAVPLSVAIVWAYSFFLTAGGAYNFKGCSSNIPSSNILLDSCRRHLETMRRCRTDVSSAWRTAAWVRIPYPFQWGPPTFHSKTGIIMIIVSLVASVDSLSSYHAASLLVNLSPPTRGVVSRAIGLEGISSFIAGVWGTGTGSITLTENIHTLETTKMASRRALQLGAAVLVVCSFFGKIGALLASIPLALAASVLCFTWALIVALGLSTLRYTQAASSRNLIIVGFTLFISLSIPAYFQQYEPSSNLILPSYLLPYAAASSGPVRTASSGLNYAVNALLSINVVVALLVALILDNTVPGSRQERGVYVWTDPKSLEVDPATLEPYRLPEKISCWFRWAKCVGM--------------------------------------------------------------

>ZmNCS2-7

MAAGAHCVVQPSAKGMEYTWSSHGPTADGDFGVSISAPGGAVAPVPTWTLQSRMLMNGTSMSSPSACGGVALLVSGMKVEGIPLSPYSVPSIIENTAASISNAPEEKLTTGNGLLQVDRAFEYAQQANKLPLVSYRISINQVGSQVGSYHASSLFVATRPPTYGVVSRGIGVEGVSTVLAGLWGTGVGSATITENVHTIAVTKMGSRRAVGFGAILLVLLSIVVKNSHAVRKGKVGAFIASIPDVMVAALLCFMWAMLCALGLSNLHYSATGSSRNSIIVGLALFLSLSVPSYFQQYGVHPSANSSVPTYFQPYVVASHGPVHIGSGGVNYVLNTILSFNMAIAFLVALVLDNTVPGGRQERGLYVWSEAEATMRESTFMKDYELPFKIGRLFRVWSCLGQDDMATVVSTVLLITEVTTLLHMFVGTRLPLVQGPSFVYLAPALAIINSQEFFRINDNVIE----------------------------------------------------------------------------------------------------------------------------------------------------------------------------------------------------------------------------------------------------------------------------------------------------------------------------------------------

>ZmNCS2-8

MTSKGTRPWSRLSEAETAVNRAVAGSRVGRYFKLDARKSSFTKELRAGAATFLTMAYIISVNAAVLTDSGGPCTVRDCTPVPTNSTVAATPPGPECTVAGANNPGYQQCLARTKSDLVVATAVAAMAGSFAMGLFANLPLALAPGMGANAYFAYNMVGFHGSGPIGYSTALAVVMLEGLVFFALSVVGLRSRLARMIPRNIRLASAVGIGLFLAFTGLQAHQGVGLVGASPSTLVTLTACSDVDTVTGACLGGTMRSPTFWLGAVGFLITATCLARDVKGSMIFGMLFVTVVSWIRGTSVTMFPDTPVGNAGFAYFKKVVDFHMIRSTAGQLSFGGFRHGNVWLALLTLLYVDVLDTTGTMYSMAEYGGFTDGAGGGFEGEYRAFLVDAGSTVLSAGLGSSTVTTYIESTAGIREGGRTGLTAITVAACFLASLFFGPLLMSVPPWAVGPSLVLVGAMMMRVAKDIEWGDMKEGVPAFVTMALMPLSFSIANGIIAGLGVYVALHWYDWARHGYGKVRNALDERRNQVAAAAGEVGPAAQDVV------------------------------------------------------------------------------------------------------------------------------------------------------------------------------------------------------------------------------------------------------------

>ZmNCS2-22

MSSTSGALPQPRRGRPGPWPPAPPPQPQAQPLSWAKRTGFQSRVSGESLPSSSAHNSGQAPLPRPAEAPSDLESGPPARPSSTLPAPPAAAGNGERQHPPPPPPQARTRRRDSDSGRPNGQIAAPSLPQLQEEEEAPERPAHVKYELRDTPGIFPLVVYGFQHYISMVGSIILIPLVMVPAMGGSAVSSGIEFVVSRYIWSCLGQDDMAAVVSTVLLVTGVTTLLHMFVGTRLPLVQGPSFVYLAPALAIINSPELFGINDNNFKHIMKHLQGAIIIGGAFQVFLGYTGLMSLFLRLINPVVVSPTVAAVGLSFFSYGFTKIGTCIEMGILQLLMVVIFALYLRKIKLFGYRVFLIYAVPLGLGITWAVAFVLTATGVYSYKGCDANIPASNNVSAFCRKHVLRMRSCRVDTSHALRSSPWFRFPYPLQWGTPVFSWKMGLVMCVVSVIASVDSVGSYHASSLFVATRPPTSGVVSRGIGVEGVSTVLAGLWGTGVGSATITENVHTIAVTKMGSRRAVGFGAILLILLSIVGKVGAFIASIPDVMVAALLCFMWAMLCALGLSNLRYSATGSSRNSIIVGLALFLSLSVPSYFQQYGVHPSANSSVPTYFQPYVVASHGPVHTGSGGVNYVLNTILSLNMAIAFLVALVLDNTVPGGRQERGLYVWSEAEAAMRESTFMKDYELPFKIGRPFRWVKCVGL----------------------------------------------------------------------------------------------

>ZmNCS2-23

MAEVKPEEISHPPMEQLQGFEYCIDSNPPWGEAIILGFQHYILALGTAVMIPTVLVPMMGGDDGDRVRVVQTLLFVTGINTLLQSLFGTRLPTVIGGSYAFVIPIMAIIQDPSLSGIPDGHERFLETMKAIQGALIVSSSIQIILGYSQLWGIFSRFFSPVGMTPVVALLGFGLFERGFPVVGRCVEIGLPMLILFVVLSQYLKNIQIKEIPILERFSLFICVALVWAYAQILTSGGAYKNSAEVTQNNCRTDRANLISSAPWIKIPYPLQWGAPTFNAGQSFGIVSAVLVSLVESTASYKAAARLASATPPPAHILSRGIGWQGIGILLDGLFGTGTGSTVSVENVGLLGSTRIGSRRVIQISAGFMIFFSMLGEQNMSLLRAKGGGRLLTPLHFILSVAGKFGALFASIPFTIFAAVYCVLFGLVAAVGLSFLQFTNMNSMRNLFIVGVSIFLGLSVPEYFFRYTMAAHRGPAHTKAGWFNDYINTIFSSPPTVGLMVAVFLDNTLEVKQAGMDRGMPWWQRFRTFKGDSRNEEFYRLPFNLNRFFPPA----------------------------------------------------------------------------------------------------------------------------------------------------------------------------------------------------------------------------------------------------

>ZmNCS2-15

MAAPAPAPKQEELQPHAVRDQLPSVSYCLTSPPPWPEAVLLGFQHYLVMLGTTVIIPTALVPQMGGGNEEKARVVQTLLFVAGINTLVQSFLGTRLPAVMGASYTFVAPTISIVLAGRYSGIADPHEKFVRIMRGTQGAFIVASTLQIIMGFSGLWRIVVRLLSPLSAAPLVALVGFGLYELGFPSVAKCVEIGLPQILLLVALSQYIPHAAPLLSTAFERFAVIMSIALIWLYAFFLTVGGAYKNAAPKTQFHCRTDRSGLVGGAPWISVPYPFQWGAPTFDAGEAFAMMAASFVALVESTGAFIAVSRYASATPCPPSVMSRGIGWQGVGILLGGIFGTANGTSVSVENAGLLGLTRVGSRRVVQISAGFMIFFSILGKFGAVFASIPGPIIAAIYCLLFAYVGTAGVGFLQFCNLNSFRTKFILGFSLFMGLSVPQYFNEYTSVAGFGPVHTRARWAFVGGAVAYFLDNTLQRRDGAVRKDRGHHFWDRFRSFKTDPRSEEFYSLPFNLNKFFPSF------------------------------------------------------------------------------------------------------------------------------------------------------------------------------------------------------------------------------------------------------------------------------------

>ZmNCS2-16

MAGGGGGGAAPKHDDFTPHPVKDQLPGVSYCITSPPPWPEAVLLGFQHYLVMLGTTVIIPTALVPQMGGNNEDKAVVIQTLLFVAGINTLLQSFFGTMLPAVIGGSYTFVLPTISIILAGRYANEPNPHIKFLRIMRGTQGALIVASALQIIVGFSGLWRNVARYLSPLSAAPLVALVGFGLYELGFPSVAKCVEIGLPQLILLVIFTMYLPHAVHMLKSIFDRFAVLFTIPIVWLYAYLLTVGGAYRNAPPKTQFHCRTDRSGLIGGAPWIRVPYPFQWGAPTFDAGEAFAMMAASFVALVESTGAFIAVSRYASATPIPPSVLSRGIGWQGIGILLDGLFGTGNGSSVSVENAGLLALTRVGSRRVVQISAGFMIFFSILGKFGAVFASIPAPIFAAMYCIFFAYAGSAGIGFLQFCNLNTFRTKFILGFSVFMGLSVPQYFNEYTSIAGYGPVHTHSRWFNDIVNVIFSSKAFVAGFVAYLLDNTIDRHEASVRRDRGYHWWDKFRSYRTDTRSEEFYSLPFNLNKFFPSV---------------------------------------------------------------------------------------------------------------------------------------------------------------------------------------------------------------------------------------------------------------------

>ZmNCS2-18

MAAAPPPKADELQPFPPKEQLPGVAFCITSPPPWPEAILLGFQHFVVMLGTTVIIPSALVPQMGGGNEEKARVVQTILFVAGINTLFQTLFGTRLPVVMGGSYVFVGPTISIVLAGRYSNEADPHEKFLRTMRGTQGALLVASTIQIILGFSGLWRNVVKLLSPLAAVPLVSLVGFGLYELGFPGVAKCVEVGLPELLLLVVFSQYLPQVLDFGKSVFSRFSVLFTVAIVWLYAYILTIGGAYKNSPPKTQVHCRVDRSGLISGAPWISVPYPFQWGAPTFDAGEAFAMMMTSFIALVESTGAFIGASRYASATMIPPSIISRGVGWQGIGLLLDSFFGTANGTSVSVENIGLLALTRIGSRRVVQISAGFMIFFSVLGKFGALFASIPLPVFAGMYCLFFAYVGGVGLSLLQFCNLNSFRTKFIMGFAFFMGLSVPQYFNEYTAVASYGPVHTGARWFNDMINVPFTSKPFVAGLVAYILDNTLQVKESAVRKDRGNHWWEKFRSFKKDARSQEFYSLPFNLNKFFPSV-------------------------------------------------------------------------------------------------------------------------------------------------------------------------------------------------------------------------------------------------------------------------

>ZmNCS2-19

MQFHLFFVLFLTIYLFNLWQYLRKIKLFGYKVFLIYAVPLGLGITWVVAFVLTATGVYSYKGCDANIPASNNVSAFCQKHWGTPVFSWKMGLVMCVVSVIASVDSVGSYHASSLFVATRPPTSGVVSRGIGVEGVSTVLAGLWGTGVGSATITENVHTIVVTKMGSRRAVGFSAILLVLLSIVGKVDAFIASIHDVMVAALLCFMWAMLCALGLSNLRYRATGSSRNSIIVGLALFLSLSVPSYFQ---------------------------------------------------------------------------------------------------------------------------------------------------------------------------------------------------------------------------------------------------------------------------------------------------------------------------------------------------------------------------------------------------------------------------------------------------------------------------------------------------------------------------------------------------------------------

>ZmNCS2-20

MPSSRRTTGRGGAGGGSAGAGDAGGGDGDRVPPFMGNNRDHNPRELRSWARRTGFHPSAFFSGESNSSFASSAAPQAPPPPPPPPVSSRRPPPAREREPEPDTEDDLVPAPPLELERGGPAPVRGRGGRPRRRIDLRGELVIPPGFGREEAEPDAGKGRARGDARRRNFGVEMDQTPANAGRNGNGALADAESRKKSEEAEADAKRKAEEAEARRKKEEEERDAELAAYYQEQWANEDEGAADAAAAETAPLYEASGLRCGVTDNPGWALLIFYGMQHYLSIAGSLVFGPLILVPTMGGSDEDTATVISTMLLVSGLTTILHTFLGSRLPLIQGSSFVYLAPALVIANSEEFRNLSDNKFKHIMRELQGAILVGSVFQIILGYTGLMSLFLRLINPVVVAPTIAAVGLAFFSYGFPQAGSCVEISMPLILLVLLCTLYMRKISLFGNHIFLVYAVPLSVAIVWAYAFFLTAGGAYNFKGCSSNIPSSNILLDSCRRHLETMRRCRTDVSTAWKTAAWVRVPYPFQWGPPTFHFKTVIIMIIVSLVASVDSLSSYHAASLLVNLSPPTRGVVSRAIGLEGVSTFIAGVWGTGTGSTTLTENIHTLETTKMGSRRALQLGAAVLVIFSFFGKIGALLASIPLALAASVLCFTWALIVALGLSTLRYTQAASSRNMIIVGFTLFISLSIPAYFQQYEPSSNLILPSYLLPYAAASSGPVHTASSGLNYAVNALLSINVVVALLVALILDNTVPGSKQERGVYIWTDPKSLEVDPATLEPYRLPEKVSCWFRWAKCVGI

>ZmNCS2-21

MQHYLPIAGSLVFVPLILVPAMDGSDEDTATVISTMLLVSGLTTILHTFLGSRLPLIQGSSFVYLAPALVIANSEEFRNLSDNKFKHIMRELQGAILVGSVFQIILGYTGLISLFLRLINPVVVAPTIAVVGLAFFSYGFPQAGSCVEISMPLILLVLLCTLYMRKISLFGNHIFLVYAVPLSVAIVWAYAFFLIAGGAYNFKCCSSNIPSSNILLDSCRRHLETMRRCRTDVSTAWKTTAWVRV----------------------------------------------------------------------------------------------------------------------------------------------------------------------------------------------------------------------------------------------------------------------------------------------------------------------------------------------------------------------------------------------------------------------------------------------------------------------------------------------------------------------------------------------------------------------

>ZmNCS2-24

MQHYLSIAGSLVFVPLILVPAMGGSDEDTTTVISTMLLVSGLTTILHTFLGSRLPLIQGSSFVYLAPALVIANSEEFRNLSDNKFKHIMRELQGAILVGSVFQIILGYTGLISLFLSADFLKLSSLPVGKIGALLASIPLALAASVLCFTWALIVALGMSILRYTQAASSRNMIIVGFTLFISLSIPAYFQQYEPSSNLILPSYLLSYAAASSGPVRTASSGLNYAVNALLSIDVVVALLVALILDNTVPGSRHLDRSQIPRGGSCDIGTLPIAGESFMLV----------------------------------------------------------------------------------------------------------------------------------------------------------------------------------------------------------------------------------------------------------------------------------------------------------------------------------------------------------------------------------------------------------------------------------------------------------------------------------------------------------------------------------

>ZmNCS2-1

MGETNELPPPPVAAPPPMMPMQSMMVGAGAIHPPHEQFHHLNYCVHSNPSWVQVAALAFLHYLVMLGSTVMLATAIVPAMGGNAGDKARVIQSFLFMSGINTLLQTLIGTRLPTVMNASFAFVVPVLSIAKEIESNNFLNDHERFKHTMRTAQGALIVASILNMILGFSTIWGAYAKKFSPVIMTPVVCVVGLGLFQLGFPQVGKCVEIGLPMLILAVVVQQYVPNYFSYIHQRVTFLFERYSLLLCIGIVWAFAAILTAAGAYDHASPKTQQHCRTDKSFLMSSAPWIKIPLPFEWGPPIFTAGHSFGMMGAVLVAAFESTGAHFATARLAGATPPPAYVLSRSVGLQGIGMFLEGIFSVPAGSSVSVENIGLLGLTKVGSRRVIQISTGFMIFFSIFGKFGAFFASIPLPIFAAIYCILFGIVAAVGVSFMQFANKNSMRNIYIIGLSLFLGISVPQYFNGYTSSAGGHGPARTNAGWFNDIINTVFASGPTVALIVASVLDNTLEFRGYEADRGLSWFQPFLRRHKGYSDPRNEEFYSFPISVYDVIPDRFRR-----------------------------------------------------------------------------------------------------------------------------------------------------------------------------------------------------------------------------------------------

>ZmNCS2-2

MAGGGAAPPPKQEELQPHPVKDQLPSVSYCITSPPPWPEAVILGFQHYIVMLGTSVIIPSALVPQMGGGNEEKARVIQTLLFVAGINTLCQSFFGTRLPAVMGGSYTIVAPTISIIMAGRYSNEADPREVVEFSEKFLRTMRGTQGALIIASTIQIVLGFSGLWRNVVKLLSPLSAVPLVSLAGFGLYELGFPGVAKCVEIGLPEIILMLIFSQYLPHAVHAAKPVFDRFSVIFTIAIVWLYAYILTVSGAYKSARTKTQLHCRVDRSGLVGGAPWISVPYPFQWGAPTFDAGESFAMMVAAFVALVESSGAFIAVSRYASATMIPPSVLGRGIGWQGIGTLLGAFFGTANGTAVSVENAGLLALTHVGSRRVVQISAGFMIFFSVLGKFGAIFASIPLPIFAALYCILFAYIGACGLSFLQFCNLNSFRTKFIMGFSLFMGLSVPQYFNEYTSVAGYGPVHTGARWFNDMINVPFSSKPFVAVLVAFLLDNTIQVRDSGVRRDRGYHWWDKFRSFKTDSRSEEFYSLPFNLNKFFPSV----------------------------------------------------------------------------------------------------------------------------------------------------------------------------------------------------------------------------------------------------------------

>ZmNCS2-3

MPPVKAEDLVVHAVKEQFAGLDYCITSPPPWITTVLVGFQHYLVMLGTTVLIATIIVPLMGGGHAEKAIVIQTILFLSGINTLLQVHFGTRLPAVMSGSYTYIYPAVAIILSPRYALLIDPLELIHRWLVSSVQRFVFTMRSLQGALIIAGVFQAVVGFFGIWRVFIRFLSPLAAVPFVTLTGLGLFFFAFPGVTKCIEVGLPALVLLVIFAEYASHVFAKGSFVFSRCAVLVTVVIIWIYAEILTAAGAYNERGPVTQFSCRADRSGIIQGSPWVRFPYPFQWGYPIFCFQDCFAMLAASFASLIESTGTLIAVSRYSGATFCPPSVFSRGIGWEGISIILDGMCGTLTGTAASVFEVTCSLCCFQSENAGLLAVTRVGSRRVIKISALFMIFFSLFAKFGAVLASIPLPIFAALYCVLFAYSAGAGFSLLQYCNLNSLRTKFILSISLFLGLSIPQYFRVYEMFFGFGPVHTHSVAFNVMVNVIFSSPATVAAILAYLLDCTHLYWEASVKKDRGWFWWEKFKSYKYDGRSEEFYRLPYGLSRYFPSL-----------------------------------------------------------------------------------------------------------------------------------------------------------------------------------------------------------------------------------------------------

>ZmNCS2-4

MAAPAPKQEELQPHAVRDQLPSVSYCLTSPPPWPEAVLLGFQHYLVMLGTTVIIPTALVPQMGGGNEEKARVVQTLLFVAGINTLIQSFLGTRLPAVMGGSYTFVAPTISIILAGRYSGIADPHEARLAVLPASSSKFRLSGLPTNLFAGCRNSCASCGARKARSLWPPPSKSSLASVAFGALSSGSSPALGPPRVFRSRCFLSSSTTPSASANSCCRLLSPLSAAPLVALVGFGLYELGFPSVAKCVEIGLPQILLLVALSQYVPHLVPLLSTAFERFAVIMSVVLIWLYAFFLTVGGAYKNAAPKTQFHCRTDRSGLVGGAPWISVPYPFQWGAPTFDAGEAFAMMAASFVALVESTGAFIAVSRYASATPCPPSVMSRGIGWQGVGILLGGLFGTANGTTVSVENAGLLALTRVGSRRVVQISAGFMIFFSILGKFGAVFASIPGPVIAAVYCLLFAYVGMAGVGFLQFCNLNSFRTKFILGFSLFMGLSVPQYFNEYTSVAGFGPVHTRARWFNDMINVVFSSKALVGGAVAYFLDNTLHRRDGVARKDRGHHFWDRFRSFKTDPRSEEFYSLPFNLNKFFPSF---------------------------------------------------------------------------------------------------------------------------------------------------------------------------------------------------------------

>ZmNCS2-17

MPPVKAEDLVVHAVKEQFAGLDYCITSPPPPWITTVLVGFQHYLVMLGTTVLIATIIVHFGTRLPAVMSGSYTYIYPVVAIILSQRYALLIDPLERFVFTMRSLQGVLIIVGVFQAVVGFFGIWRVFIRFLSPLTAVPFVTLTGLGLFFSAFPGVTKCIEVGLPALVLLVIFAEYASHVFAKGSFVFSRCPVLVTVVIIWIYAEILTAAGAYNQLGIT-------------------------------------------------------------------------------------------------------------------------------------------------------------------------------------------------------------------------------------------------------------------------------------------------------------------------------------------------------------------------------------------------------------------------------------------------------------------------------------------------------------------------------------------------------------------------------------------------

>ZmNCS2-10

MLLVSGLTTILHTFLGSRLPLIQGSSFVYLAPALVIANSEEFRNLTDNKFKHIMRELQGAILVGSVFQIILGYTGLISLFLSADFLKLSSLPVGKIGALLASIPLALAASVLCFTWALIVALGMSTLRYTQAASSRNMIIVGFTLFISLSIPAYFQQYEPSSNLILPSYLLSYAAASSGPVHTASSGLNYALNALLSIDVVVALLVALILDNTVPGSRQERGVYI------------------------------------------------------------------------------------------------------------------------------------------------------------------------------------------------------------------------------------------------------------------------------------------------------------------------------------------------------------------------------------------------------------------------------------------------------------------------------------------------------------------------------------------------------------------------------------------

>ZmNCS2-11

MAATSSKATGPWSRLSEAEAAVNRAVAGSHVGRYFKLDARKSSFTKELRAGAATFLTMAYIISVNAAILTDSGGPCTARDCTPVATATNSTTADCAVVGAKANPGYQQCLARTKSDLIVATAVAAMAGSFAMGLFANLPLALAPGMGANAYFAYNMVGFHGSGPIGYRTALAVVMLEGLVFFALSAVGLRSRLARMIPRNIRLASAVGIGLFLAFTGLQAHQGVGLVGASPSTLVTLAACSDVDPATGACLGGTMRSPTFWLGAVGFLITATCLGRDVKGSMIYGILFVTVVSWIRGTSVTVFPDTAAGNAGFDYFRKVVDFHVIRSTAGQLSFGGFRHGNVWLALLTLLYVDVLDTTGTMYSMAEYGGFVKEGGGFEGEYRAFLVDAGSTVLSAGLGSSTVTTYIESTAGIREGGRTGLTAITVAACFLASLFFGPLLMSVPPWAVGPSLVLVGAMMMRVAREIEWGDMKEGVPAFVTMALMPLSFSIANGIIAGLGVYVALHWYDWARHGYGKLRNALDERRNQVAAAAATATAGEVGPAAQGIV--------------------------------------------------------------------------------------------------------------------------------------------------------------------------------------------------------------------------------------------------------

>ZmNCS2-12

MEMTDMKPEEMSHPPMDQLLGMEYCIDSNPSWGGAVLLGFQHFILCLGTAVMIPTLLVPLMGGNAHDKAKVVQTVLFVTGINTMLQTLFGTRLPTVIGGSYAFLVPVMSVISDHSLIQIADNHTRFKMTMRAIQGALIISSCIQIILGFSQLWGVCSRFFSPLGMVPVIALVGLGLFERGFPVIGTCVEIGVPMLVLFVALSQYLKHVQVHPFPILERFSVLITIAVVWLYAHILTVSGAYKHSSQVTQLNCRTDRASLITTMPWFDIPYPLQWGPPSFSADHSFGMMAAVLVSLVESTGAFKAAARLASATPPPPFVLSRGIGWQGIGLLLDGLFGTASGSTVSVENVGLLGSTRIGSRRVIQISAGFMIFFSILGKFGGLFASIPFTVFAAIYCVLFGYVGAVGLSFMQFTNMNSMRNLFIIGTSLFLGISIPEYFFHYDHGPSHTRAGWFNDLINTIFSSPPTVGFIISVVLDNTLDVRNRAKDRGMPWWARFRTFRGDSRNEEFYNLPFNLNRFFPPS---------------------------------------------------------------------------------------------------------------------------------------------------------------------------------------------------------------------------------------------------------------------------------

>ZmNCS2-13

MQKFKHIMRELQGAILVGSVFQIILGYTGLISLFMRLINPVVVALTIAAVGLAFFSYGFPQAGSCVEISMPFILLVLLCTLYMRKISLFGNHIFLVYALADYTGMRYFSNPLPLHVEFADFLKLSSLPVGKIGALLASIPLALAASVLCFTWALIVALGLSTLRYTQAASSRNLIIVGFTLFISLDAYNKDL---------------------------------------------------------------------------------------------------------------------------------------------------------------------------------------------------------------------------------------------------------------------------------------------------------------------------------------------------------------------------------------------------------------------------------------------------------------------------------------------------------------------------------------------------------------------------------------------------------------------------

>ZmNCS2-14

MQHYLSIAGSLVFVPLILVPAMGGSDEDTATVISTMLLVSGLTTILHTFLGSRLPLIQGSSFVYLAPALVIANSEEFRNLSDNCIQDPIWGLFEKGRSIGAKSLGGFLGQSLLFVYQKFKHIMRELQGAILVGSVFQIILGYTGLMSLFLRLINPVVVAPTIAAVGLAFFSYGFPQAGSCVEISMPLILLVLLCTLVPLSVAIVWAYAFFLTAGGAYNFKGCRSNIPSSNILLDSCRRHLETMRRCRTDVSTAWKTAAWVRVPYPFQWGPPTFHFKTGIIMIIVSLVAPVDSLSSYHAASLLLNYAVNVLLSINVVVALLVALILDNTVPGSRQERGVYIWTDPKSLEVDPATLEPYRLPEKVSCWLRWAKCDKVLLFLTQH-----------------------------------------------------------------------------------------------------------------------------------------------------------------------------------------------------------------------------------------------------------------------------------------------------------------------------------------------------------------------------------------------------------------------------

>AT1G10540.1

MLGTTVLIPTMLVSKIDAKLIQTLLFVSGINTLFQSFFGTRLPAVIGASYSYVPTTMSI

VLARFEQIMRGIQGALIIASFLHILVGLSPLSAVPLVAFSGFGLIGLPEIILLVIFSFHR

FAVIFSVVIVWLYAYILTIGGAYSCRTDRAGIISASPESTGTYIAVSRYASATPIPPSVL

SRGQGFGILLCGLFGAGNATSENAGLLAVTRVGSRRVIQVAAGFMIFFSILGKFGAIFA

SIPAPIVAALYCLIQFCNLNSFRTKFILGFSIFMGLS

>AT1G60030.1

MLGTTVLIPTYLVPQMGGKMVQTLLFVSGLNTLLQSFFGTRLPAVIGGSYTYVPTTLSI

ILAKFKRIMRGIQGALIVASILQIVVGLSPLSAVPLVALAGFGLIGLPEIILLLLFSFHR

FAVIFSVVIVWIYAHLLTVGGAYKCRTDRSGLISGSPESTGTYIVVSRFASATPPPPSVL

SRGQGVGVLLCGLFGAGNGASENAGLLALTRVGSRRVVQISAGFMIFFSILGKFGAIFA

SIPAPVVAALHCLLQFCNLNSFRTKFILGFSVFMGLS

>AT5G49990.1

MLGTTVLIPSALVPQMGGKLIQTILFVAGLNTLLQTVFGTRLPAVIGASYTFVPVTISI

MLSRFKRIIRATQGALIVASTLQIILGLSPLSAAPLVGLVGYGLIGLPGLIILILISFAR

FAVIFSVAIVWLYAFFLTLGGAYNCRTDRAGLISAAPESTGAFIAVSRYASATMPPPSVI

SRGQGVAILISGLFGTGIGSSENAGLLALTKIGSRRVVQISAGFMIFFSILGKFGAVFA

SIPSPIIAALYCLLQFCNLNSFRTLFILGFSIFLGLS

>AT5G62890.1

MLGTTVLIPTALVPQMGGKVIQTILFVAGINTLLQTLFGTRLPAVVGASYTFVPTTISI

ILSRFERIMRATQGALIVASTLQMILGLSPISAVPLVGLVGFGLIGLPELLILVFVSFDR

FAVIFAVVIVWIYAHLLTVGGAYNCRTDRAGIIGAAPESTGAFVAVSRYASATMLPPSIL

SRGQGVAILISGLFGTGAGSSENAGLLALTRVGSRRVVQIAAGFMIFFSILGKFGAVFA

SIPAPIIAALYCLLQFCNLNSFRTKFILGFSVFLGLS

>Potri.012G077400.1

MLGTTVLIPSALVPQMGGEVIQTLLFVAGLNTLLQSLFGTRLPAVIGGSYTFVPTTISI

ILAKFKRIMRATQGALIVASTLQIVLGLSPLSAVPLVALVGFGLIGLPELIILVFISFDR

FAVIFAVVIVWIYAHLLTVGGAYNCRTDRAGLIDGSPESTGAFIAVSRYASATPMPPSVL

SRGQGVAILLSGLFGTGNGSSENAGLLALTRVGSRRVVQISAGFSVLGKFGAVFA

SIPSPIIAGLYCLLQFCNLNSFRTKFILGFSIFMGLS

>Potri.015G072600.1

MLGTTVLIPSALVPQMGGDVIQTLLFVAGLNTLLQSLFGTRLPAVIGGSYTFVPTTISI

ILSKFKRIMRAIQGALIVASTLQIVLGLSPLSAVPLVALVGFGLIGLPELIILVFVSFDR

FAVIFAVVIVWIYAHLLTVGGAYNCRTDRAGLIDAAPESTGAFIAVSRYASATHMPPSVL

SRGQGIAILLSGLFGTSTGSSENAGLLALTRVGSRRVVQISAGFMIFFSILGKFGAIFA

SIPGPIFASLYCLLQFCNLNSFRTKFILGFSIFMGLS

>Thecc1EG011998t2

MLGTTVIIPSSLVPQMGGKVIQTLLFVAGLNTLLQTLFGTRLPAVIGGSYTFVPTTISI

ILARFKRIMRAIQGSLIVASTLQIVLGLSPLSVVPLVSLVGFGLIGLPQLILIVFISFDR

FAVIFSVVVVWIYAHLLTVGGAYNCRTDRAGLIDAAPESTGAFIAVSRYASATPMPPSIL

SRGQGVAILVSGLFGTVNGSSENAGLLALTRVGSRRVVQISAGFMIFFSILGKFGAVFA

SIPAPIIAALYCLLQFCNLNSFRTKFILGFSVFMGLS

>GSVIVT01007809001

MLGTTVLIPTALVPQMGGKVIQTLLFVAGLNTFTQTLFGSRLPAVIGGSYTFVAATISI

ILAKFKRTMRAIQGAMIVASTLQIVLGLSPLSAVPLVSLAGFGLIGLPQLIILILVSFDR

FAVIFTVVIVWIYAHLLTVGGAYNCRTDRAGLIDAAPESTGAFIAVSRFASATHLPSSIL

SRGQGIGILLSGLFGTVNGSSENAGLLALTRVGSRRVVQISAGFMIFFSILGKFGAVFA

SIPAPIVAALYCLLQFCNLNSFRTKFILGFSIFMGFS

>Potri.008G146400.1

MLGTTVLIPTTLVPQMGGKMIQTLLFVAGLNTFLQTLFGTRLPAVIGGSYSYLPTTISI

VLAKFEKIMRGIQGALIVASTLQIVVGLSPLSTVPLVALSGFGLIGLPQIIFLLIFSFDR

FAVIFSVVIVWIYAHLLTVSGAYKCRTDRAGIIGASPESTGAFIAVSRYASATPVPPSIL

SRGQGVGILFSGIFGTGSGSSENAGLLALTRVGSRRVVQISAGFMIFFSILGKFGAVFA

SIPAPIIAALYCLLQFCNLNSFKTKFILGFSVFMGLS

>Potri.010G095500.1

MLGTTVFIPTALVPQMGGKMIQTLLFVAGLNTFFQTFFGTRLPAVIGGSFSYLPATISI

VLARFEKTMRGIQGALIVASTLQIVVGLSPLSAVPLVALSGFGLIGLPQIIFLLIFSFNR

FAVIFSVVIVWVYAHLLTVSGAYKCRTDRAGIIGAAPESTGAFIAVSRYASATPLPPSIL

SRGQGVGILFSGIFGTGSGSSENAGLLALTRVGSRRVVQISAGFMIFFSILGKFGAVFA

SIPSPIIAALYCLLQFCNLNSFKTKFILGFSVFMGLS

>Thecc1EG010231t1

MLGTTVLIPSSLVPQMGGKMIQTLLFVAGLNTLFQTLFGTRLPAVIGGSFTYVPTTISI

ILAKFEKIMRGIQGALIVASTLQIVVGLSPLSAVPLVALSGFGLIGLPQIILLLIFSFDR

FAVIFSVVIVWIYAHLLTVGGAYKCRTDRAGIIGAAPESTGAFIAVSRYASATPLPPSIL

SRGQGVGILFSGIFGTGNGSSENAGLLALTRVGSRRVVQISAGFMIFFSILGKFGAVFA

SIPPPIIAALYCLLQFCNLNSFRTKFILGFSVFMGLS

>GSVIVT01020164001

MLGTTVLIPSSLVPQMGGKVIQTLLFVAGLNTLCQTLFGTRLPAVIGGSFSFVPTTISI

VLARFEKIMRGIQGALIVASTLQIVIGLSPLSAVPLVALSGFGLIGLPQLIALVIFSFDR

FAVIFSVVLVWIYAHLLTVGGAYKCRTDRAGIIGAAPESTGGFIAVSRYASATPMPPTIL

SRGQGVGILFSGIFGTGTGSSENAGLLALTRVGSRRVVQISAGFMIFFSILGKFGAVFA

SIPPPIIAALYCLLQFCNLNSFKTKFVLGFSIFMGLS

>Solyc05g006020.2.1

MLGTTVLIPSTLVPQMGGKVIQTLLFVAGLNTLTQTLFGTRLPAVIGGSYTFVPTTLSI

VLAKFERIMRGIQGAMIVASTLQIVIGISPLSAIPLVALSGFGLIGLPQLIILLIFSFDR

FAVIFSVMIVWVYAHILTVAGTYKCRTDRAGIISGSPESTGTFFAVSRYASATPIPPSVL

SRGQGVGILFSGIFGTGTGSSENAGLLALTRVGSRRVVQISAGFMIFFSILGKFGAVFA

SIPAPIVAALYCLLQFCNLNSFRTKFILGFSIFMGLS

>PGSC0003DMP400027292

MLGTTVLIPSTLVPQMGGKVIQTLLFVAGLNTLTQTLFGTRLPAVIGGSYTFVPTTLSI

VLAKFERIMRGIQGAMIVASTLQIVIGISPLSAIPLVALSGFGLIGLPQLIILLIFSFDR

FAVIFSVMIVWVYAHILTVAGTYKCRTDRSGIISGSPESTGTFFAVSRYASATPIPPSVL

SRGQGVGILFSGIFGTGTGSSENAGLLALTRVGSRRVVQISAGFMIFFSILGKFGAVFA

SIPAPIVAALYCLLQFCNLNSFRTKFILGFSIFMGLS

>NNU_07073RA

MLGTTVIIPTALVPQMGGKVIQTLLFVAGLNTLAQTLFGTRLPAVIGGSYTFVVPTISI

ILAKFERIMRGIQGALIVASTLQIVIGISPLSAVPLVALAGFGLIGLPQLILLVVFSFDR

FAVIFSVIIVWVYAHLLTVGGAYKCRTDRSGLVESAPESTGTFIAVSRYASATPLPPSIL

SRGQGVGILFSGIFGTVNGSSENAGLLALTRVGSRRVIQISAGFMLFFSVLGKFGAVFA

SIPAPIIAAFYCVLQFCNLNSFRTKFILGFSVFLGLS

>NNU_24993RA

MLGTTVIIPTALVPQMGGKVIQTLLFVAGLNTLFQTLFGTRLPAVIGGSYTFVAPTISI

ILAKFERIMRAIQGSLIVASTLQIVIGLSPLSAVPLVALAGFGLIGLPQLILLVIFSFDR

FAVFFSVIIVWVYAHLLTVGGAYKCRTDRSGLVEAAPESTGAFIAVSRYASATPLPPSIL

SRGQGVGILFSGIFGTVNGSSENAGLLALTRVGSRRVVQISAGFMIFFSVLGKFGAVFA

SIPAPIIAAFYCLLQFCNLNSFRTKFILGFSFFMGLS

>LOC_Os03g60880.2

MLGTTVIIPTALVPQMGGRVIQTLLFVAGINTLIQSFLGTRLPAVIGGSYTFVAPTISI

ILAKFVRIMRGTQGALIVASTLQIIMGLSPLSAAPLVALVGFGLIGLPQIILLVALSFER

FAIIMSVALVWLYAFFLTVGGAYKCRTDRSGLVGGAPESTGAFIAVSRYASATPCPPSVM

SRGQGVGILLGGLFGTANGSSENAGLLGLTRVGSRRVVQISAGFMIFFSILGKFGAVFA

SIPGPIIAAIYCLLQFCNLNSFRTKFIVGFSVFMGLS

>Seita.9G030300.1.p

MLGTTVIIPTALVPQMGGRVIQTLLFVAGINTLIQSFLGTRLPAVIGGSYTFVAPTISI

ILAKFLRIMRGTQGALIVASTLQIIMGLSPLSAAPLVALVGFGLIGLPQILLLVALSFER

FAVVMSIAVVWLYAFFLTVGGAYKCRTDRSGLVGGAPESTGAFIAVSRYASATPCPPSVM

SRGQGVGILLGGLFGTANGSSENAGLLALTRVGSRRVVQISAGFMIFFSILGKFGAVFA

SIPGPIIAAIYCLLQFCNLNSFRTKFILGFSLFMGLS

>Sobic.001G031700.1.p

MLGTTVIIPTALVPQMGGRVVQTLLFVAGINTLIQSFLGTRLPAVMGASYTFVAPTISI

ILAKFVRIMRGTQGALIVASTLQIIMGLSPLSAAPLVALVGFGLIGLPQILLLVALSFER

FAVIMSITLIWLYAFFLTVGGAYKCRTDRSGLVGGAPESTGAFIAVSRYASATPCPPSIM

SRGQGVGILLSGLFGTANGTSENAGLLGLSRVGSRRVVQISAGFMIFFSILGKFGAVFA

SIPGPIIAAIYCLLQFCNLNSFRTKFILGFSLFMGLS

>Zm00001d012935_T003

MLGTTVIIPTALVPQMGGRVVQTLLFVAGINTLVQSFLGTRLPAVMGASYTFVAPTISI

VLAKFVRIMRGTQGAFIVASTLQIIMGLSPLSAAPLVALVGFGLIGLPQILLLVALSFER

FAVIMSIALIWLYAFFLTVGGAYKCRTDRSGLVGGAPESTGAFIAVSRYASATPCPPSVM

SRGQGVGILLGGIFGTANGTSENAGLLGLTRVGSRRVVQISAGFMIFFSILGKFGAVFA

SIPGPIIAAIYCLLQFCNLNSFRTKFILGFSLFMGLS

>Bradi1g03480.1.p

MLGTTVIIPTALVPQMGGRVVQTLLFVAGINTLLQSFLGTRLPAVIGGSYTFVAPTISI

VLAKFIRIMRGTQGALIVASTLQIIMGLSPLSAAPLVALVGFGLIGLPQIILLVALSFER

FAIIMSVAIVWLYAFFLTVGGAYKCRTDRSGLVAGASESTGAFIAVSRYASATPCPPSVM

SRGQGVGILLGGLFGTASGSSENAGLLGLTRVGSRRVVQISAGFMIFFSILGKFGAVFA

SIPGPIIAAIYCLLQFCNLNSFRTKFILGFSLFMGFS

>GSMUA_Achr3P06050_001

MLGTTVIIPTALVPQMGGRVVQTLLFVAGINTLFQTLFGTRLPAVIGGSYTFVVPTISI

ILAKFLRIMRGTQGALIVASTLQIIVGLSPLAVVPLVALAGFGLIGLPQIILLVIFSFDR

FSVILSVAIVWLYAYFLTVGGAYRCRTDRSGLVGGSPESTGTFIAVTRYASATPLPPSVL

SRGQGIGILLDGLFGTANGSSENAGLLALTRIGSRRVVQISAGFMIFFSILGKFGAVFA

SIPAPIFAALYCLLQFCNLNSFRTKFILGLSVFMGLS

>GSMUA_Achr9P14510_001

MLGTTVIIPTALVPQMGGRVVQTLLFVAGINTLFQTLFGTRLPAVIGGSYTFVVPTISI

ILAKFLRIMRGTQGALIVASTLQIIIGLSPLAAVPLVALAGFGLIGLPQIILLVVFSFDR

FAVIFSVTIVWLYAYFLTVGGAYKCRTDRSGLVGGSPESTGTFIAVTRYASATPLPPSVL

SRGQGIGILLDGLFGTVNGSSENAGLLALTRVGSRRVVQISAGFMIFFSILGKFGAVFA

SIPAPIFAALYCLLQFCNLNSFRTKFILGLSVFMGLS

>GSMUA_Achr5P05260_001

MLGTTVIIPTALVPQMGGRVVQTLLFVAGINTLFQTLFGTRLPAVIGGSYTFVAPTVSI

VLAKFLRVMRGTQGALIVASTLQIIVGLSPLAAVPLVVLAGFGLIGFPQIILLVIFSSDR

FAVIFSVTIVWLYAYLLTVGGAYRCRTDRSGLVGGAPESTGTFIAITRYASATPLPPSVL

SRGQGIGILLDGLFGTANGSSENAGLLALTRVGSRRVVQISALFMIFFSILGKFGAIFA

SIPAPIFAALYCLLQFCNLNSFRTKFILGFSVFMGLS

>GSMUA_Achr3P10390_001

VLGTTVLIPTALVPQMGGRVVQTLLFLAGINTLLQTYFGTCLPAVMGGSYTFVAPTISI

ILAKFLHIMRGTQGALIVASTLQIIIGLSPLSAVPLVALAGFGLVGLPVIILLVTFSFDR

FAVIFSITIVWLYAYLLTVGGAYRCRTDRSGLVGGAPESTGTFTAVARYASATPVPPSVL

SRGQGIAILLDGLFGTANGTSENAGLLALTRVGSRRVVQISAGFMIFFSILGKFGAVFA

SIPAPIFAALYCLLQFCNLNSFRTKFILGFSFFMGLS

>GSMUA_Achr8P25880_001

MLGTTVIIPTALVPQMGGRVIQTLLFVAGINTLFQTFFGTRLPAVMGGSYTFVMPTISI

ILAKFLRIMRGTQGALIVASTLQIIIGLSPLSAVPLVALAGFGLIGLPVIVLLVIFSFDR

FAVIFSIVIVWLYAYLLTVGGAYRCRTDRSGLVGGAPESTGTFMAVARYASATPVPPSVL

SRGQGIGILLDGLFGTANGSSENAGLLALTRVGSRRVVQLSAGFMIFFSILGKFGAVFA

SIPAPIFAALYCLLQFCNLNSFRTKFILGFSVFMGLS

>GSMUA_Achr9P18290_001

MLGTTVIIPTALVPQMGGRVIQTLLFVAGINTLLQTYFGTCLPAVMGGSYTFVVPTISI

ILAKFLHIMRGTQGALIVASTLPIIVGLSPLSAVPLVALAGFGLIGLPVIILLVIFSFDR

FAVIFSIAIVWVYAYFLTIGGAYRCRTDRSGLVGGAPESTGTFIAVARYASATPVPPSVL

SRGQGIGILLDGLFGTANGSSENAGLLALTRVGSRRVVQISAGFMIFFSILGKFGAVFA

SIPAPIFAALYCLLQFCNLNSFRTKFILGFSVFMGLS

>Solyc03g114030.2.1

MLGTAVIITTALVPQMGGKVIQTVLFVAGLNTLLQSYFGTRLPAVIGASYTFVAPTISI

ILSRFKKIMRATQGALIVASTIQIVLGLSPLSAVPLVALVGFGLIGLPELVLLVIFSFDR

FAVLFTVAIVWIYAHLLTVGGAYNCRTDRAGLIDGAPESTGAFIATTRYASATPLPPSVL

SRGQGIGILLSGLFGTGNGSSENVGLLALTRVGSRRVVQIAAGFMIFFSILGKFGAVFA

SIPTSIVGALYCILQFCNLNSFRSKFILGFSIFLGLS

>PGSC0003DMP400042557

MLGTAVIITTALVPQMGGKVIQTVLFVAGLNTLLQSYFGTRLPAVIGASYTFVAPTISI

ILSRFKKIMRATQGALIVASTIQIVLGLSPLSAVPLVTLVGFGLIGLPELVLLVIFSFDR

FAVLFTVAIVWIYAHLLTVGGAYNCRTDRAGLIDGAPESTGAFIATTRYASATPLPPSVL

SRGQGIGILLSGLFGTGNGSSENVGLLALTRVGSRRVVQIAAGFMIFFSILGKFGAVFA

SIPTSIVGALYCILQFCNLNSFRSKFILGFSIFLGLS

>Solyc12g026430.1.1

MLGTTVIIPSALVPQMGGKVIQTVLFVSGLNTLLQTSFGTRLPAVIGGSYTFVAPTISI

ILSRFKKIMRATQGALIVASTLQIVLGLTPLSAVPLVSLVGFGLVGLPQLVLLVIFSFDR

FAVLFTVAIVWIYAYILTVAGAYNCRTDRAGLIGAAPESTGAFIAVARYASATPMPPSIL

SRGQGIGILLSGLFGTGNGSSENAGLLALTRVGSRRVVQISAGFMIFFSILGKFGAVFA

SIPAPIVGALYCLLQFCNLNSFRNKFILGFSIFLGLS

>PGSC0003DMP400033653

MLGTTVIIPTALVPQMGGKVIQTVLFVSGLNTLLQTSFGTRLPAVIGGSYTFVAPTISI

ILSRFKKIMRATQGALIVASTLQIVLGLTPLSAVPLVSLVGFGLVGLPQLVLLVIFSFDR

FAVLFTVAIVWIYAYILTLAGAYNCRTDRAGLIGAAPESTGAFIAVARYASATPMPPSIL

SRGQGIGILLSGLFGTGNGSSENAGLLALTRVGSRRVVQISAGFMIFFSILGKFGAVFA

SIPAPIVGALYCLLQFCNLNSFRNKFILGFSIFLGLS

>Solyc06g071330.2.1

MLGTIVIIPTALVPQMGGQVIQTSLFVAGLNTLLQSIFGTRLPAVIGGSYTFVAPTISI

ILSKFKKIMRATQGALIVASTLQIVLGLSPLSAVPLVSLVGFGLIGLPELVLLVIFSFDR

FAVLFTVIIVWIYAHLLTVGGAYNCRTDRAGLISGAQESTGAFIAVARYASATPLPPSIL

SRGQGVGILLSGLFGTGNGSSENAGLLALTRVGSRRVVQISAAFMIFFSILGKFGAVFA

SIPAPIVGALYCLLQFCNLNSFRTKFILGFSVFLGLS

>LOC_Os02g50820.1

MLGTTVIIPTALVPQMGGVVIQTLLFVAGINTLLQSFFGTRLPAVIGGSYTFVVPTISI

ILAKFLRIMRGTQGALIVASALQIIFGLSPLSAAPLVMLVGFGLIGLPELILLVIFAFDR

FAVLFTIPIVWLYAYLLTVGGAYRCRTDRSGIIGGAPESTGAFIAVSRYASATPLPPSVL

SRGQGIGILLDGLFGTGNGSSENAGLLALTRVGSRRVVQISAGFMIFFSILGKFGAVFA

SIPPPIFAALYCILQFCNLNSFRTKFILGFSVFMGLS

>Sobic.004G242200.1.p

MLGTSVIIPTALVPQMGGVVIQTLLFVAGINTLLQSFFGTRLPAVVGGSYTFVLPTISI

ILAKFLRIMRGTQGALIVASALQIIVGLSPLSAAPLVALVGFGLIGLPELILLVIFAFDR

FAVLFTIPIVWLYAYLLTVGGAYRCRTDRSGLIGGAPESTGAFIAVSRYASATPIPPSVL

SRGQGIGILLDGLFGTGNGSSENAGLLALTRVGSRRVVQISAGFMIFFSILGKFGAVFA

SIPAPIFAALYCILQFCNLNSFRTKFILGFSVFMGLS

>Seita.1G316400.1.p

MLGTTVIIPTALVPQMGGVVIQTLLFVAGINTLLQSFFGTRLPAVIGGSYTFVVPTISI

ILAKFLRIMRGTQGALIVASALQIIVGLSPLSAAPLVALVGFGLIGLPELILLVIFAFDR

FAVLFTIPIVWLYAYLLTVGGAYRCRTDRSGLIGGAPESTGAFIAVSRYASATPIPPSVL

SRGQGIGILLDGLFGTGNGSSENAGLLALTRVGSRRVVQISAGFMIFFSILGKFGAVFA

SIPAPIFAALYCILQFCNLNSFRTKFILGFSVFMGLS

>Zm00001d018001_T001

MLGTTVIIPTALVPQMGGVVIQTLLFVAGINTLLQSFFGTMLPAVIGGSYTFVLPTISI

ILAKFLRIMRGTQGALIVASALQIIVGLSPLSAAPLVALVGFGLIGLPQLILLVIFTFDR

FAVLFTIPIVWLYAYLLTVGGAYRCRTDRSGLIGGAPESTGAFIAVSRYASATPIPPSVL

SRGQGIGILLDGLFGTGNGSSENAGLLALTRVGSRRVVQISAGFMIFFSILGKFGAVFA

SIPAPIFAAMYCILQFCNLNTFRTKFILGFSVFMGLS

>Bradi3g59600.1.p

MLGTTVIIPTALVPQMGGIVIQTLLFVAGINTLLQSFFGSRLPAVIGGSYTFVLPTISI

ILAKFLRIMRGTQGALIVASALQIIVGLSPLSAAPLIALVGFGLIGLPELILLLIFAFDR

FAVLFTIPIVWLYAYLLTVGGAYRCRTDRSGLIGSAPESTGSFIAVSRYASATPLPPSVL

SRGQGIGILLNGLFGTANGSSENAGLLALTRVGSRRVVQISAGFMIFFSILGKFGAVFA

SIPAPIFAALYCVLQFCNLNSFRTKFILGFSLFMGLS

>el__p5_sc00044.V1.gene532

MLGTTVIIPTALVPQMGGRVIQTLLFVAGINTLLQTFVGTRLPAVIGGSYTFVVPTISI

ILAFDR

FAVLFSVAIVWLYAFILTVGGAYRCRTDRSGLVGGAPESTGTFIAVARYSSATPLPPSIL

SRGVGL

>GSMUA_Achr2P05370_001

MLGTTVIIPTALVPQMGGRVIQTLLFVAGLNTLLQTTFGTRLPAVIGGSYTFVVPTISI

ILARFLHVMRGTQGALIVSSTIQIILGLSPLSAAPLVALAGFGLIGLPQLIILVIFSFDR

YAVLFSIAIVWLYAFILTVGGAYKCRTDRSGLIASSPESTGAFIAVSRYSSATPVPPSIL

GRGQGIGILLDGIFGTANGSTENAGLIALTRVGSRRVVQISAGFMIFFSILGKFGAVFA

SIPGPIIAALYCLLQFCNLNSFRTKFILGFSVFMGLS

>GSMUA_AchrUn_randomP11370_001

MLGTTVIIPTVLVPQMGGRVIQTFLFVAGLNTLLQTLFGTRLPAVIGGSYTFVVPTISI

ILNRFLRTMRGTQGALIVASVVQIIIGLSPLSAAPLVALAGFGLIGLPQLIVLVIVSFDR

FAVIFSVAIVWLYAFILTVGGAYRCRTDRSGLVAASPESTGTFIAVSRYASATQVPPSIL

SRGQGIGILLDGIFGTANGSTENAGLLALTRVGSRRVVQISAGFMIFFSILGKFGAVFA

SIPGSIIAALYCLLQFSNLNSFRTKFILGFSVFLGLS

>LOC_Os08g32500.1

MLGTSVIIPSALVPQMGGRVIQTLLFVAGINTLCQSFFGTRLPAVMGGSYTIVAPTISI

ILAKFLRTMRGTQGALIIASTIQIILGLSPLSAVPLISLAGFGLIGLPEIILLLVFSFDR

FAVIFTIAIVWLYAYILTASGAYKCRVDRSGIISGAPESTGTFIAVSRYASATMIPPSVL

GRGQGIGTLIGAFFGTANGTAENAGLLALTHVGSRRVVQISAGFMIFFSILGKFGAIFA

SIPLPIFAALYCILQFCNLNSFRTKFIVGFSFFMGLS

>Sobic.007G129000.1.p

MLGTSVIIPSALVPQMGGRVIQTLLFVAGINTLCQSFFGTRLPAVMGGSYTIVAPTISI

IMAKFLRTMRGTQGALIIASTIQIILGLSPLSAVPLVSLAGFGLIGLPEIILMLIFSFDR

FSVIFTIAIVWLYAYILTVSGAYKCRVDRSGLISGAPESSGTFIAVSRYASATIIPPSIL

GRGQGIGTLLGAFFGTANGTAENAGLLALTHVGSRRVVQISAGFMIFFSILGKFGAIFA

SIPLPIFAALYCILQFCNLNSFRTKFIMGFSLFMGLS

>Zm00001d032322_T001

MLGTSVIIPSALVPQMGGRVIQTLLFVAGINTLCQSFFGTRLPAVMGGSYTIVAPTISI

IMAKFLRTMRGTQGALIIASTIQIVLGLSPLSAVPLVSLAGFGLIGLPEIILMLIFSFDR

FSVIFTIAIVWLYAYILTVSGAYKCRVDRSGLVGGAPESSGAFIAVSRYASATMIPPSVL

GRGQGIGTLLGAFFGTANGTAENAGLLALTHVGSRRVVQISAGFMIFFSVLGKFGAIFA

SIPLPIFAALYCILQFCNLNSFRTKFIMGFSLFMGLS

>Seita.6G149300.1.p

MLGTSVIIPSALVPQMGGRVIQTLLFVAGINTLCQSFFGTRLPAVMGGSYTVVAPTISI

IMAKFLRTMRGTQGALIIASTIQIILGLSPLSAVPLISLAGFGLIGLPEIILMLIFSFDR

FSVIFTIAIVWLYAYILTASGAYKCRVDRSGLISGAPESTGTFIAVSRYASATMIPPSVL

GRGQGIGTLLGAFFGTANGTAENAGLLALTHVGSRRVVQISAGFMIFFSILGKFGAIFA

SIPLPIFAALYCILQFCNLNSFRTKFILGFSLFMGLS

>Bradi3g36010.1.p

MLGTSVIIPSALVPQMGGRVIQTLLFVAGINTLFQSFFGTRLPAVMGGSYTVVAPTISI

ILAKFLRTMRGTQGAFIIASTIQIILGLSPLSAVPLISLAGFGLIGLPEIILLLIFSFDR

FAVIFTIAIVWLYAYILTVSGAYNCRVDRSGLIGGAPESTGTFVAVSRYASATMIPPSIL

GRGQGIGTLLGAFFGTANGTAENAGLLALTHVGSRRVVQISAGFMIFFSILGKFGAIFA

SIPLPIFAALYCILQFCNLNSFRTKFIVGFSFFMGLS

>LOC_Os09g21340.1

MLGTTVIIPSALVPQMGGRVIQTLLFVAGINTLFQTFFGSRLPVVMGGSYTFVAPTISI

ILAKFLRTMRGTQGALIIASTIQMILGLSPLSAVPLISLVGFGLIGLPELILLVAFSFGR

FGVLFTVSIVWLYAYILTISGAYKCRVDRSGLISGAPETTGAFIAASRYASATMIPPSII

SRGQGISILIDSFFGTANGTSENVGLLALTHVGSRRVVQISAGFMIFFAILGKFGALFA

SIPLPIFAGMYCILQFCNLNSFRTKFILGFAFFMGIS

>Bradi4g29440.1.p

MLGTTVIIPSALVPQMGGRVIQTLLFVAGINTLLQTFFGSCLPVVMGGSYTFVAPTISI

ILAKFLRTMRGTQGALIIASTIQIILGLSPLSAVPLVSLVGFGLVGLPELILMVAFSFGR

FAVLFTVSIVWLYAYILTISGAYKCRVDRSGLIAGAEESTGAFIAASRYASATMIPPSIV

SRGQGIGILLDSFFGTANGTSENVGLLAVTHVGSRRVVQISAGFMIFFAVLGKFGALFA

SIPLPIFAGMYCVLQFCNLNSFRTKFILGFAFFMGIS

>Sobic.002G189700.1.p

MLGTTVIIPSSLVPQMGGRVVQTILFVAGINTLFQTFFGTRLPVVMGGSYIFVGPTISI

ILAKFLRTMRGTQGALLIASTIQIILGLSPLAAVPLVSLVGFGLIGLPEVFLLVVFSFSR

FSVLFTVSIVWLYAYILTIGGAYKCRVDRSGLISGAPESTGAFIGASRYASATMIPPSII

SRGQGIGLLLDSFFGTATGTSENIGLLALTRIGSRRVVQISAGFMIFFSVLGKFGALFA

SIPLPIFAGMYCLLQFCNLNSFRTKFIMGFAFFMGLS

>Zm00001d020345_T001

MLGTTVIIPSALVPQMGGRVVQTILFVAGINTLFQTLFGTRLPVVMGGSYVFVGPTISI

VLAKFLRTMRGTQGALLVASTIQIILGLSPLAAVPLVSLVGFGLVGLPELLLLVVFSFSR

FSVLFTVAIVWLYAYILTIGGAYKCRVDRSGLISGAPESTGAFIGASRYASATMIPPSII

SRGQGIGLLLDSFFGTANGTSENIGLLALTRIGSRRVVQISAGFMIFFSVLGKFGALFA

SIPLPVFAGMYCLLQFCNLNSFRTKFIMGFAFFMGLS

>Seita.2G193900.1.p

MLGTTVIIPSALVPQMGGRVVQTILFVAGINTLFQTFFGTRLPVVMGGSYTFVAPTISI

ILAKFLRTMRGTQGALIIASTIQIILGSSPLSAVPLVSLVGFGLVGLPELLLLVVFSFGR

FGVLFTVPIVWLYAYILTIGGAYKCRVDRSGLVGGAPESTGAFIGASRYASATMIPPSII

SRGQGIGILLDSFFGTANGTSENIGLLGLTRVGSRRVVQISAGFMIFFSVLGKFGALFA

SIPLPIFAGMYCLLQFCNLNSFRTKFIMGFAFFMGLS

>Zm00001d034678_T001

MLGTTVIIPTALVPQMGGRVVQTLLFVAGINTLIQSFLGTRLPAVMGGSYTFVAPTISI

ILARLAVLPASSSKFRLSLSPLSAAPLVALVGFGLIGLPQILLLVALSFER

FAVIMSVVLIWLYAFFLTVGGAYKCRTDRSGLVGGAPESTGAFIAVSRYASATPCPPSVM

SRGQGVGILLGGLFGTANGTTENAGLLALTRVGSRRVVQISAGFMIFFSILGKFGAVFA

SIPGPVIAAVYCLLQFCNLNSFRTKFILGFSLFMGLS

>el__p5_sc00047.V1.gene389

MLGTTVLIPTILVPQMGGRVIQTLLFVAGINTLLQGFFGTRLPAVIGGSFTYLLPTISI

ILSRFVHTMRAIQGALIAASSFQIIVG

ESTGTLIAVSRFASATPVPPAIF

SRGQGIGILLDGMFGTAN

>GSMUA_Achr7P26110_001

MLGTTVIIPTILVPLMGGRVIQTFLFVAAINTLTQVYFGTRLSAVIGGSYTYLLPTISI

ILSRFEHTMRAIQGAFIAASSFQIIVGLSPLAAVPFVTLSALGLLGLPAIILLILFAFDR

FALLIVVAIVWLYAYILTVAGAYKCRTNRSGLIGASPESTGTLIAVSRLSSATPVPPSVF

SRGQGIGILLDGMFGTANGSAENAGLLGLTRVGSRRVIKISACFMFFFSILGKFGALLA

SIPLSIFAALYCVLQFCNINSFRTKFILGFSFFMGLS

>NNU_13781RA

VLGTTVLIPSIIVPQMGGKVIQSLLFVAGLNTLLQSLFGTRLPTVIGGSCTFVVPTISI

ALARFIRTMRGIQGALICASIFQIVVGLSPLSAVPLVTLTGLGLVGIPELVILVLVSFDR

FVVPFSVAVVWVYAHILTVAGAYKCRTDHSGLLRTAPESTGTFIAVSRYGSATPVPPSVL

SRGQGIGILLDGMFGTGNGSTGNAGLLALTRVGSRRVVQISAGFMLFFSVLGKFGALFA

SIPMSIVAALNCILQFCNLNSFRTKFILGFSFFMGFS

>AT1G49960.1

MLGTTVIIPSILVPLMGGEVINTVLFVSGINTLLQSLFGSRLPVVMGASYAYLIPALYI

TFSRFEETMRAIQGALIIASISHMIMGLSPLSAAPLVILTGVGLIGLPALIILIILSCEQ

FAVLFTIAIVWAYAEILTAAGAYDCRTDRSGLISASPETTGSFIAASRFGSATHIPPSVL

SRGQGIGVLLNGLFGTATGSTENTGLLGLTKVGSRRVVQISAGFMIFFSIFGKFGAVLA

SIPLPIFAALYCVLQFCNLNSFRNKFILGFSIFIGLS

>Potri.009G086800.1

MLGTSVIIPSIVVPLMGGEMINTLVFVAGINTLLQTWLGTRLPVVIGGSYAFIIPTITI

ALSRFKQSMRAVQGAIIIASFFQMIIGLSPLAAVPLVILTGLGLIGLPALLLVVFISYSR

YAVLFSVAVVWAYAAVLTVAGAYNCRVDRSGLIGAAPESTGTIIATYQYGSATHLPPSVF

GRGLGIGTLLDGLFGTGNGSTENAGLVGLTRVGSRRVIQISAGFMLLFSVLGKFGAVLA

SIPLPIMAALYCVLQFCNLNSFRTKFILGFSLFLGLS

>Thecc1EG007245t1

MLGTTVIISSILVPLMGGEVINTILFVAGINTLLQTLFGTRLPVVIGGSYAFVIPIISI

ALSRFKHSMRDVQGALIVASFFTMVIGLSPLAAVPLVILTGLGLVGLPALVAVVFLSFDR

FAILFSVGIIWAYAEILTAAGAYDCRTDRSGLISAAPESTGTFIAASRYGSATPMPPSVL

SRGLGVGILLDGLFGTGSGSTENAGLLGLTRVGSRRVVQISAIFMLFFSVLGKFGAVLA

SIPLPIVAALYCVLQFCNLNSFRTKFILGFSLFMGLS

>GSVIVT01037811001

MLGTTIIITGIFVPKMGGEVIQTVLFVAGLNTLLQTWFGTRLPVVMGASYTFIIPIYSI

VLARFKETMRGIQGALLIASLFPMIAGLSPLSAVPLVTLTGLGLVGLPQLILLVFLSFDR

FAVLFSVALVWVYAEVLTVAGAYDCRTDRSGLITAAPESTGTFIAASRYSSATPIPPSIL

SRGQGVAILLDGMFGAVSGSTENTGLLGLTRVGSRRAIQISAGFMLFFSVLGKFGAIFA

SIPLPIVAAIYCILQFCNLNSFRSKFILGFSLFMGLS

>Solyc01g106920.2.1

MLGTSVIIPTIIVPQMGGQVIQTLLFVAGVNTLLQSWFGTRLPVVIGGSFRFIIPAVFI

ALSRFKQSMRSMQGALMIASVIPALAGLCPLSKAPLVLLVGIGLIGLPELVLLILLSFER

FAVLLSVGIVWAFAALLTVAGAYKCRVDRSGLVSGASESTGGFIAAARYGSATFCPASVL

SRGLGLSTLINGLWGTPSGSTENVGLLALTRVGSRRVIQISAVFMLFFSVLGKFGAILA

SIPLPIIGALYCVLQFCNLNSYRTKFILGFSIYMGLS

>PGSC0003DMP400044823

MLGTSVIIPTIIVPQMGGQVIQTLLFVAGVNTLLQSWFGTRLPVVIGGSFRFIIPAVFI

ALSRFEQSMRSMQGALMIASVIPVLAGLSPLSKAPLLLLVGIGLIGLPELVLLILLSFER

FAVLLSVGIVWAFAALLTVAGAYTCRVDRSGLVSGASESTGGFIAAARYGSATFCPASVL

SRGLGLSTLINGLWGTPSGSTENVGLLALTRVGSRRVIQISAVFMLFFSVIGKFGAILA

SIPLPIIGALYCVLQFCNLNSYRTKFILGFSIYMGLS

>Solyc10g049280.1.1

MLGTTVIIPTIIVPQMGGQVIQTLLFVAGLNTLLQSLFGTRLPVVIGGSFTFIIPATFV

ASSRFIHSMRGMQGALMIASILPILIGLSPLSAAPPVFLVGLGLIGLPGLIILLLLSFER

FAVLLSVAIVWAYAALLTVTGAYNCRVDRSGLISGASESTGAFIAAARYGSVTHTPGSVI

SRGLGLGLLLSGLWGTASGFTENVGLVAMTRVGSRRVIQMSAIFMLFFSVLGKFGAVLA

SIPLPIVGALYSILQFCNLNSFRTKFILGLSIYLGFS

>PGSC0003DMP400005826

MLGTTVIIPTIVVPQMGGQVIQTLLFVAGLNTLLQSLFGTRLPVVIGGSFTFIIPATFV

ALSRFIHSMRGMQGALMIASILPILIGLSPLSAAPPVLLVGLGLIGLPGLIILILLSFER

FAVLLSVAIVWAYAAILTVTGAYNCRVDRSGLISGASESTGAFIAAARYGSVTHTPGSVI

SRGLGLGILLSGLWGTASGSTENVGLLAMTRVGSRRVIQMSAIFMLFFSVLGKFGAVLA

SIPLPIVGALYCVLQFCNLNSFRTTFILGLSIYLGLS

>AT1G65550.1

SLGITVLIPSVLVPLMGGKVIQTLLFVSGLTTLFQSFFGTRLPVIAVASYAYIIPITSI

IYSRFVRTMRSIQGALIITGCFQVLICLSPLSIAPLATFTGLGLVGLPGLILLIFVTCDR

YGMILCIPLVWLFAQLLTSSGVYDCRTDRTGLITNTPESTGLFYASARYGSATPIPPSVV

SRGLGVGVLLNGMLGGITGITENVGLLAMTKIGSRRVIQISAAFMIFFSIFGKFGAFFA

SIPLPIMASLYCILQFCNLNSFNIKFILGFSFFMAIS

>AT5G25420.1

SLGITVLIPSLLVPLMGGKVIQTLLFVSGLTTLFQSFFGTRLPVIASASYAYIIPITSI

IYSRFVRTMRSIQGALIITGCFQVLVCLSPLSIAPLVTFTGLGLCDR

YGMMLCIPVVWLFAQLLTSSGVYDCRTDRTGLITNTPESTGLFYASARYG

KNVGLLAMTKVGSRRVIQISAAFMLFFSIFGKFGAFFA

SIPLPIMASLYCILQFCNLNSFNTKFILGFSFFMAIS

>Potri.014G015100.1

TLGMTVMIPSIIVPRMGGRVIQTLLFTSGLSTLFQTLFGTRLPSVAVGSYAYMIPTTSI

VLARFVQTMRAIQGALIIAGCFQIIMGLSPISIVPCVTFAGLGLIGLPGMLIMVFFSCDR

FAVLLTAAIAWLFAQILTASTVYNCRTDRVGLIHASPESTGTFYATSRYGSATPVPPSVV

SRGLGIGVLLNGFFGCVTGFTENAGLLALTKVGSRRVIQISAGFMIFFSLFGKFGAFFA

SIPLPIIAAVYCVLQFCNLNSFRTKFILGFSFFIGIS

>Thecc1EG019285t1

TLGITVLIPSMIVPQMGGTVIQTLLFVSGLSTLLQSFFGTRLPTVAVGSYAYLIPVTSI

IQARFVWTMRGIQGALIIAACFQSVMGLSPLSVVPYVTFTGLGLIGLPAIIVMVFISYDR

FAVLFSVAITWLFAQLLTSASVYKCRTDRVGLLSSAPESTGTFFAAARYGSATPVPPSVI

SRGLGIGVLLNGLLGSVTGTTENTGLLALTRVGSRRVIQISAGFMIFFSIFGKFGAFFA

SVPLPILAALSCVLQFCNLNSFRTKFILGFSFFMGIS

>GSVIVT01009328001

TLGITVLIPSILVPQMGGRAIQTLLFVSGLNTLLQSFFGTRLPNMVVGSYAFLVPATSI

LLSRYEQTMRGIQGALIATSFFQMIVGISPLSAVPLVTSTAVGLVGCPELILMVFISYDR

YAMLFSVPIVWSYAHILTASGVYDCRTDRSGLVGGSPESTGTFIATSRYGSATPIPPSVI

SRGLGIGVLLNGFFGAVTGSTENVGLLAVTRVGSRRVIQISAGFMIFFSVLGKFGAVFA

SIPLPIIAALYCVLQFCNLNSFRTKFILGFSIFMGFS

>LOC_Os03g48810.1

MLGTTVIVATILVPLMGGIVVQTILFLAGINTLLQVHLGTRLPAVMGASYAYIYPAVAI

ILSRFVYTMRSLQGALIIAGVVQAIIGLSPLAAVPFVTLSALGLVGLPALILLLLFTFGR

CAVLATVLVVWIYAEILTAAGAYNCRADRSGLIHGAPESTGTLMAVTRYAGATFCPPSVF

ARGQGISTILDGMCGTLTGSVENAGLLALTRVGSRRVIKISALFMIFFSLFGKFGAIIA

SIPLPIFSALYCVLQYCNLNTLRTKFILSISLFLGLS

>Bradi1g11910.1.p

MLGTTVIIATILVPLMGGVVIQTILFLSGINTLLQVHFGTRLPAVMGGSYTYIYPTVAI

ILSRFVYTMRSLQGALIIAGVFQVVVGLSPLAAVPFVTLSALGLIGLPALILLLIFAFGR

CAVLLTVIIVWIYAEILTAAGAYNCRTDRSGLIHAAPESTGTLIAVSRYAGATFVPPSVF

ARGQGISIILNGMCGTLTGTAENSGLLAITRVGSRRVIKISALFMIFFSLFGKFGAILA

SIPLPIFSALYCVLQYCNLNTLRTKFILSISLFLGLS

>Sobic.001G129600.1.p

MLGTTVLIATIIVPLMGGIVIQTILFLSGINTLLQVHFGTRLPAVMSGSYTYIYPAVAI

ILSRFIFTMRSLQGALIIAGVFQAVVGLSPLAAVPFVTLSGLGLVGLPALVLVVIFAFGR

CAVLVTIIVVWIYAEILTAAGAFNCRSDRAGIIEGSPESTGTLIAVSRYAGATFTPPSVF

SRGEGISIILDGMCGTLTGTAENAGLLAVTRVGSRRVVKIAALFMIFFSLFGKFGAVLA

SIPLPLFAAVYCVLQYCNLNSLRTKFILSISIFLGLS

>Zm00001d033585_T002

MLGTTVLIATIIVPLMGGIVIQTILFLSGINTLLQVHFGTRLPAVMSGSYTYIYPAVAI

ILSRFVFTMRSLQGALIIAGVFQAVVGLSPLAAVPFVTLTGLGLVGLPALVLLVIFAFSR

CAVLVTVVIIWIYAEILTAAGAYNCRADRSGIIQGSPESTGTLIAVSRYSGATFCPPSVF

SRGEGISIILDGMCGTLTGTAENAGLLAVTRVGSRRVIKISALFMIFFSLFAKFGAVLA

SIPLPIFAALYCVLQYCNLNSLRTKFILSISLFLGLS

>Seita.9G131100.1.p

MLGTTVIIPTIIVPLMGGIVIQTILFLAGINTLLQVHFGTRLPAVMGGSYTYIYPIVAI

VLSRFVLTMRSIQGALIIAGVFQAVVGLSPLAAFPFVTLSGLGLIGLPALVLLVLFAFGR

CAVLVTIIIVWIYAEILTAAGAYNCRTDRAGIIQGSPESTGTLIVVSRFAGATFCPPSVF

SRGEGISIILDGMCGTLTGTAENAGLLPLTRVGSRRVIKISALFMIFFSLFGKFGAVLA

SIPLSLFAALYCVLQYCNLNSLRSKFIISISLFLGLS

>AT2G05760.1

MLGTSAFIPALLVPAMGGRVIQTLLFVAGIKTLLQALFGTRLPAVVGGSLAYVVPIAYI

INDRFIHTMRAIQGALIVASSIQIILGFSPLGMAPVVGLVGLGMIGLPMLLLVIGLTFER

FPILICVTIVWIYAVILTASGAYRCRTDKANLISTAPESTGAYIAASRLAIATPPPAYVL

SRGQGIGVLLDGLFGTGTGSTENVGLLGLTRVGSRRVVQVSAGFMIVFSTLGKFGAVFA

SIPVPIYAALHCILQFTNMNSMRNLMITGLSLFLGIS

>Potri.014G157800.1

MLGTSVMIPSVLVPAMGGRVIQTLLFVAGINTLLQALFGTRLPAVVGGSYAYVVPIAYI

IRDRFIQTMRAIQGALIVASSIQIILGFSPLGMAPVVGLVGLGLIGIPMLLLVIGLSFER

FPVLICIAFVWIYAIILTASGAYRCRTDRANLISTAPESTGAYKAASRLAIATPPPAYVL

SRGQGIGILLDGLFGTGTGSTENVGLLGLTRVGSRRVVQISAGFMIFFSILGKFGAVFA

SIPFPIFAALYCVLQFTNMNSMRNLIITGLSLFLGIS

>Thecc1EG002164t1

LLGTSVMIPSLLVPAMGGRVIQTLLFVAGINTLLQALFGTRLPAILGGSYAYVIPIAYI

INDRFIQTMRAIQGALIVASSIQIILGFSPLGMAPVVALVGLGLIGLPMLLLVIGVSFER

FPVLICVAIIWIYSLILTASGAYRCRTDRANLISSAPESTGAYKAASRLAIATPPPAYVL

SRGQGIGILLDGLFGTCTGSTENVGLLGLSRVGSRRVVQLSAGFMMFFSTLGKFGAVFA

SIPFPIFAALYCVLQFTNMNCMRNLIITGLSLFLGLS

>GSVIVT01030497001

VLGTSVMIPSLLVPVMGGRVIQTLLFVAGINTLLQALFGTRLPAVVGGSFAYIIPIVYI

ISDRFIHTMRAIQGALIVAASIQIILGFSPLGMAPVVGLVGLGLIGIPMLLLVIGVSFER

FPVLICVTIVWIYALILTASGAYRCRTDKANLISSAPESTGAYKAASRLAIATPPPAYVL

SRGQGIGILLDGLFGTCTGSTENVGLLGLTRVGSRRVVQISAGFMIFFSMLGKFGAVFA

SIPFPIFAALYCVLQFTNMNSMRNLIITGLSLFLGIS

>NNU_20362RA

TLGTSVMIPSLLVPVMGGRVIQTLLFVSGIKTLLQALFGTRLPAVVGGSFAYVIPILYI

IGDRFIQTMRAIQGALIVASSIQIILGFSPLGMAPVVGLVGLGLIGIPMLLAVIGLTFER

FPVLICVSIIWAYAHVLTAGGAYRCRTDRANLIYSAPESTGAYKAASRLAIATPPPAYVL

SRGQGIGVLLDGLFGTGTGSTENVGLLGLTRVGSRRVVQISAGFMIFFSILGKFGALFA

SIPFPIFAALYCVLQFTNMNSMRNLFITGLSLFLGIS

>Solyc07g049320.2.1

VLGTSVMIPSALVPLMGGRVIQTLLFVAGINTLLQALFGTRLPAVVGGSFAYVIPIVYI

ISDRFVHTMRAIQGALIVAASIQIILGFSPLGMAPVVGLVGFGLIGLPMLLLVIGLSFER

FPVLICVSIIWIYSIILTASGAYHCRTDRANLISTAPESTGAYMAASRLAIATPPPAYVL

SRGQGIGILLDGLFGTCTGSTENVGLLGLTRVGSRRVVQISAGFMIFFSMLGKFGAVFA

SIPFPIYAALYCVLQFTNLNCMRNLIITGLSLFLGIS

>PGSC0003DMP400001358

VLGTSVMIPSALVPLMGGRVIQTLLFVAGINTLLQALFGTRLPAVVGGSFAYVIPIVYI

ISDRFVHTMRAIQGALIVAASIQIILGFSPLGMAPVVGLVGFGLIGLPMLLLVIGLSFER

FPVLICVSIIWIYSIILTASGAYHCRTDRANLISTAPESTGAYMAASRLAIATPPPAYVL

SRGQGIGILLDGLFGTCTGSTENVGLLGLTRVGSRRVVQISAGFMIFFSMLGKFGAVFA

SIPFPIYAALYCVLQFTNLNCMRNLIITGLSLFLGIS

>GSMUA_Achr4P09490_001

MLGTSVMIPSLLVPLMGGRVIQTLLFVSGINTLLQALFGTRLPTVVGGSFAYVIPILYI

IRDRFLQTMRAIQGALIIASSLQIILGFSPLGMAPVIGLVGLGLIGIPMLLMLIGLSFER

FPVLICVTVIWIYSLILTAGGAYNCRTDRANLISSAPESTGAYKAASRLAIATPPPAYVL

SRGQGIGVLLDGLFGTGTGSTENVGLLGLTRVGSRRVVQISAGFMIFFSTLGKFGAVFA

SIPFPIFAALYCVLQFTNMNSMRNLIITGLSLFLGIS

>GSMUA_Achr8P25270_001

MLGTSVMIPSMLVPLMGGRVIQTLLFVSGINTLLQALFGTRLPTIVGGSFSYVIPILYI

IRDRFLQTMRAIQGALIIASSLQIIIGFSPLGMAPVIGLVGLGLIGIPMLLMLIGLSFER

FPVLICVTIIWIYSLILTAGGAYNCRTDRANLISSAPESTGAYKAASRLAIATPPPAYVL

SRGQGIGVLLDGLFGTGTGSTENVGLLGLTRVGSRRVVQISAGFMIFFSTLGKFGAVFA

SIPFPIFAALYCVLQFTNMNSMRNLIITGLSLFLGIS

>el__p5_sc00037.V1.gene489

FVL

FER

FPVLICVTVIWIYSLILTAGGAYNCRTDRANLISSAPESTGAYMAASRLAIATPPPAYVL

SRG

>AT2G34190.1

ALGTAVMIPSILVPMMGGRVVQTLLFLQGVNTLLQTLFGTRLPTVIGGSYAFMVPIISI

IHDRFLSTMRAVQGAIIVASSVQIILGFSPIGMVPVIALTGFGLIGLPMLILFVIFSVER

FALIIALIIVWAYAHVLTASGAYKCRTDMSNLISSAPESTGAFKAAARLASATPPPPHVL

SRGQGIGILLNGLFGTLSGSSENIGLLGSTRVGSRRVIQISAGFMIFFSMLGKFGALFA

SIPFTIFAAVYCVLQFTNMNSLRNLFIVGVSLFLGLS

>Potri.004G058800.1

ALGTAVMIPSFLVPLMGGRVVQTLLFVEGINTLLQTLFGTRLPTVIGGSYAFMVPIVSI

IHDRFLSTMRAVQGALIVSSSIQIILGFSPIGMVPVIALVGFGLIGIPMLILFITCSLER

FALLISITVIWAYAHLLTASGAYKCRTDKAYLISSAPESTGAYKAASRLASATPPPAHVL

SRGQGIGILLDGLFGTLTGSTENIGLLGSTRVGSRRVIQISAGFMIFFSILGKFGALFA

SIPFPIFGAVYCVLQFTNMNSMRNLFITGVAFFLGLS

>Potri.011G068200.1

ALGTAVMIPSFLVPLMGGRVVQTLLFVEGINTLLQTLFGTRLPTVIGGSYAFMVPIISI

IHDRFLSTMRAVQGALIVSSSIQIILGFSPLGMVPVIALVGFGLIGFPMLILFVICSLER

FALLLSITVIWAYAHLLTASGAYKCRTDQAYLISSAPESTGAYKAASRLASATPPPAHVL

SRGQGIGILLDGLFGTMTGSTENIGLLGSTRVGSRRVIQISAGFMIFFSILGKFGALFA

SIPFPIFAAVYCVLQFTNMNSMRNLFITGVAFFLGLS

>Thecc1EG029673t1

ALGTAVMIPSFLVPLMGGRVVQTLLFVEGINTLLQTLFGTRLPTVIGGSYAFMVPIISI

IHDRFLNTMRAVQGALIVASSIQIILGFSPLGMVPVIALVGFGLIGIPMLILFIAFSLER

FGLLISITVIWAYAHLLTASGAYKCRTDKANLISSAPESTGAYKAASRLASATPPPAHVL

SRGQGIGILLDGLFGTLTGSTENVGLLGSTRVGSRRVIQISAGFMIFFSILGKFGALFA

SIPFTIFAAVYCVLQFTNMNSMRNLFITGVALFLGLS

>GSVIVT01021237001

ALGTAVMIPSFLVPLMGGRVVQTLLFVEGINTLLQTLFGTRLPTVVGGSYAFMVPVISI

IHDRFLNTMRAIQGALIVASSIQIILGFSPLGMVPVISLVGFGLIGIPMLFLFIAFSLER

FALLISVTVIWAYAHLLTASGAYRCRTDKANLISSAPESTGAYKAASRLASATPPPAHVL

SRGQGIGILLSGLFGTSTGSTENVGLLGSTRVGSRRVIQISAGFMIFFSILGKFGALFA

SIPFTIFAAVYCVLQFTNMNSMRNLFITGVAFFLGLS

>Solyc02g072500.2.1

ALGTAVMIPSFLVPLMGGRVVQTLLFVEGINTLLQTLFGTRLPTVIGGSWAFVVPIISI

IHDRFLSTMRAIQGALIVASSVQIILGFSPVGMVPVIALAGFGLIGVPMFILFVIFSMER

FALIITITVIWAYAHLLTASGAYRCRTDKAGLISAAPESTGAYKAASRLASATPPPAHVL

SRGQGIGILFSGLFGTATGCTENVGLLGSTRVGSRRVIQISAGFMIFFSILGKFGALFA

SIPFPIFAAVYCVLQFTNMNSMRNLFIAGVSLFLGLS

>PGSC0003DMP400049569

ALGTAVMIPSFLVPLMGGRVVQTLLFVEGINTLLQTLFGTRLPTVIGGSWAFAVPIISI

IHDRFLGTMRAIQGALIVASSVQIILGFSPVGMVPVIALAGFGLIGVPMFILFVIFSMER

FALIITITVIWAYAHLLTASGAYRCRTDKANLISTAPESTGAYKAASRLASATPPPAHVL

SRGQGIGILFSGLFGTATGCTENVGLLGSTRVGSRRVIQISAGFMIFFSILGKFGALFA

SIPFPIFAAVYCVLQFTNMNSMRNLFIAGVSLFLGLS

>NNU_04060RA

ALGTAVMIPTLLVTMIGGRLVQTLLFVGGINTLLQTLFGTRLPTVIGGSYAFLVPIISI

IHDRFLQTMRAIQGALIVASSIQIILGFSPLGMVPVISLVGFGLIGIPMLILFIAFSLER

FALVLSITLIWAYAHLLTASGAYKCRTDRAHLISSAPESTGGYKAASRLASATPPPAHVL

SRG

>NNU_19267RA

ALGTAVMIPTMLVNYIGGRVVQTLLFVGGINTLLQTLFGTRLPTVIGGSHAFLVPIISI

IHDRFIQTMRAIQGALIVASSIQIVLGFSPLGMVPVISLVGFGLIGVPMLLLYIAFSFER

FALIISITMIWVYAHILTASGAYRCRTDKANLISSAPESTGAYKAASRLASATPPPAHVL

SRGQGVGVLLDGLFGTGTGSTENVGLLGATRVGSRRVIQISAGFMIFFSILGKFGALFA

SIPFTIFAAVYCVLQFTNMNSMRNLFITGVALFLGLS

>el__p5_sc00065.V1.gene316

ALGTAVMIPTLLVPLMGGRVVQTLLFVTGINTLLQTLFGTRLPTVVGGSYAFVVPIISI

IHDRFLQTMRAIQGALIVSSSIQIILGLER

FALLISITIIWVYAHILTVSGAYRCRTDRANLISSAPESTGAYKAAARLASATPPPAYVL

SRG

>GSMUA_Achr3P31510_001

ALGTAVMIPTFLVPLMGGRVVQTLLFVTGINTLIQTLFGTRLPTVIGGSYAFMVPIISI

IHDRFLQTMRAIQGALIVSSCIQIILGFSPLGMVPVVSLVGFGLIGVPMLILFVASSLER

FSLLITITIIWVYAHLLTVSGAYKCRTDRANLISSAPESTGAYKAAARLASATPPPAHVL

SRGQGIGILLDGLFGTVTGSTENLGLLGSTRVGSRRVIQISAGFMIFFSIMGKFGALFA

SIPFVIFAAVYCVLQFTNMNSMRNLFITGVSIFLGLS

>GSMUA_Achr7P25220_001

ALGAAVMIPTLLVPLMGGRVVQTLLFVTGINTLLQTLFGTRLPTIVGGSYAFVVPIISI

IHDRFLQTMRAIQGALIVSSCIQIVLGFSPLGMVPVVSLVGFGLIGVPMLILFIAASLER

FSLLLTITIIWVYAHLLTVGGAYKCRTDRADLISSAPESTGAYNAAARLASATPPPAYVL

SRGQGIGVLLDGLFGTATGSTENVGLLGSTRVGSRRVIQISAGFMIFFSILGKFGALFA

SIPFTIFAAVYCVLQFTNMNSMRNLFITGVSIFLGLS

>GSMUA_Achr4P02700_001

ALGTAVMIPTLLVPLMGGRVVQTLLFVTGVNTLLQTLFGTRLPTVVGGSYAFVVPIISI

IHDRFLQTMRAIQGALIVSSSIQIILGFSPLGMVPVVSLVGFGLIGIPMLILFIASSLER

FSLLITITIIWVYAHLLTVGGAYKCRTDRANLISSAPESTGTYKAAARLASATPPPAHVL

SRGQGIGILLDGLFGTVTGSTENVGLLGSTRVGSRRVIQISAGFMIFFSILGKFGALFA

SIPFTIFAAVYCVLQFTNMNSMRNLFIVGVSIFLGLS

>GSMUA_Achr4P15210_001

ALGTAVMIPTLLVHLMGGKVVQTLLFVSGINTLLQTLFGTRLPIVIGGSYAFVVPIISI

IHDRFLQTMRAIQGALIVSSSIQIILGFSPLGMVPVVSLVGFGLIGIPMLILFIASSLER

FSLLITIVIIWIYAYLLTVGGAYKCRTDRANLISSAPESTGAFKAAARLASATPPPAHVL

SRGQGIGILLDGLFGTASGSTENVGLLGSTRVGSRRVTQISACFMIFFSIMGKFGAFFA

SIPFTIFAAVYCVLQFTNMNSMRNLFIVGVSIFLGLS

>LOC_Os01g55500.1

ALGTAVMIPAVLVPMMGGRVVQTLLFVTGINTLLQSLFGTRLPTVIGGSYAFVVPIMAI

IQDRFLQTMRAIQGALIVSSSIQIILGFSPLGMAPVVALLGFGLVGLPMLILFVVLSLER

FSLFICIALVWAYAQILTAGGAYKCRTDRANLISSAPESTASYKAAARLASATPPPAHIL

SRGQGIGILLDGLFGTGTGSTENVGLLGSTRIGSRRVIQISAGFMIFFSMLGKFGALFA

SIPFTIFAAVYCVLQFTNMNSMRNLFIVGVSIFLGLS

>Bradi2g50460.1.p

ALGTAVMIPAVLVPMMGGRVVQTLLFVTGINTLLQSLFGTRLPTVIGGSYAFVVPIMAI

VQDRFLQSMRAIQGALIVSSSIQIILGFSPLGMAPVVALLGFGLVGLPMLILFVVLSLER

FSLFICIALVWAYAQILTSGGAYKCRTDRANLISSAPESTASYSAAARLASATPPPAHIL

SRGQGIGILLSGLFGTGTGSTENVGLLGSTRIGSRRVIQISAGFMIFFSMLGKFGALFA

SIPFTIFAAVYCVLQFTNMNSMRNLFIVGVSIFLGLS

>Seita.5G325700.1.p

ALGTAVMIPAVLVPMMGGRVVQTLLFVTGINTLLQSLFGTRLPTVIGGSYAFVIPIVAI

IQDRFLETMRAIQGALIVSSSIQIILGFSPVGMAPVVALLGFGLVGLPMLILFVVLSLER

FSLFICIALVWAYAQILTSGGAYKCRTDRANLISSAPESTASYKAAARLASATPPPAHIL

SRGQGIGILLDGLFGTGTGSTENVGLLGSTRIGSRRVIQISAGFMIFFSMLGKFGALFA

SIPFTIFAAVYCVLQFTNMNSMRNLFIVGVSIFLGLS

>Sobic.003G303000.1.p

ALGTAVMIPAVLVPMMGGRVVQTLLFVTGINTLLQSLFGTRLPTVIGGSYAFVIPIMAI

IQERFLETMKAIQGALIVSSSIQIILGFSPVGMTPVVALLGFGLIGLPMLILFVVLSLER

FSLFICIALVWAYAQILTSGGAYKCRTDRANLISSAPESTASYKAAARLASATPPPAHIL

SRGQGIGILLDGLFGTGTGSTENVGLLGSTRIGSRRVIQISAGFMIFFSILGKFGALFA

SIPFTIFAAVYCVLQFTNMNSMRNLFIVGVSIFLGLS

>Zm00001d012693_T001

ALGTAVMIPTVLVPMMGGRVVQTLLFVTGINTLLQSLFGTRLPTVIGGSYAFVIPIMAI

IQDRFLETMKAIQGALIVSSSIQIILGFSPVGMTPVVALLGFGLIGLPMLILFVVLSLER

FSLFICVALVWAYAQILTSGGAYKCRTDRANLISSAPESTASYKAAARLASATPPPAHIL

SRGQGIGILLDGLFGTGTGSTENVGLLGSTRIGSRRVIQISAGFMIFFSMLGKFGALFA

SIPFTIFAAVYCVLQFTNMNSMRNLFIVGVSIFLGLS

>el__p5_sc00116.V1.gene254

ALGTAVMIPTLLVPLMGGRVVQTLLFVTGINTLLQTLFGTRLPTVIGGSYAFVVPIISI

IQDRFLQTMRATQGALIISSSIQIILG

CRESTGAYKAAARLASATPPPAHVL

SRGQGVGILLDGLFGTGTGST

>Bradi3g34660.1.p

CLGTAVMIPTFLVPLMGGKVVQTMLFVTGINTLLQTLFGTRLPTIIGGSYAFVIPVISI

IRDRFIMTMRATQGALIISSCIQIVLGFSPLGMVPVVALVGLGLIGLPMLVLFVALSLER

FSLVISIALVWVYAHILTVSGAYKCRTDRANLIASADESTGAFKAAARLASATPPPPYVL

SRGQGIGLLFDGLFGTVAGSTENVGFLGSTRIGSRRVIQISAGFMIFFSILGRFGGLFA

SIPFTIFAAIYCVMQFTNMNSMRSLFIIGISLFLGMS

>Sobic.007G106300.1.p

CLGTAVMIPTLLVPLMGGKVVQTMLLVTGINTMLQTLFGTRLPTVIGGSYAFLIPVISI

ISDRFKMTMRAIQGALIISSCIQIILGFSPLGMVPVIALAGLGLIGLPMLLLFVALSLER

FSVLISIALVWLYAHILTVSGAYRCRTDLANLITTMPESTGAFKAAARLASATPPPPFVL

SRGQGIGLLLDGLFGTASGSTENVGLLGSTRIGSRRVIQISAGFMIFFSILGKFGALFA

SIPFTLFAAIYCVMQFINMNSMRSLFIIGMSLFLGIS

>Zm00001d049324_T001

CLGTAVMIPTLLVPLMGGKVVQTVLFVTGINTMLQTLFGTRLPTVIGGSYAFLVPVMSV

ISDRFKMTMRAIQGALIISSCIQIILGFSPLGMVPVIALVGLGLIGVPMLVLFVALSLER

FSVLITIAVVWLYAHILTVSGAYKCRTDRASLITTMPESTGAFKAAARLASATPPPPFVL

SRGQGIGLLLDGLFGTASGSTENVGLLGSTRIGSRRVIQISAGFMIFFSILGKFGGLFA

SIPFTVFAAIYCVMQFTNMNSMRNLFIIGTSLFLGIS

>Seita.6G132400.1.p

CLGTAVMIPTMLVPLMGGLVVQTVLFVTGINTMLQTLFGTRLPTIIGGSYAFIIPVISI

ISDRFKMTMRAIQGALIISSCIQIILGFSPLGMVPVIALAGLGLIGLPMLVLFVTLSLER

FSVLISIALVWVYAHILTVSGAYKCHTNRANLITTAPESTGAFMAAARLASATPPPPFVL

SRGQGIGLLFDGLFGTVSGSTENVGLLGSTRIGSRRVIQISAGFMIFFSILGRFGALFA

SIPFTLFAAIYCVMQFTNMNSMRNLFIIGISLFLGIS

>Seita.9G069900.1.p

CLGTAVMIPTILVPLMGGQVVQTMLFVTGINTMLQTLFGTRLPTIIGGSYAFTIPIISI

ISDRFKMTMRAIQGALIISSCVQIMLGFSPLGMVPLVALGGLGLIGLPMLILFVALSLER

FSVLISIAVVWAYAHILTVSGAHKCRTDHANLMTTAPESTGAFQAAARLASATPPPPFVL

SRGQGIGLLFNGLFGTVSGSAENVGLIGATRIGSRRVIQISAGFMIFFSILGRFGALFA

SIPFALFAAIYCVMQFTNMNSMRNLFIIGTSLFLGFS

>Sobic.008G019300.1.p

CLGTAVMIPTLLVPLMGGIVVQTVLFVTGINTLLQTLFGTRLPTVIGGSYAFVIPVISI

ISDRFKVAMRAIQGAQIISSCIQIVLGFSPLGMVPVVALVGIGLIGLPMLVLFVALSFER

FSVLISVALVWLYAQILTVSGAYKCRTDHANLITTAPESTAAFQAAARLASATPPPPFVM

SRGQGIGLLLDGLFGTVSGSTENVGLLGSTRIGSRRVVQISAAFMIFFSILGRFGALFA

SIPFTLFAAMYCVMQFTNMNSTRNLFVLGVSLYLGIS

>LOC_Os09g15170.1

SLGTAVMIPTMLVPLMGGRVVQTLLFVTGIKTLLQTLFGTRLPTIIGGSYAFVVPILSI

IRDRFVQTMRAIQGSLIVSSSIQIILGFSPLGMVPVVALVGLGLIGLPMLILFVALSLER

FSLLICVALVWVYAHILTASGAYKCRTDRANLISSALETTGAFMAAARLASATPPPAYVL

SRGQGIGTLLDGLFGTGTGSTENVGLLGSTRVGSRRVIQISAGFMIFFSMLGKFGALFA

SIPFPIFAAIYCVLQFTNMNSMRNLFIVGVSLFLGLS

>Sobic.002G168600.1.p

SLGTAVMIPTLLVPLMGGKVVQTLLFVTGIKTLLQTLFGTRLPTIMGGSYAYVVPILSI

IRDRFLQTMRAIQGSLIVSSSIQIILGFSPLGMVPVVALVGLGLISLPMLILFVALSLER

FSLLMCIALVWVYAHILTASGAYKCRTDRANLISSSQETTGAFKAAARLASATPPPAYVL

SRGQGIGTLLDGLFGTGTGSTENVGLLGSTRVGSRRVIQISAGFMIFFSILGKFGALFA

SIPFTIFAAIYCVMQFTNMNSMRSLFIIGVSLFLGLS

>Zm00001d005590_T001

SLGTAVMIPTLLVPLMGGKVVQTLLFVTGIKTLLQTLFGTRLPTVMGGSYAYVVPILSI

VRDRFLQTMRAVQGSLIVSSSIQIILGFSPLGMVPVVALVGLGLIGLPMLILFVALSFER

FSLLMCITLVWVYAHILTASGAYKCRTDRANLISSSQETTGAFKAAARLASATPPPAYVL

SRGQGIGTLLDGLFGTGTGSTENVGLLGSTRVGSRRVIQISAGFMIFFSILGKFGALFA

SIPFTIFAAIYCVMQFTNMNSMRNLFIIGVSLFLGLS

>Seita.2G174800.1.p

SLGTAVMIPTMLVPLMGGKVVQTLLFVTGIKTLLQTLFGTRLPTIMGGSYAYVVPVLSI

IRDRFLQTMRAIQGSLIVSSSIQIILGFSPLGMVPVIALVGLGLIGLPMLILFVALSLER

FSMLMCIALVWVYAHILTASGAYKCRTDLANLISSSQETTGAFKAAARLASATPPPAYVL

SRGQGIGTLLDGLFGTGTGSTENVGLLGSTRVGSRRVIQISAGFMIFFSMLGKFGALFA

SIPFTIFAAIYCVMQFTNMNSMRNLFIIGVSLFLGLS

>Bradi4g27960.7.p

SLGTAVMIPTLLVTHMGGRVVQTLLFVTGIKTLLQTLFGTRLPTVISGSYAFVIPILSI

INDRFMQTMRAIQGALIVSSSIQIILGFSPLAMVPVVSLVGLGLIGLPMLILFVALSLER

FSMLICIALFWVYAHILTASGAYNCRTDRSNLISSALESTGAFMAAARLASATPPPAYVL

SRGQGIGTLLDGLFGTGTGSTENVGLLGSTRIGSRRVIQISAGFMIFFSILGKFGALFA

SIPFGIFAAIYCVLQFTNMNSMRNLFIVGVSLFLGLS

>el__p5_sc00109.V1.gene239

WVIQTSLFVSGINTLLQSLFGTRLPAVMGGSFAYVIPILYI

MRD

ESTGAYKAASRLAIATPPPAYVL

SRGQEIKLRWNVSMLEGWGRIGIKVQE

LQFTNMNSMRNLIITGLSLFLGIS

>AT2G26510.1

MLGTTVLIANTLVSPMGGRVIQTILFMSGINTLLQTLIGTRLPTVMGVSFAYVLPVLSI

IRDRFRHTMRTVQGSLIISSFVNIIIGFSPIIVVPVVSVVSLGLIGLPMLILLIITQLER

YALLVCLAIIWAFAAILTVSGAYNCRTDRAFLMSSAPESTGVFFAASRLAGATAPPAHVV

SRSQGIGVLLEGIFGSITGNTENVGLLGLTRIGSRRVVQVSTFFMIFFSIFGKFGAFFA

SIPLPIFAGVYCIIQFTDTNSMRNMYVIGVSLFLSLS

>Potri.002G129400.1

MLGTTVLIARNLVPRMGGRVIQTLLFMSGINTLLQTIIGTRLPTVMGPSYAFVLPVLSI

MRDRFVDTMRTIQGSLIVSSFANIILGFSPITVAPVVCVVGLGLIGLPMLILLIICIER

FGLLVCVGIIWAFAAILTVSGAYNCRTDRSYLMSSASESTGTFFAAARLAGATHPPAHVL

SRSQGVSLLLDGIFGAAVGTTENVGLLGLTHVGSRRVVQISTAFMFFFSIFGKFGALFA

SIPLPIFAAIYCVIQFSNNNSMRNHYILGMALFLGIS

>Potri.014G035800.1

MLGTTVLITRTLVTQMGGRVIQTLVFMSGINTLLQTMIGTRLPTVMGPSYAFVLPVLSI

IRDRFVDIMRTIQGSLIVSSFVNIILGFSPITIVPVVCVVGLGLLGLPMLIVLVICLEM

FGLLICIGIIWAFAAILTVAGAYNCRTDRSYLVSSSPESTGTFFAASRLAGATSPPAHVL

SRSQGVGLLLNGIFGAAVGTTENVGLLGLTHIGSRRVVQVSTAFMFFFSIFGKFGALFA

SIPLAIFAAAYCVIQFSNNNSMRNHYVLGLALFLGIS

>NNU_05554RA

MLGTTVMIASFLVPQMGGHVIQTLLFMGGVNTLLQTFLGTRLPTVMGASYSFVIPVMSI

INDRFTHTMRTIQGSLIISSFFNILIGFSPVVVVPVVCVVGLGLIERKTLILLFER

FALLFCIAIVWAFAAILTVSGAYTCRTDRSYLISSAPESTGTYFAAARLAGATPPPAHVL

SRSQGIGMLLDGIFGAAVGTTENVGLLGLTHVGSRRVVQISTAFMIFFSIFGKFGAFFA

SIPFPIFSAIYCVIQFTNNNSMRNLYILGLSLFLGIS

>GSVIVT01009577001

MLGSTVLIASTLVPLMGGRVIQTLLFMAGVNTLLQTLLGARLPTVMGASFAFFIPVMSI

VNDRFVYTMRAIQGSLMVSSIINIFLGFSPVILVPVVCVVGLGLIGLPMLILLVIGQLER

FGLLLCVAIIWAFAGILTVAGAYKCRVDHSYLISSSPESTGTFFAAARLAGATPPPPHVL

SRSQGISLLLDGLFGAVVGTTENVGLLGLTHIGSRRVVQISTAFMFFFSIFGKFGAFFA

SIPLPIFAAIYCVLQFANSNSMRNLYVLGLSLFLGVS

>Thecc1EG033917t2

NLGTTVLIASTIVPRMGGRVIQALLFMSGINTLLQTLIGSRLPTVMGASFAYTLPLLSI

INDRFVRGMRTIQGSLIVSSFVNIILGFSPIVVVPVVCLVGLGLIGLPMLILLVISQLER

FALLLCIGIIWAFAAILTVSGAYNCRTDRSYLMSSAPESTGTFFAAARLSGATAPPAHVL

SRSQGVGMLIEGLFGSLVGTTENVGLLGLTHIGSRRVVQISTGFMIFFSIFGKFGAFFA

SIPLPIFAAIYCVIQFANNNSMRNIYVLGVSLFLGLS

>Solyc04g079430.2.1

MLGTTVMIATVLVPQMGGQVIQSLLFTSGVNTLLQTLFGTRLPTVMGPSFAYIISALSV

INDRFKHTMRAIQGSLIVSSFINIILGFSPVVITPLVCVVGLGLIGLPMLILLVISQLER

FALLLCIGFIWAFAAILTVAGAYNCRIDHSFLLSSAPESTATFYAASRLAGATTPPAHVV

SRSQGIGQLFDGFFGAIVGTTENVGLLGLTRVGSRRVVQISTAFMIFFSIFGKFGAFFA

QIPLPIFAAIYCIIQFANKNSMRNIYVLGVSLFLGIS

>PGSC0003DMP400014150

MLGTTVMIATVLVPQMGGRVIQSLLFTSGVNTLLQTLFGTRLPTVMGPSFAYIISALSV

INDRFEHTMRAIQGSLIVSSIINIILGFSPVVITPLVCVVGLGLIGLPMLILLVISQLER

FALLLCIGLIWAFAAILTVAGAYNCRIDHSFLLSSAPESTATFYAASRLAGATLPPAHVV

SRSQGIGQLFDGFFGAVVGTTENVGLLGLTRVGSRRVVQISTAFMIFFSIFGKFGAFFA

QIPLPIFAAIYCIIQFANKNSMRNIYVLGISLFLGIS

>LOC_Os12g39420.5

MLGSTVMLASTIVPAMGGRVIQSFLFMSGINTLLQTLVGTRLPTVMNASFAFVVPVLSI

AKDRFTHTMRATQGALIVASILNMILGFSPVIMTPVVCVVGLGLIGLPMLILAVVVQFER

YSLLLCIGIVWAFAAILTAAGAYNCRTDKSYLISSAPESTGAHFATARLAGATPPPASVL

SRSQGIGMFLEGIFGAPAGSSENIGLLGLTKVGSRRVIQISTGFMIFFSIFGKFGAFFA

SIPLPIFAAIFCIMQFVNKNSMRNIYIIGLSLFLGIS

>Sobic.008G145800.2.p

MLGSTVMLASVIVPAMGGRVIQSLLFMSGINTLIQTLIGTRLPTVMNASFAFVVPVLSI

ARERFKHTMRTAQGALIVASILNMILGFSPVIMTPVVCVVGLGLIGLPMLILAVLVQFER

YSLLLCIGIVWAFAAILTAAGAYDCRTDKSRLISSAPESTGAHFATARLAGATPPPAHVL

SRSQGIGMFLGGIFSCPTGSSENIGLLGLTKVGSRRVVQISTGFMIFFSLFGKFGAFFA

SIPLPIFAAIYCIMQFANKNSMRNIYIIGLSLFLGIS

>Zm00001d030868_T003

MLGSTVMLATAIVPAMGGRVIQSFLFMSGINTLLQTLIGTRLPTVMNASFAFVVPVLSI

AKERFKHTMRTAQGALIVASILNMILGFSPVIMTPVVCVVGLGLIGLPMLILAVVVQFER

YSLLLCIGIVWAFAAILTAAGAYDCRTDKSFLMSSAPESTGAHFATARLAGATPPPAYVL

SRSQGIGMFLEGIFSVPAGSSENIGLLGLTKVGSRRVIQISTGFMIFFSIFGKFGAFFA

SIPLPIFAAIYCIMQFANKNSMRNIYIIGLSLFLGIS

>Seita.3G369300.1.p

MLGSTVMLASTIVPAMGGRVIQSFLFMSGINTLLQTLVGTRLPTIMNASFAFVVPVLSI

ARERFVHTMRTTQGALIVASILNMILGFSPVIMTPVVCVVGLGLIGLPMLILAVIVQFER

YSLLLCIGIVWAFAAILTAAGAYNCRTDKSFLMSSAPESTGAHFATARLAGATPPPGHVL

SRSQGIGMFLEGIFGAPAGSSGNIGLLGLTKVGSRRVIQISTGFMIFFSIFGKFGAFFA

SIPLPIFAAIYCIMQFANKNSMRNIYIIGLSLFLGIS

>Bradi4g03420.1.p

MLGSTVMLASIIVPAMGGRVIQSFLFMGGINTLLQTLIGTRLPTVMNASFAFVVPVLSI

ARERFVHTIRTAQGALIVASVLNMILGFSPVIMTPVVCVVGLGLIGLPMLILAVVVQFER

YSLLLCITIVWAFAAIITAAGAYNCRTDKSYLMSSAPESTGAHFATARLAGATPPPAHVL

SRSQGIGMFLEGIFAAPCGSSENIGLLGLTKVGSRRVIQISTGFMIFFSIFGKFGAFFA

SIPLPIFAAIYCIGQFVNKNSMRNIYIIGLSLFLGIS

>GSMUA_Achr5P24860_001

MLGSIVMLASFLVPLMGGRTIQTLLFMSGINTLLQTLIGTRLPTVMNSSFAFIIPVMSI

IRDRFVHTTRTIQGALIISSFVNIFIGCSPVTIVPVVCVVGLGLVGLPVLILLVLLVLER

FSIIFCVKIVWAFAAILTAAGAYNCRTDRSYLISSAPESAGAHYAAARLAGATPPP

>Pp3c25_7020V3.1.p

MLGSSIMIPSILVPMMGGRVIQTILFVSGINTLLQTTFGTRLPTIVGGSFAFIIPTITI

INSRFLRTMRAVQGAIIASSTIQIALGLSPVCIAPTIIAAGLGLIGIPHLLLVLIFSFEL

FPVMIGTAITWAYAHLLTMSGAYECRTDRAHIIGSTPESTGHFYVISRLSGATPPPPYVI

SRGEGLGILMDGMFGTAAGSTETIGLIGLTKVGSRRVVQISAGFMICLSILGKFGGIFA

SIPVPMVGAVFCILQFCNMNLQRNIFIIGFSVFMAFS

>73581

MIGTTVLIPSLFVYEMGGRVIQTLLFVNGVMTLVQSFFGTRLPIVMNASFSYVIPIWRI

VNSRFYHTLRAIQGALICASIIQIILGLSPLSVAPVIALVGLGLIGLPEVILLIIISFER

FPVLISAAIIWAYAHLLTVSGAYKCRTDRAHFVKTAPESTATIYGVSRLSNATPPPPFIV

GRSQGIGVLLNGLFGTITGSAENAGLVGLTRVGSRLTVQIAALFMIVLSIFGKFGAIVA

SIPQPIVAAINSVLQFTNLNIIRNLFILGFTLFMGFS

>185282

MIGTTVLIPSMFVFEMGGRVIQTLLFVNGLMTLVQSFFGTRLPVVMNASFSYVIPIWRI

VNSRFYHTMRAIQGALTCASSIQIILGISPLSIAPVIALVGLGLIGLPELLLLIVLSLER

FPVLLSGVIIWAYAHLLTVSGAYRCRTDRAHFVKSAPESTATIYGVSRLANATPPPPFIV

GRSQGIGLMLNGLFGTITGSAENAGLVGLTRVGSRLTVQIAALFMIVLSIFGKFGAIVA

SIPQPIVAAINSVLQFTNLNILRNLFILGFTLFMGFS

>150195

MIGTTVLIPSLLVGNMGARVYQTLLFASGINTLIQTFVGTRLPVVVGGSFAYIIPITSI

TNSRFVHTIRAVQGAVILSSILQIILGISPTTYAPAIILLGLGFIGLPALIILLLFSFER

FPIIVTVIISWAYAYILTVSGAYRCRTDRAHLVGSSPESTAAIYAVSRLANATPPPPFVV

GRGLGFGTLLNGLFGTVVGPTENAGLVGITRVGSRRTVQIAAIFMLVFSILGKFGAVIA

SIPQSIVAAIYCVLQFVNLNITRNLFILGFALFMGFS

>76475

MVGTTVLVTTPLVYAMGGRVIQTLLFASGINTLIQSFLGTRLPAIVGGSYAYILPIFSI

INSRFLHSMKAIQGALICASILQIVLGTSPLTIGPVILMVGIGMIGIPQILLILLFSFER

FAIVIAVALTWAYAHFLTITGAYKCRTDRANLIRSSPESTGSFYGIARLAGATPPPSYVL

SRGQGVGIFINGIFGTAAGPTENAGLVGITRVGSRRTIQVAAFFMIFFSLFGKFGGIFA

SIPAAMVAGIYCVLQFTNLNLPRNLIILGFSVFMAFS

>133065

MLGTTVVIPSIMVDAIGGLVIQALLFVSGLTTLGQTFFGTRLPAVIGGSYAFMIPTLTI

INSRFLQTIRAIQGALICASSIQIALGMCPMTIAPVIMMTALGIIGLPQLALILILSFER

FPIIFSMALIWAYAQVLTLSGAYRCRTDRANLISSAPESTGTFYGLSRLSGATPPPSHVL

SRGQGIGIMLCGMFGTATGCTENAGLIGLTRVGSRRIVQLSAILMIFFSVFGKFGAILA

SIPVPLFAAVYCILQFANLSSRRNLFILGFSLFLGLS

>NNU_19957RA

ILGTSVMIPSFLVPLMGGRVIQTLLFVAGIKTLLQTLFGTRLPVIVGGSYSFVVPVLSI

IGDRFIQTMRAIQGAIIIGSSLQIVLGFSPLSMSVVMCLVGLELIGVPMLLTLVGLYFER

FSILISISLIWAYAFLLTIVGAYKHSTGAYIAASRFAMATPPPAYVL

SRGCGTAWAIPGWKPPSNTNRNYPTPYQVDKCLQMSTFFGKFGALFA

SIPLPIFAAIHCVLQFTNMNKMRNLVITGLSLFLGLS

>Pp3c16_830V3.1.p

NVGVAVLIPLTIIRAIGGRAIQSVLFVSAINTLLQTFFGARLPVVMGNSFYFLPMVLSI

VTKRFLRGMRATQGAFIAGCFLNIILGISPIVIAPVTTLVGLGIIGIPALLIFLLLSFEL

FHVIFGVIIVWIFAVILTVAGAYDCRTDRSGLVSAAPESTGGFYAVSRLAGATPPPPYVI

SRGQGIGLLLNGFWGTFTGTTENVGLVGLTRVGSRRVVQIAAVFMLFFSIFGKFGAVVA

SIPQPIVAAILCLLQFANMNMTRNIFVVGFALFMGLS

>Pp3c16_880V3.1.p

NVGVAVLIPLLIIRSIGGRVIQSVLFVSAINTLLQTFFGSRLPVVMGNSFYFLPMVLSI

VSRRFLRGMRATQGAFIAGCFLNIILGISPIVIAPVTTLVGLGIIGIPALLIFLVFSIEL

FPFIFGVIIVWIFAVILTVAGAYDCRTDRSGLVSAAPESTGGFYAVSRLAGATPPPPYVI

SRGQGIGLLLNGFWGTFTGTTENVGLVGLTRVGSRRVVQIAAVFMLFFSIFGKFGAVVA

SIPQPIVAAILCLLQFANMNMTRNIFVVGFALFMGLS

>Pp3c16_840V3.1.p

MVGTSVLIPLVIIRAIGGRAIQSVLFVSAINTLLQTFFGARLPVVMGNSFYFLPMVLSI

VTRRFLRGMRATQGAFIAGSFLNIILGISPIVIAPVTTLVGLGLIGIPALLIFLVFSFEL

YSITFGVVIVWVFATILTVAGAYDCRTDRSGLVSAAPESTGGFYAVSRLAGATPPPPYVI

SRGQGVGLLLNGFWGTLTGTTENVGLVGLTRVGSRRVVQIAALFMFFFSIFGKFGAVVA

SIPQPIVAAILSLLQFANMNSTRNLFVVGFALFMGFS

>Pp3c5_14260V3.1.p

MLGTSVLIPLTVIRAIGGRTIQSVLFVNAINTLVQTYFGTRLPVVMGSSFYFLPMVLSI

VSRRFLRGMRAAQGGFIAGSALNIILGISPIVIAPVTILVGLGLFGIPALLLFLIFSFEL

YPILIGTVIVWVFASILTAAGAYDCRIDRSGLVSGAPESTGGFYALSRLAGATPPPSHIV

SRGQGIGLLLNGFWGTFTGTTENVGLVGLTRVGSRRVAEISAVFMFFFSIFGKFGAVLA

SIPQPIVAAYLCVLQFANMNLTRNIFVVGFSLFMGLS

>Pp3c6_13450V3.1.p

MLGTSVLIPVTVIRAIGRRTIQSVLFVTAINTLVQTGLGSRLPTVMGSSFYFLPMMLSI

VSRRFLRGMRATQGGFIAGSALNIVLGISPIVIAPITILVGLGIIGIPALLLFLLFS

CLFCYCPESTGEFYSLSRLSGATPPPSYII

SRGQGIGLLFNGFWGTSTGTTENVGLVGLTRVGSRRVAEISAGFMLFFSIFGKFGAVLA

SIPQPIVTAFLCIFQFANMNSTRNIFVVGFSLFMGLS

>Pp3c21_20860V3.1.p

MVGTAVLIPLLIFRADTGGRVIQTVLFVSGINTFIQTTLGTRLPAVMGNSFYFLAPTISI

ITSRFVRSMREVQGAYIAGSALNIILGTSPIVVAPVTALVGLGLVGIPALLVILLFSFER

FPIIVGVTLVWAYAAILTVAGAYDCRTDRSGLVSAAPESTGGFYAISRLAGATPPPPHVI

SRGQGIGVLLNGVFGTFTGATENAGLIGLTRVGSRRVIQISSAFMIFFALFGKFGGIIA

SIPQPIVAAILCVLQFANMNMTRNIFIIGVSIFLGLS

>Solyc04g077720.1.1

ILFLLTLDSLNVTMVLQYLPT

CDR

FAMLFSIAIIWFYAAILTWSGAYKCHVDSSGLIAGSPESTGVFHASARYGSATPVPPSVI

SRGLGIGTMLNAVFGGVTGCAENAGLLAMTRVGSRRVVQISAGFMIFFSILGKFGALFA

SIPASIMAAMYCILQFCNLNSFRTNFILGFSLFLGFS

>PGSC0003DMP400018751

FLLTLDSLNVTMFLQYLPT

CDR

FAMLFSIAIVWFYAAILTWSGAYKCRTDSSGLIAGSPESTGVFHASARYGSATPVPPSVI

SRGENAGLLAMTRVGSRRVVQISAGFMIFFSILGKFGALFA

SIPASIMAAMYCILQFCNLNSFRTNFILGFSLFLGFS

>Cre17.g716800.t1.2

MLGSTVVIPALLVPAMGGRVVQTIFFVSGINTLLQTTIGDRLPIIQGGSFSFLRPAFSI

IAIRFVYTMRELQGSIMGSGLLVMAVGVSPVVVAPTVCMVGLSLQGLMAIVAVILFSFEL

FPLLWSIVVCWAVAAILTTSGAYDCRTDHLEALAAAPESTGDYYACARMCGAPVPPPYVI

SRGEGLGCFMCGLFGTGNGTTENIGAIGLTGVGSRRVVQAGAGIMLLLAVLGKFGALFA

SLPGAVVAGLFCCLQFTDQNSSRNLMIVGFAIYMALS

>AT2G27810.1

MLGSLILVPLVIVPAMGGNVVSTVLFVSGITTLLHTSFGSRLPLIQGPSFVFLAPALAI

INSNFKHIMRELQGAIIIGSAFQAVLGVNPVVVAPTVAAVGLSFIGVVQILLVIIFAFLI

YAVPLSLAITWAAAFLLTETGAYTCRVDTSHALSSAPDSVGSYHASSLLVASRPPTRGVV

SRAEGFTSVLAGLWGTGTGSTENVHTIAVTKMGSRRVVELGACVLVIFSLVGKVGGFLA

SIPQVMVASLLCFLRYSEAGSSRNIIIVGLSLFFSLS

>Potri.004G187900.1

ILGSLILIPLVIVPAMGGMVVSTVLFVSGVTTLLHTSFGSRLPLIQGPSFVYLAPALAI

INSNFKHIMKELQGAIIIASAFQTILGINPVVVAPTLAAVGLSFIGVVQILLVIMFSFLI

YAVPLGLAITWAAAFLLTEAGVYSCRVDTSYALKSSPDSVGSYHASSLLAASGPPTPGVV

SRGEGLCSVLAGLWGTGTGSTENVHTIAVTKMGSRRAVELGACALILLSLIGKVGGFIA

SIPEVMVAALLCFLRYSEAGSSRNIIIVGLSLFFSLS

>Potri.009G148600.1

MLGSLILIPLVVVPAMGGTVVSTVLFVSGVTTLLHTSFGSRLPLIQGPSFVYLAPALAI

INSNFKHIMKELQGAIIIASAFQTILGINPVVVAPTIAAVGLSFIGVVQILLVIMFSFLI

YAVPLGLAITWAAAFLLTEAGVYSCRVDTSHALKSSPDSVGSYHASSLLAASRPPTPGVV

SRGEGLCSVLAGLWGTGTGSTENVHTIAVTKMGSRRAVELGACALILLSLIGKVGGFIA

SIPEVMVAALLCFLRYSEAGSSRNIIIVGLSLFFSLS

>Thecc1EG041503t1

MLGSLILIPLVIVPPMGGNVVSTVLFVSGVTTLLHSFFGSRLPLIQGPSFVFLAPALAI

INSNFKHIMKELQGAIIIASAFQTILGINPVVVAPTIAAVGLSFIGAAQILLVIIFSFLI

YAVPLGLGITWAAAFLLTEAGAYSCRVDTSHALKSSPDSVGSYHASSLLVASRPPTPGVV

SRGEGLSSVLAGLWGTGTGSTENVHTIAVTKMGSRRAVELGACVLIVLSLVGKVGGFIA

SIPEVMVAALLCFLRYSEAGSSRNIIIVGLSLFFSLS

>GSVIVT01031396001

ILGSLILIPLVIVPAMGGMVVSTVLFVSGVTTLLHTSFGTRLPLIQGPSFVYLAPALAI

INSNFKHIMKELQGAVIIASAFQTILGINPVVVSPTIAAVGLSFIGAVQILLVIIFSFLI

YAVPLGLAITWATAFLLTEAGVYNCRVDTSHALKSSPDSVGSYHASSLLVASRPPTPGVL

SRGEGISSVLAGLWGTGTGSTENVHTIAVTKMGSRRAVEFGACVLIALSLVGKVGGFIA

SIPEVMVAALLCFLRYSEAGSSRNIIIVGLSLFFSLS

>NNU_01066RA

ILGSLILIPLVIVPAMGGMVVSTVMFVSGVTTLLHTSFGSRLPLIQGPSFVYLAPALSI

INSNFRHIMKQLQGAIIISSAFQALLGINPVVVSPTVAAVGLSFIGAVQILLVVIFSFLI

YAVPLGLAITWAAAFLLTEAGAYNCRVDTSHALKSSPDSVGSYHASSLLVASRPPTPGVL

SRGEGLSSVLAGLWGTGTGSTENVHTIAVTKMGSRRAVELGACILILLSLVGKIGGFIA

SIPDVMVAALLCFLRYSEAGSSRNIIIVGLSLFFSLS

>NNU_25300RA

ILGSLILIPLVIIPAMGGMVVSTVMFVSGVTTLLHTFFGSRLPLIQGPSFVYLAPALVI

INSNFKHIMKHLQGAIIISSAFQAFLGINPVVVSPTIAAVGLSFIGIVQILLVVIFSFLI

YAVPLGLAITWAAAFLLTETGAYNCRVDTSHALKSSPDSVGSYHASSLLVASRPPTPGVL

SRGEGLSSVLAGLWGTGTGCTENVHTIAVTKMGSRRAVELGACILILLSLVGKVGGFIA

SIPDVIVAALLCFLRYSEAGSSRNIIIVGLSLFFSLS

>Solyc11g066900.1.1

ILGSLILIPLVIVPAMGGNVVSTVLLVSGVTTLLHTSFGSRLPLIQGASFVYLAPALAI

INSKFKHIMKELQGALIISSAFQAILGINPVVVAPTVAAVGLSFIGAMQILLVILFSFLI

YAVPLGLAITWSVAFLLTAVGVYSCRVDALHALGSAPDSIGSYHASSLLVASRPPTPGVL

SRGEGLCSLLAGLWGTGTGSAENVHTIAVTKMGSRRAIELGACVLIVLSLIGKVGGFIA

SIPDVIVAGLLCFLRYSEAGSSRNIIIVGLSLFLSLS

>PGSC0003DMP400046114

ILGSLILIPLVIVPAMGGNVVSTVLLMSGVTTLLHTSFGSRLPLIQGPSFVYLAPALAI

INSKFKHIMKELQGALIISSAFQAILGINPVVVAPTVAAVGLSFIGAVQILLVIFFSFLI

YAVPLGLAITWAVAFLLTAVGVYSCRVDALHALGSAPDSVGSYHASSLLVASRPPTPGVL

SRGEGLCSLLAGLWGTGTGSAENVHTIAVTKMGSRRAIELGACVLIVLSLIGKVGGFIA

SIPDVIVAGLLCFLRYSEAGSSRNIIIVGLSLFLSLS

>GSMUA_Achr10P10180_001

IIGSLILIPLVLVPAMGGALVSTVLFVSGVTTLLHTLFGTRLPLIQGPSFVYLAPALAI

INSNFKHIMKELQGAIIISSAFQAIMGINPVVVSPTIAAVGLSFIGMMQILLVIIFSFLI

YAVPLGLGVTWAIAFLLTESGVYNCRTDTSHAIKSSPDSVGTYHASSLLVASRPPTAGVL

SRGEGISSILAGLWGTGVGSTENVHTIAVTKMGSRRAVELGAVILILLSFVGKVGGFIA

SIPDVMVAGLLCFLRYSETGSSRNSIIVGLSLFFSLS

>GSMUA_Achr3P22820_001

IMGSLILIPLVIVPAMGGAVVSTVFFISGVTTLLHTFFGTRLPLIQGPSFVYLAPALAI

INSNFKHIMKELQGALIISSAFQAIMGINPVVVSPTIAAVGLSFIGMVQILLVVVFSFLI

YAVPLGLGITWAIAFLLTASGVYSCRVDTSHALRSSPDSVGTYHASSLLVASRPPTAGVL

SRGEGISSIFAGLWGTGVGSTENVHTIAVTKMGSRRAVELGAVMLILLSFVGKVGGFIA

SIPDVMVAGLLCFLRYSETGSSRNNIIVGLSLFFSLS

>LOC_Os01g63870.1

MKHLQGAIIIGGAFQVLLGINPVVISPTVAAVGLSFMGLLQLLIVVMFAFLI

YAVPLALGITWAIAFVLTATGVYSCRVDTSHALRSSPDSVGSYHASSLFVATRPPTAGVV

SRGEGVSTVLAGLWGTGVGSAENVHTIAVTKMGNRRAVGFGAIVLILLSFVGKVGAFIA

SIPDVLVAALLCFLRYSAKGSSRNSIVVGLALFLSLS

>Sobic.003G362200.2.p

MVGSIILIPLVMVPAMGGAVVSTVLLVTGMTTLLHMFVGTRLPLVQGPSFVYLAPALAI

INSNFKHIMKHLQGAIIIGGAFQVVLGINPVVVSPTVAAVGLSFMGILQLLMVVIFAFLI

YAVPLGLGITWAVAFVLTATGVYSCRVDTSHALRSSPDSVGSYHASSLFVATRPPTSGVV

SRGEGVSTVLAGLWGTGVGSAENVHTIAVTKMGSRRAVGFGAILLVLLSIIGKVGAFIA

SIPDVMVAALLCFLRYSATGSSRNSIIVGLALFLSLS

>Seita.5G388000.1.p

MVGSIILIPLVMIPAMGGAVVSTVLLVTGMTTLLHMFVGTRLPLVQGPSFVYLAPALAI

INSNFKHIMKHLQGAIIIGGAFQVVLGINPVVVSPTVAAVGLSFMGILQLLMVVIFAFLI

YAVPLGLGITWAIAFVLTATGVYSCRVDTSHALRSSPDSVGSYHASSLFVATRPPTSGVV

SRGEGVSTILAGLWGTGVGSAENVHTIAVTKMGSRRAVGFGAILLLLLSIVGKVGAFIA

SIPDVMVAALLCFLRYSATGSSRNSIIVGLSLFLSLS

>Zm00001d012307_T002

MVGSIILIPLVMVPAMGGAVVSTVLLVTGVTTLLHMFVGTRLPLVQGPSFVYLAPALAI

INSNFKHIMKHLQGAIIIGGAFQVFLGINPVVVSPTVAAVGLSFMGILQLLMVVIFAFLI

YAVPLGLGITWAVAFVLTATGVYSCRVDTSHALRSSPDSVGSYHASSLFVATRPPTSGVV

SRGEGVSTVLAGLWGTGVGSAENVHTIAVTKMGSRRAVGFGAILLILLSIVGKVGAFIA

SIPDVMVAALLCFLRYSATGSSRNSIIVGLALFLSLS

>Bradi2g55440.1.p

MLGSIILVPLVIVPAMGGAVVSTVLLVSGLTTLLHTLFGTRLPLVQGPSFVYLAPALAI

INSNFKHIMKHLQGAIIIGGVFQVLLGINPVVVSPTVAAVGLSFIGVLQLMMVIIFAFLI

YAVPLGLGITWAIAFVLTATGVYSCRVDTSHVLRASPDSVGSYHASSLFVATRPPTAGII

SRGEGVSTVLAGLWGTGVGSAENVHTIAVTKMGSRKAVSFGAIVLLLLSLIGKFGAFIA

SIPDVMVAALLCFLRYSATGSSRNSIVVGLALFLSLS

>Zm00001d007116_T001

I

QVGSYHASSLFVATRPPTYGVV

SRGEGVSTVLAGLWGTGVGSAENVHTIAVTKMGSRRAVGFGAILLVLLSIVGKVGAFIA

SIPDVMVAALLCFLHYSATGSSRNSIIVGLALFLSLS

>Pp3c13_10930V3.1.p

IAGSLILIPLVIVPAMGGSVVSSMLMVSGLSTLLHTSFGSRLPLIQGASFVHLAPALAI

IFSRFKKTMRELQGAVIIGGAFQTFLGINPVVVAPTVASVGLAFIGIPQILALVLFAFQV

YAVPLGLALTWAYAFLLTESKVYNCRTDVSNALSTSADSVGAYHASSLLVASRAPTHGVV

SRSEGLTSVLAGFWGTGSAATENVHTIAVTKMGSRRAVEFGACVLIVASVIGKISGFIA

TIPQVIVAGLLVFLRYSETGSSRNVLIVGLSLFLSFS

>Pp3c3_8470V3.1.p

IVGSLLLIPLIIVPAMGGKVVSSVLMVSGISTLLHTSFGSRLPLIQGASFVYLAPALAI

IFSRFKKTMRELQGAIIIGSAFQALLGINPVVVAPTLAAVGLAFIGIPQILLLVLFAFQV

YAVPLGLALTWAFAFLLTESKVYTCRTDVSNALSTSADSVGAYHASSLLVASRAPTPGVV

SRSEGLTSILAGIWGIGTGATENVHTIAVTKMGSRRPVEFGACILIAASLIGKISGFIA

SIPQVIVAGLLVFLRYSETGSSRNVLIVGLSLFLSLS

>Pp3c3_22050V3.1.p

IIGSLILIPLVIVPAMGGKVISSMFMVSGISTLLHCLFGTRLPLVQGASFVYLGPTLAI

VFSRFKSTMRELQGAIIISSLFQTLLGINPVVVAPTVTAVGLAFIGIPQFVVVLFLAFQV

YAVPLGLAAVWAYAFLLTESKVYTCRTDASDALSSTSDSVGSYHAASLLVASRAPTPGVV

SRGEGVTSFLAGLWGTGAGATENVHTIAVTKMGSRRAVEFGACVMIGISLVGKISGFIA

SIPQAVAGGLLVFLRYSETGSSRNVLIVGLSLFLSLS

>81830

IIGSLILVPLVLVPLIGGRVVSTTLLVSGITTLIHLCFGSRLPLIQGPSFVYLAPALVI

ANSRFKHTMKELQGAVIISSLFQIIAGINPVIVSPTVAAVGLAFIGIPQIIVVIIFAFQI

YAVPLGLATTWAYAFLLTETGAYNCRTDASHALRDAADSVGSYHATSLLVASRAPTPGLV

SRAEGITSALAGLWGIGTGATENVHTIAVTRMGSRHAVTFGAFVLIALSFIGKVGAFLA

SIPQVMVAALLCILRYTETGSSRNVLIVGLSLFLSLS

>268297

IVGSLVLTPLIFVPAMGGKVVSTMLLVTGITTLLHSFFGSRLPLVQGASFAYLAPILTI

IHSRFKHIMRELQGAVIISSVFQMVVGINPVVVAPTVAAIGLAFIGLPQLLIVLFFAFQV

YAVPLGLGIIWAYAFLLTETGAYNCRTDVSTALKDAADSVGTYHATSLLVASRAPSPGVV

SRSEGITSALAGIFGTGAGATENVHTIAVTKMGSRRAVEFGACVLIILALVGKVGAFIA

SIPTVIVAGLLAFLRYSETGSSRNVLIVGLSLFTALS

>AT4G38050.1

LVGSLVFIPLVIVPAMDGSVISTMLLLTGVTTILHCYFGTRLPLVQGSSFVYLAPVLVV

INSKFRDTMRELQGAIIVGSLFQCILGINPVVVAPTVAAVGLAFISVPLILLLLIFTFRI

YAVPLSALLIWTYAFFLTVGGAYDCRTDASNAWRTASDSVGTYHSASMIVNAKRPTRGIV

SRGEGFCSLLAGIWGSGTGSTENIHTINITKVASRRALVIGAMFLIVLSFLGKLGAILA

SIPQALAASVLCFLRYTQTASFRNITIVGVSLFLGLS

>Potri.007G011400.1

MAGSLIFIPLIIVPAMGGEVISTMLLISGITTILHSYFGTRLPLVQGSSFVYLAPALVI

INAKFRHIMRELQGAIIVGSLFQTILGINPVVVAPTVAAVGLAFISIPLILLVLIFTFQI

YAVPLSVLMIWTYAFFLTAGGAYNCRTDASNAWRTAADSVGTYHSTSLLVNSKPPTPRIV

SRGEGFCSVLAGIWGCGTGSTENVHTVNITKVASRRVVEVGAAFLILFSFIGKVGAILA

SIPQALAASILCFLQYSQTASFRNITIVGVSLFLGLT

>GSVIVT01007130001

LAGSIIFIPLVIVPAMGGTVISTMLLVTGITTILQSYFGTRLPLVQGSSFVYLAPALVI

INSKFRHIMRELQGAIIVGSIFQSILGINPVVVAPTIAGVGLAFISIPQILLVLIFTFRI

YAVPLSILIIWAYAFFLTAGGAYNCRTDVSNAWRTAADSVGTYHSTSLLVNSKPPTPGIV

SRG

>Thecc1EG000977t1

LAGSLIFIPLVMVPAMGGTVISTMLLVSGITTILHSYFGTRLPLVQGSSFIYLAPALVI

INAKFRHIMRELQGAIIIGSVFQSILGINPVVVAPTVAAVGLAFVSVPLILLVLICTFRI

YAVPLSVMITWIYAFFLTTGGAYDCRTDVSNAWRNAADSVGTYHSASLLVSSKPPTPAVV

SRGEGFCSMLAGLWGSGTGSTENTHTINITKMASRRAVVFGALFLILFSFVAKVGAILA

SIPLSLAASILCFLQYTQTTSFRNITIVGVSLFLGLS

>NNU_05186RA

LAGSLIFIPLVIVPAMGGTVISTMLLISGITTILHSYFGTRLPLVQGSSFVYLAPVLVI

MNSKFKDIMKELQGAIIVGAIFQSVLGINPIVVAPTIATVGLAFISIPQILLLLIFTFQV

YAVPLSVAVVWAYAFFLTDGGAYDCRTDASSAWRTAADSVGTYHSASLLVISRPPTPGIV

SRGEGFCSILAGLWGTGSGSTENVHTIDITKVASRRAVELGALLLILFSFFGKVGALLA

SIPQALAVSVLCFLQYGQTSTFRNITIVGLSLFFSLS

>NNU_12861RA

LAGSLIFVPLVIVPAMGGTVISTMLLVSGITTILHSYFGSRLPLVQGSSFVYLAPALVI

MNSKFKHIMKELQGAIIVGAIFQSILGINPIVVAPTIAAVGLAFISIPQILLLLIFTFRV

YAVPLSVTIVWAYAFFLTAGGAYDCRTDASNAWRTADDSVGTYHSASLLVNSRPPTPGVV

SRGEGFCSILAGLWGTGSGSTENAHTIDITKVANRRAVELGAVMLILFSFMGKVGALLA

SIPQALAVSVLCFLQYGQTASFRNITIVGVSLFFSLS

>LOC_Os07g30810.1

IAGSLVFVPLILVPTMGGTVISTILLVSGLTTILHTFFGSRLPLIQGSSFVYLAPALVI

SNSKFKHIMRELQGAILVGSVFQIILGINPVVVAPTIAAVGLAFISMPLILLVLLCTFLI

YAVPFSVAVVWAYAFFLTAGGAYNCRTDASNAWRTAADSLSSYHATSLLVNLSPPTRGVV

SRGEGISTLIAGVWGTGTGSTENIHTLENTKMASRRALQFGAVLLVIFSFFGKIGALLA

SIPVALAASVLCFLRYTQAASSRNMIIVGFTLFISMS

>Sobic.002G306300.1.p

IAGSLVFVPLILVPTMGGTVISTMLLVSGLTTILHTFLGSRLPLIQGSSFVYLAPALVI

ANSKFKHIMRELQGAILVGSVFQIILGINPVVVAPTIAAVGLAFISLPLILLVLLCTFLV

YAVPLSVAIVWAYAFFLTAGGAYNCRTDVSTAWKTAADSLSSYHAASLLVNLSPPTRGVV

SRGEGISTFIAGVWGTGTGSTENIHTLETTKMGSRRALQLGAAVLVIFSFFGKIGALLA

SIPLALAASVLCFLRYTQAASSRNMIIVGFTLFISLS

>Seita.2G318900.1.p

IAGSLVFVPLILVPTMGGTVISTMLLVSGLTTILHTFLGSRLPLVQGSSFVYLAPALVI

ANSKFKHVMRELQGAILIGSVFQILLGINPVVVAPTIAAVGLAFISLPLILLVLLCTFLV

YAVPLSVAIVWAYAFFLTAGGAYNCRTDVSTAWKTAADSLSSYHAASLLVNLSPPTRGVV

SRGEGISTFIAGVWGTGTGSTENIHTLETTKMASRRALQLGAALLVVFSFFGKIGALLA

SIPVALAASVLCFLRYTQAASSRNMIIVGFTLFISLS

>Zm00001d021519_T001

IAGSLVFGPLILVPTMGGTVISTMLLVSGLTTILHTFLGSRLPLIQGSSFVYLAPALVI

ANSKFKHIMRELQGAILVGSVFQIILGINPVVVAPTIAAVGLAFISMPLILLVLLCTFLV

YAVPLSVAIVWAYAFFLTAGGAYNCRTDVSTAWKTAADSLSSYHAASLLVNLSPPTRGVV

SRAEGVSTFIAGVWGTGTGSTENIHTLETTKMGSRRALQLGAAVLVIFSFFGKIGALLA

SIPLALAASVLCFLRYTQAASSRNMIIVGFTLFISLS

>Zm00001d006408_T001

IAGSLVFVPLILVPTMGG

KFKHIMRELQGAILVGSVFQIILGINPVVVAPTIAAVGLAFISMPLILLVLLCTFLV

YAVPLSVAIVWAYSFFLTAGGAYNCRTDVSSAWRTAADSLSSYHAASLLVNLSPPTRGVV

SRAEGISSFIAGVWGTGTGSIENIHTLETTKMASRRALQLGAAVLVVCSFFGKIGALLA

SIPLALAASVLCFLRYTQAASSRNLIIVGFTLFISLS

>Bradi1g27197.1.p

IAGSLVFIPLILVPTMGGTVISTMLLVSGLTTILHTFLGSRLPLIQGSSFVYLAPALVI

ANSKFKHIMRELQGAILVGSVFQIILGINPVVVAPTIAAVGLAFISMPLIVLLLLCTFLI

YAVPLSVGITWAYAFFLTAGGAYNCRTDVSSAWRTADDSLSSYHAASLVVNLSPPTRGVV

SRGEGISSFIAGLWGTGTGSTENIHTLDITKMASRRALQLGAALLVIFSFFGKIGALLA

SIPVALAASVLCFLRYTEAASSRNMIIVGFSLFISLS

>Zm00001d022079_T001

IAGSLVFVPLILVPAMDGTVISTMLLVSGLTTILHTFLGSRLPLIQGSSFVYLAPALVI

ANSKFKHIMRELQGAILVGSVFQIILGINPVVVAPTIAVVGLAFISMPLILLVLLCTFLV

YAVPLSVAIVWAYAFFL

>GSMUA_Achr5P15470_001

LAGSLVFIPLIMVPTMGGTVISTMLLVSGITTIMHSYFGSRLPLVQGSSFVYLAPALVI

INSKFKHIMRELQGAILVGSVFQAVLGINPVVVAPTVAVVGLAFISIPLILLVLIFTFLI

YAVPLSVAIVWAYAFFLTAGGAYNCRTDVSNAWRAAADSVGTYHATSLLVNLSPPTPGVV

SRGEGFASILAGLWGTGTGSTENIHTIDVTRVASRKALEFGASLLILFSFVGKIGALLA

SIPLALAASVLCFLQYTQTASSRNIIIVGFTLFISFS

>Zm00001d024918_T001

IAGSLVFVPLILVPAMGGTVISTMLLVSGLTTILHTFLGSRLPLIQGSSFVYLAPALVI

ANSKFKHIMRELQGAILVGSVFQIILG

ADFLKL

VGKIGALLA

SIPLALAASVLCFLRYTQAASSRNMIIVGFTLFISLS

>Zm00001d042322_T001

LLVSGLTTILHTFLGSRLPLIQGSSFVYLAPALVI

ANSKFKHIMRELQGAILVGSVFQIILG

ADFLKL

VGKIGALLA

SIPLALAASVLCFLRYTQAASSRNMIIVGFTLFISLS

>Solyc11g010690.1.1

LAGSLIFIPLITVPTMGGDVVSTVLLLSGLTTILHSYFGTRLPLVQGSSFVYLAPALVI

MNSFRI

YAVPVSVMIIWAYAFFLTAGGAYNCRTDVSNAMRTAADSIGSYHSAAIRINLKPPTPGIV

SRGEGFCSVLAGLWGTGTGATENVHTINATKVANRRAVQLGAVLLILFSFVGKIGAILA

SIPQALAAAVLCFLQYTQNASSRNIIIVGVSLFFGLS

>PGSC0003DMP400028295

LAGSLIFIPLITVPTMGGDVVSTMLLVSGLTTILHSYFGTRLPLVQGSSFVYLAPALVI

MNSKFRYIMRELQGAIIVGSIFQSFLGVNPVVVAPTVAAVGLAFISLPQILLILIFTFRI

YAVPVSVMIIWAYAFFLTAGGAYNCRTDVSNAMRTAADSIGNYHSAAIRINLKPPTPGIV

SRGEGFCSVLAGLWGTGTGATENLHTINATKVANRRAVQLGAALLILFSFVGKIGAILA

SIPQALAAAVLCFLQYTQNASSRNIIIVGVSLFFGLS

>Zm00001d053955_T001

IAGSLVFVPLILVPAMGGTVISTMLLVSGLTTILHTFLGSRLPLIQGSSFVYLAPALVI

ANSKFKHIMRELQGAILVGSVFQIILGINPVVVAPTIAAVGLAFISMPLILLVLLCT

VPLSVAIVWAYAFFLTAGGAYNCRTDVSTAWKTAADSLSSYHAASLL

>Zm00001d053604_T002

KHIMRELQGAILVGSVFQIILGINPVVVALTIAAVGLAFISMPFILLVLLCTFLV

YALAD

VEFADFLKLSSLPGKIGALLA

SIPLALAASVLCFLRYTQAASSRNLIIVGFTLFISL

>Pp3c12_11660V3.1.p

MVGGIITAPLLIAMLGFYLVSAALIVAAVASFVQIVQLRIPGTMGVSFSFVPISQQ

VISTPEAAYGKVLGTVLVCCFYQIFVSFPPVVTGTCLVLLGSALLGLVVFFAIIFAE

IVGLLVGMIVASSVHYSLRFVTGQQISDAKECIGDITATAEASQLEPVGEKFE

KSIDGINSFFSALATITPVTTQNNGVLSLSRCASRYAGYACCVWLLILGIVGKFAAIIL

TIPNCVLGGMTTFLNLREGLNRRNRFIAIMSIGVGMG

>Pp3c3_22020V3.1.p

MVGGIISPPLLISMTGFYLVSAALIVAAIASFIQVMQIKIPGTMGVSFSFVPVAQQ

VITTPEEAYGKVLGTLLVCCFFQMAISFPPVVTGTCLVLLGAALLGLVVFFSIIFAE

VVGLLVGMIVASNVHYTKRYVTGKQIADAKECIGDITATTEASHLEPVGEKFE

QRIDGMNSFLGALGTITPVTTQNNGVLSLSRCASRYAGYACCGWLFVIGIVGKFAAIIL

TIPNCVLGGMTTFLNFGEGLNRRNRFIAIMAIGVGMG

>Cre10.g433200.t1.2

MVGGLITPPLLISNLGFGTYLVQASLIVCGIMTFFQVLGVRIWKTMGVSFTTVPIATS

VIGTFEEAYGMFLGTIAMCAVIPALLSFPPIVCGITIMMIGVHLLGFLVFVTIIVLE

ILALLFGYFIAAVTNYQKKYVITTKIDQAPETVGDTAATLEASRMQVNTEDGT

RRIDGISGIFSALATSLPLTTQNNGVISLTNVAARQAGFAAAFWLLLLGILGKVGAWIT

TIPECVLGGMTTFIINGDPLTRRSRFILACSLALAFG

>Cre10.g433250.t1.2

MVGGLITPPLLISNLGFGTYLVQASLIVCGIMTFFQVLGVRIWKTMGVSFTTVPIATS

VIGTFEEAYGMFLGTIAMCAVIPALLSFPPIVCGITIMMIGVHLLGFLVFVTIIVLE

ILALLFGYFIAAVTNYQKKYVITTKIDQAPETVGDTAATLEASRMQVNTEDGT

RRIDGISGIFSALATSLPLTTQNNGVISLTNVAARQAGFAAAFWLLLLGILGKVGAWIT

TIPECVLGGMTTFIINGDPLTRRSRFILACSLALAFG

>Cre10.g442800.t1.1

MVGGLITPPLLISNLGFGTYLVQASLIVTGIMTFFQVLGVRIWKTMGVSFTTVPIATS

VIGTFEEAYGMFLGTIVMCAIIPVILSFPPIVCGITIMMIGVHLLGFLVFVTIIFLE

ILALLFGYLIAAVTTYQKKYVITTKIDQAPETVGDTAATMEASRMLVNTEDGT

RRIDGISGIFSALATSLPLTTQNNGVISLTNVAARQAGFAAAFWLFLLGVLGKVGAWIT

TIPECVLGGMTTFIINGDPLTRRSRFILACSLALAFG

>Cre10.g442600.t1.2

MVGGLITPPLLVSTLAFGNYLVQAALIVCGIMTFFQVLGVRIYKTMGISFTTVPIATS

VIGTFEEAYGNFLGTIAMCGVIPVILSFPPLVCGIVIMMIGVHLLGFLVFITIIFLE

ILALLFGYLIAAVTTYQKKYVITTKIDQAPETVGDTSATMEASRMAVDTEDGT

RRIDGISGIFSALATSLPLTTQNNGVIALTNVAARQAGFAAAFWLFLLGILGKVGAWIT

TIPECVLGGMTTFIINGDPLTRRSRFILACSLALAFG

>CDF38528

MIGGVIAVPRILSGGGYLISTALVVSGLASWIQIVRIKLVSGVSFTFLPIAQT

MFSRDAYGMWLGTIMVGALLEIGLSFPPLVTGVTVFLIGASLLGFFVFTIIIVLE

MIGLIAGIILAASLGYMDIIDAAPETLGDIAASSEASRVATQGPEFE

SRIDGLNSLLAGLLTSSPTTTQNNGVIVMTRTANTTAGLWAAGWLILAGIIGKVGGVFV

ALPDAALGGMTTFLQWDRRERFILAISLSL

>Pp3s46_310V3.1.p

MFGSTVLVPILTGLATALFTSGIGTLLFLLILGSSFAFIGPIIAV

TASGTALLGCFLSGLVYLPPVVIASVIVVIGLSLISLVTTIVTILAALGL

IPILIGIIVGYVYTLLPDLIDLQKIADAPEHLGHLLVTSKVM

DRDDGIATSLAAFIGGPPATTENIGVLAITRVFSKMVIGLAAVLAILFAFIGKISTLLM

SIPTPVLGGVSIIIDFSNKRNMVLAAAILVTGIG

>AT3G10960.1

IMGLMANLPLALAPGMGTRTALAAVFIEGLIFLFISAIGFRAKLAIGLFLAFI

LQNNQGIGLVGYSPSTLVTLAPVITLGIVGFVIIAY

YGIVFVTAVSWFRNTEVTAFKKIVDVHVIKHTADTTGTLYSMARFAGFV

DEDASAIVIGSLLGTSPVTVESSTGIREGGRTGLTAITVAVYFLLAMFFTPLLA

SIPAWAVGPPLILIDWEDMREAIPAFVTMILMPLTYS

>Potri.013G083300.1

IMGAFANLPLALAPGMGTKSALAAVFIEGVIFLGISAIGLRAKLAIGLFLAFI

LQNGQGIGLVGYSSSTLVTLAPVMTLGVVGFVIIAY

YGIVFVTAISWFRDTKVTVFKKVVDVHVIESTADTTGTLYSMARFAGFS

DQDASSIVVGSLLGTSPVTAESSTGIREGGRTGLTALTVAGYFFLAFFFTPLLA

SIPAWAVGPPLILVEWNDMRQAIPAFMTLILMPLTYS

>Thecc1EG045453t1

IMGAFANLPLALAPGMGTKSALAAVFIEGLIFLFISAVGFRAKLAIGLFLAFI

LQNNQGIGLVGYNPSTLVTVAPVLTLGIVGFVIIAY

YGIVFVTAVSWFRHTKVTAFKKVVDIHLIESTADTTGTLYSMARFAGFT

DEDAMSIVVGSLLGTSPVTAESSTGIREGGRTGLTALTVAGYFFLAFFFTPLLA

SIPAWAVGPPLILIEWDDMRQAIPAFVTLILMPLTYS

>Thecc1EG019735t1

IMGIFANLPLALAPGMGTKSALTAIFIEGLLFLLISAVGLRAKLAIGLFLAFI

LQNNQGIGLVGYSSSTLVTLAPVVTLGIVGFVIIAY

YGIIFVTVISWFRNTAVTAFKKVVDVHVIKRTADTTGTLYSMARFAGFT

DEDAASIVVGSLLGTSPVTAESSTGIREGGRTGLTALTVAGYFFLAFFFTPLLA

SIPAWAVGPPLILVEWDDMRQAIPAFVTLILMPLTYS

>NNU_12259RA

IMGAFANLPLALAPGMGAESALAAVFIEGLIFLLISAIGLRAKLAIGLFLAFI

LQNNQGIGLVGYSSSTLVTLAPVITLGIVGFVIISY

YGIVFVTAVSWFRNTSVTAFRKVVDVHVIKSTVDTTGTLYSMARFAGFT

DADAASIVVGSLLGTSPVTVESSTGIREGGRTGLTSLTVAGYFFLALFFTPLLA

SIPAWAVGPPLILIEWNDMKQAIPAFVTLILTPLTYS

>NNU_24425RA

IMGVFANLPLALAPGMGTKSALAAVFIEGLIFLLISAIGLRTKLAIGLFLAFI

LQNNQGIGLVGYSSSTLVTLAPVITLGIVGFVIIAY

YGILFVTAVSWFRNTRVTAFKKVVDVHAIESTADTTGTLYSMARFAGFM

DADAASIVVGSLLGTSPVTAESSTGIREGGRTGLTALTVAGYFFMAFFLTPLLA

SIPAWAVGPPLILIEWNDMKQAIPAFVTLILMPLTYS

>Potri.016G133800.1

IMGLLANLPLALAPGMGTKSALTAIFIEGLVFLFISSIGLRAKLAIGLFLAFI

LQNNQGIGLVGYSSSTLVTLAPVVMLGILGFVIIAY

YGIVIVTVISWFRNTAVTAFKKVVDVHVIEKTADTTGTLYSMAKFAGFT

DVDAASIVVGSLLGTSPVTTESSTGIREGGRTGLTALIVAGYFFMAFFFTPLLA

SIPAWAVGPPLILIKWDDMRQAIPAFITMIMMPLTYS

>Solyc06g050250.1.1

IMGVLANLPLALAPGMGTESALAAVFIEGLLFLLISAIGLRAKLAIGLFLSFI

LQNNQGIGLVGYSPSTLVTVAPVMALGIVGFVIIAY

YGIIFVTAISWFRNTSVTAFKKVVDVHTIKTTADTTGTLYSMARFAGFT

DEDASAIVVGSLLGTSPVTAESSTGIKEGGRTGMTALTAAGYFFLAFFFTPILA

SIPAWAVGPPLILVEWDDMRQAIPAFVTLILTPLTYS

>GSVIVT01030208001

IMGTFANLPLALAPGMGTSSALAAIFIEGLIFLFISAVGLRARLAI

VTLGM

KKIVDVHAIESTADTTGTLYSMARFAGFT

DDDAASIVVGSLLGTSPVTTESSTGIREGGRTGLTALTVAGYFFMAFFFTPLLA

SIPAWAVGPPLILIEWDDMKQAIPAFVTMLLMPLTYS

>el__p5_sc00007.V1.gene266

IMGAFANLPLAVAPGMGTGNALAAVFLEGLLFLLLAALGFRSKLAIGLFLAFI

LQNNEGVGLVGYSSSTLVTLAPVRSLAVVGFLIIAF

YGIIFVTAVSWFRGTAVTAFKKVVDVHPIRSTADTTGTLYSMARFAGFV

DEDATAIVAGSLLGTSPVTAESSTGIREGGRTGLTALTVAGYFFLAFFFTPLLA

SIPAWAVGPPLVLIDWEDMKEAIPAFMTLLLMPLTYS

>el__p5_sc00033.V1.gene306

IMGAFANLPLALAPGMGAGNALAAVFLEGLLFLLISALGFRSKLAIGLFLAFI

LQNDQGVGLVGYSSSTLVTLAPVRSLAVVGFLIIAF

YGIVFVTAVSWFRGTAVTAFKKVVDIHPIRSTADTTGTLYSMARFAGFV

DEDATAIVAGSLLGTSPVTAESSTGIREGGRTGLTALTVAGYFMLSFFFTPLLA

SIPAWAVGPPLVLIDWDDMKEAIPAFMTLILMPLTYS

>GSMUA_Achr10P12940_001

IMGAFANLPLALAPGMGTRTALAAVFLEGLLFLLISALGLRARLAIGLFLAFI

LQNNEGVGLVGYSSSTLVTLAPLAVVGFLIMAF

YGIVFVTAVSWFRHTSITAFKKVVD

DAAAIVVGALLGTSPVTAESSTGIKEGGRTGMTALTVAGYFLLAFFFTPLLA

SIPAWAVGPPLVLIEWEDMKEAIPAFMTMLLMPLTYS

>GSMUA_Achr6P15800_001

IMGAFANLPLALAPGMGTRTALAAVFLEGLLFLLISALGFRARLA

LAPLAVVGFLIIAF

YGIVFVTAVSCFKGIGTGRF

DAVAIVVGSLLGTSPVTAESSTGIKEGGRTGMTALTVAVYFLLAFFFTPLLA

SIPAWAVGPPLVLIEWEDMKEAIPAFMTILLMPLTYS

>GSVIVT01032706001

IMGAFANLPLGLAPGMGTQNALAAVFIEGLIFLLISAVGLRTKLAIGLFLAFI

LQNNQGIGLIGYSSATMV

>LOC_Os05g26840.1

IMGTFANLPIALAPGMGTRTALAAVFLEGLIFLFISLVGLRSKLAIGLFLAFI

LQSSEGVGLVGFSSSTLVTVAPVVTLAVVGFLIIAF

YGILFVTFISWPRNTAVTVFKKVFDVHRIQSTADTTGGLYSMARFAGFV

DDADATAIVFGSLLGTSPVTAESSTGIREGGRTGLTALTAAAYFAAALFVTPLLA

SIPSWAVGPPLVLVDWADMRQAVPAFLTLALMPLTYS

>Sobic.009G097100.1.p

IMGAFANLPIALAPGMGTRKALAAVFLEGLIFLLISIVGLRSKLAIGLFLAFI

LQSNEGVGLVGFSSSTLVTVAPVLTLAVVGFLIIAF

YGILFVTFVSWPRHTAVTAFKKVFDVHRIRSTADTTGSLYTMARFAGFV

DDVDATAIVFGSLLGTSPVTAESSTGIREGGRTGLAALTTAAYFAAALFITPVLA

SIPSWAVGPPLVLVDWDDMRQAVPAFLTLALMPLTYS

>Seita.3G284700.1.p

IMGAFANLPIALAPGMGTRTALAAVFLEGLIFLFISVVGLRSKLAIGLFLAFI

LQSNEGVGLVGFSSSTLVTVAPVVTLAVVGFLIIAF

YGILFVTFVSWPRGTAVTAFKKVFDVHRIRSTADTTGGLYSMARFAGFV

DDADASAIVFGSLLGTSPVTAESSTGIREGGRTGLTALTAAVYFAAALFITPLLA

SIPSWAVGPPLVLVDWNDMRQAVPAFLTLALMPLTYS

>Zm00001d000434_T001

IMGAFANLPIALAPGMGTRTALAAVFLEGLIFLFISIVGLRSKLAIGLFLAFI

LQSNEGVGLVGFSSSTLVTVAPVLTLAVVGFLIIAF

YGILFVTFVSWPRHTAVTAFKKVFDVHRIRSTADTTGSLYTMARFAGFV

DDADATAIVFGSLLGTSPVTAESSTGIREGGRTGLTALTAAIYFAAALFITPVLA

SIPSWAVGPPLVLVDWDDMRQAVPAFLTLALMPLTYS

>Bradi2g30850.1.p

IMGAFANLPIALAPGMGTRTALAAVFLEGLIFLFISVVGLRSKLAIGLFLAFI

LQSSQGMGLVGFSSSTLVTVAPVMTLAVVGFIIIAL

YGILFVTFISWPRNTAVTVFKRVFDVHRIRSTADTTGGLYSMARFAGFM

DDADATAIVFGSLLGTSPVTTESCAGIREGGRTGLTALTAAAYFMAALLVTPLLA

SIPSWAVGPPLVLVDWADMRQAVPAFMTLALMPLTYS

>126004

IMGVFANLPLGLAPGMGAGGALAAVFVEGLIFLGLAALGLRTRLAIGVFLTLI

LQSSEGVGL

>419549

IMGVFANLPLGLAPGMGAGGALAAVFVEGLIFLGLAALGLRTRLAIGVFLTLI

LQSSEGVGLIGFSPSSLVTVAPVVTNLGVVGFVIIAY

YGIVFVTGISWFRGTKVTYFRKVVDVHTIKGTADTTGTLYSMAKFAGFV

DEDAAAIVVGSALGTSPVTAESSTGIREGGRTGITALAVAMYFFLALFFTPLLA

SIPPWAVGPALVLIDWDDMREAIPAFITIVLMPLTYS

>419524

IMGVFANLPLGLAPGMGAGGALAAVFVEGLIFLGLAALGLRTRLAIGLFLAFI

LQSSEGIGLIGFSPSTLVTVAPVVTNLGVVGFVIIAY

YGIVFVTGISWFRGTQVTYFREVVDVHTIKGTADTTGTLYSMAKFAGYV

DEDAAAIVVGSALGTSPVTAESSTGIREGGRTGITALTVAMYFFLALFFTPLLA

SIPPWAVGPALVLIEWEDMREGIPAFITIILMPLTYS

>Pp3c19_3540V3.1.p

IMGMFANLPLGLAPGMGTQSALAAVLIEGIIFLVIAALGFRTKIAIGLFLAFI

LQASEGVGLVAYDGSTLLTLAPVILAVVGFLIIAY

YGIVFVTGVSWFRHSKVTYFKNVVDAHKIQLTADTTGTLFTMAKFAGFV

DADATAICAGSLLGTSPITTESSTGIREGGRTGLTAITVAFYFFLSLFFTPLLA

SIPPWAVGPALVLINWEDLREAIPAFITIIVMPLTYS

>AT5G50300.1

AMGLLANLPFGLAPGMGAHTAMAIVLLEGCAFLAVSALGLRGKLAIGMFIAFV

LQMNQGIGLVGPDKSTLVTLAVVGFLITSF

YGIVFVTAISWIRGTQVTIFTKIVDFHKIQSTLGTTGVLYTMAEIGGFV

EDDAGSSVVGSALGVTTTATESSAGLKEGGKTGLTAVIVGLYFLASMFFTPLVT

NVPRWAVGPSLVMIRWGETKEAVTAFVTILLMPLTYS

>Potri.015G090000.1

AMGILANLPFGLAPGMGPKTAMAIVLVEGCAFFIVSALGLREKFAIGLFIAFV

LQIHQGVGLVGPDQSTLVTLGSVGLLITCY

YGILFVTLTSWIRGTAVTYFKKVVDFHKIQSTAGTTGILYTMAEIGGFV

NEDAGSTIVGSTLGVTTTATESSAGIREGGRTGLTAVTVGFYFLVSLFFTPLLT

SVPPWAIGPSLVMIDWENIKEAVPAFITMLLMPLTYS

>Thecc1EG014561t1

AMGILANLPLGLAPGMGPQTALAVVLVEGCAFLAIAALGLRAKLAIGLFIAFV

LQIHQGLGLVGPDPSTLVTLGFAGFLITCY

YGILFVTLISWIRGTAVTYFKKVVDFHKIQSTAATTGTLYTMAEIGGFV

DDDSSSTVMASALGVSPVATESSAGIKEGGRTGLTAVIVGVYFFLSLFFTPLLT

SVPPWAIGPSLVMINWGNMKEAAPAFVTMLLMPLTYS

>Solyc03g111400.1.1

AMGLLANLPLGLAPGMGPQTVMAIFLVEGCAFLAIAVFGLRGRIAIGLFIAFV

LQAHQGVGLVGPDPSTLITLGSVGFIIMCY

YGILFVTLISWIRNTAVTVFKKVVDFHKIESTAASTGTLYTMAEIGGFV

NEDAGSTIVASTLGVSPVATESSAGIREGGRTGITAIVVGFYFLLSLFFTPLIA

SVPPWAIGPSLVIIDWNNIKHAVPAFVTMVLMPLTYS

>PGSC0003DMP400026721

AMGLLANLPLGLAPGMGPQTVMAIFLVEGCAFLAIAVFGLRGRIAIGLFIAFV

LQAHQGVGLVGPDSSTLITLGSVGFIIMCY

YGILFVTLISWIRNTAVTVFKKVVDFHKIESTAASTGTLYTMAEIGGFV

NEDAGSTIVASTLGVSPVATESSAGIREGGRTGITAIVVGFYFLLSLFFTPLIA

SVPPWAIGPSLVIIDWNNIKHAVPAFVTMVLMPLTYS

>NNU_02168RA

AMGLLANLPFALAPGMGVQTALAIVFVEGCIFLVISALGLRAKLAIGLFIAFV

LQPHQGIGLVGPSSSTLLTLGAVGFLITCY

YGILFVTLISWIRGTSVTYFKKVVDFHRIKTTAATTGILYSVAQVGEFV

DDEDASSTIVGSALGTSTMATESSAGIREGGRTGLTALTVGFYFLLSLFFTPLLA

SVPPWAIGPSLVMIEWMKVKEGAPAFVTMLLMPLTYS

>NNU_22058RA

AMGLLANLPFALAPGMGVQTALAIVLVEGCVFLAIAAFGLRARLAIGLFIAFV

LQSQQGVGLVGPSSSTLVTLGAVGFLVTCY

YGILFVTLVSWIRGTSVTYFKQVVEFHKIKSTAATTGTLYSVAEVGEFI

DEDAGSTVVGSALGVSPIATESSAGIREGGRTGLTALVVGFYFFLSLFFTPLLA

SVPPWAIGPSLIMIEWVKVKEGAPAFVTMMLMPFTYS

>LOC_Os11g24060.1

AMGALANLPLGLAPGMGASTALAGVMVEGIVFFILSAVGLRSRLAIGLFLAFT

LQAHQGVGLVGASPSTLVTLGAVGFLITAT

YGIVFVTVVSWIRGTAVTVFKKVVDFHTITTTAATTGTMYSMAEYGGFT

DGADAGSTVLSAALGSTTVTTESTAGIREGGRTGVTAITVAACFLASLFFGPVLT

SVPPWAVGPSLVLIEWADMKEAIPAFVTMVLMPLTFS

>Bradi4g19930.1.p

AMGALANLPLALAPGMGARTALAGVMLEGIVFFFLSAVGIRSRLAIGLFLAFT

LQANQGVGLVGSSPSTLVTLGVAGFLVTAT

YGIVAVTAVSWVRGTSVTVFKKVVDFHMIGTTADTTGTMYSMAEYGGFT

DGDAGSTVLSAALGSTTVTTESTAGIREGGRTGVTAITVSACFLASLFFSPLLT

SVPPWAVGPSLVLIEWGDMKEAIPAFVTMALMPLTFS

>Sobic.005G091500.1.p

AMGVFANLPLALAPGMGARTALAVVMLEGLVFFALSAVGLRSRLAIGLFLAFT

LQAHQGVGLVGASPSTLVTLGAVGFLITAT

YGILFVTVVSWIRGTSVTVFKKVVDFHMIRTTADTTGTMYSMAEYAGFV

DEDDAGSTVLSAGLGSSTVTTESTAGIREGGRTGITAVTVAACFLASLFFGPLLM

SVPPWAVGPSLVLIEWGDMKEGVPAFVTMALMPLSFS

>Zm00001d048960_T001

AMGLFANLPLALAPGMGARTALAVVMLEGLVFFALSAVGLRSRLAIGLFLAFT

LQAHQGVGLVGASPSTLVTLGAVGFLITAT

YGILFVTVVSWIRGTSVTVFRKVVDFHVIRSTADTTGTMYSMAEYGGFV

KEDAGSTVLSAGLGSSTVTTESTAGIREGGRTGLTAITVAACFLASLFFGPLLM

SVPPWAVGPSLVLIEWGDMKEGVPAFVTMALMPLSFS

>Seita.2G153000.1.p

AMGLFANLPLALAPGMGASTALAVVMLEGIVFFALSAVGLRSKLAIGLFLAFT

LQAHQGVGLVGASPSTLVTLGAVGFLITAT

YGILFVTVVSWIRGTSVTVFKKVVDFHMIKSTADTTGTMYSMAEYGGFT

DEDAGSTVLSAGLGSSTVTTESTAGIREGGRTGLTAITVAACFLASLFFGPLLM

SVPPWAVGPSLVLIEWGDMKEGVPAFVTMALMPLSFS

>Zm00001d007251_T001

AMGLFANLPLALAPGMGASTALAVVMLEGLVFFALSVVGLRSRLAIGLFLAFT

LQAHQGVGLVGASPSTLVTLGAVGFLITAT

FGMLFVTVVSWIRGTSVTMFKKVVDFHMIRSTADTTGTMYSMAEYGGFT

DGADAGSTVLSAGLGSSTVTTESTAGIREGGRTGLTAITVAACFLASLFFGPLLM

SVPPWAVGPSLVLIEWGDMKEGVPAFVTMALMPLSFS

>GSVIVT01007551001

FMGVLANLPLAVAPAMGPGTGLAVLCVEGLAFIFLSASGLRAKVAIGLFIAFV

LQAHQGVGLVGPDPSTLLGMVGLVITC

TAVTYFKKVATTGILYTMAELGGFV

DDDAGTTVVASTLGGSPVATESSAGLREGGRTGLTAVIVSFYFMLSLFFTPLLT

SVPPWAIGPSLVMIEWGNVKDAVPAFATMVLMPLTYS

>GSMUA_Achr10P16670_001

AMGSFANLPLALAPGMGTGTALAAVMLEGCLFLALSVLGLRAKLAIGLFLAFT

LQANQGVGLVGPLGAAGFLVTAA

YGIVFVTLISWIRGTSDTTGSMYSMAD

STIVGSALGTTTVTTESTAGLREGGRTGLTAITVALLFLVSLFFAPLFT

NVPPWAIGPSLVLIEWSDAKTAVPAFLTLILMPLTYS

>GSMUA_Achr6P06790_001

AMGSFANLPLALAPGMGAETAMAAVMLEGCLFLALSVLGLRAKLAIGLFLAFT

LQAREGIGLVGPSKSTLVT

YGIVIVTLVSWFRGTSNTADTTGSMYSMAD

ATIVGSALGTTTVTTESTAGLREGGRTGLTAITVAALFLVSAFFTPLLT

NVPPWAVGPSLVLIEWVEMKEAVPAFLTMILMPLTYS

>GSMUA_Achr6P12090_001

AMGSFANLPLALAPGMGAETALAAVMLEGCLFLALSVLGLRSKLAIGLFLAFT

LQAREGVGLVGPSSSTLVT

YGVVFVTLVSWIRGTSSTADTTGSMYSMAEYGGFT

DDDASATIVGSALGTTTVTTESTAGLREGGRTGLTAITVAFLFLVSLFFAPLFT

NVPPWAVGPSLVLIEWSETKEAVPAFLTMILMPLTYS

>Cre06.g260700.t1.2

LMGVVGNLPFGLAPGMGIQDALAAAFIEGWIFFVISISGLRGKITIGIFLAFI

MQTSNGIGLIAFEPATLVTYTIADPAGVCSLLGICGGILMVL

VAILFVTFISWIPNHDASYLGAKKVVQVPNTSATDDCTGTFYSMAAYIDKRQPGFI

NPIDATAIWVGALLGIPPLTTESATGIREGGRTGITAIMIGFYFFLAMFFTPIIS

SIPPYATGPALILIDWKDYTQAIPAFITISVIPLTYS

>Cre10.g448450.t1.1

TAECLGTEKALAATFLEGLVFLAICFLGIRRWLLIGCFISFV

VKDMGVIVAAPYPTLLSLGIPLSVGGLIFTAIVS

MGIFFTMFISWIKFPEKISTGQGKVAYLPKFQETAGSCITFVAMGEMTGIL

DEDGFGTMLGGLLGSSALTTESASAVREGGRTGITAIVCALFFFAACFLSPLFS

VIPAIATGPILALINWHDITDAIPAFVTMLGMPFTHN
